# Ultralight Conductive MXene Aerogels: From Material Design to Energy, Environment, and Electronic Applications

**DOI:** 10.1002/smsc.70287

**Published:** 2026-04-21

**Authors:** Narasimharao Kitchamsetti, HyukSu Han, Sungwook Mhin

**Affiliations:** ^1^ Department of Microsystems University of South‐Eastern Norway Borre Norway; ^2^ Division of Materials Science and Engineering Hanyang University Seoul Republic of Korea; ^3^ Department of Energy and Materials Engineering Dongguk University Seoul Republic of Korea

**Keywords:** catalysis, electromagnetic interference shielding, energy storage, MXene‐based aerogels, sensors and solar desalination, water purification

## Abstract

MXene‐based aerogels have emerged as a fast‐growing family of porous materials that merge the intrinsic electrical conductivity and layered morphology of MXenes with an ultralight, great surface‐area framework of aerogels. This synergistic integration results in materials that exhibit excellent charge and heat transport, mechanical flexibility, and broad multifunctionality. This review summarizes the fundamental design strategies underlying MXene aerogels, with particular attention to how pore structure, architectural organization, and surface functionalization govern their performance. Advanced characterization techniques are highlighted for their role in revealing structure‐property correlations. Key application areas are surveyed, spanning energy storage, sensing technologies, environmental remediation (including solar desalination and water treatment), catalysis, electromagnetic interference shielding, and thermal regulation. The review also identifies critical barriers to practical implementation, such as environmental susceptibility, degradation mechanisms, and performance trade‐offs between conductivity and other functional attributes. Finally, future prospects are discussed, emphasizing AI‐enabled materials design, greener synthesis strategies, scalable manufacturing, and integration into next‐generation technologies, including soft robotics, neuromorphic systems, and bioelectronic platforms.

## Graphical abstract

MXene aerogels combine the intrinsic conductivity and surface chemistry of MXenes with ultralight, highly porous 3D architectures, enabling efficient charge/heat transport and multifunctional performance. By tailoring pore structure, orientation, surface functional groups, and hybrid compositions (with polymers, carbons, metals, and MOFs), MXene aerogels offer tunable properties for diverse applications including energy storage, catalysis, desalination, water treatment, sensing, electromagnetic interference shielding, thermal management, and biomedicine. This review highlights design principles, structure‐property relationships, and emerging opportunities for next‐generation MXene aerogel systems.

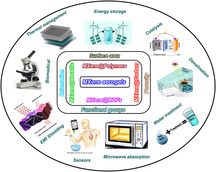

## 1 Introduction

MXene‐based aerogels have attracted increasing attention as a multifunctional materials class capable of meeting a wide range of technological requirements, including energy storage, environmental remediation, sensing, and biomedical applications [1, 2, 3]. The integration of the high electrical conductivity and rich surface chemistry of two‐dimensional (2D) MXenes with the lightweight and highly porous architecture of aerogels yields 3D conductive frameworks with low density and adjustable surface functionality. Such structures support diverse applications, spanning electrochemical storage, water treatment, electromagnetic interference (EMI) shielding, and biosignal monitoring [4, 5]. As a result, MXene aerogels constitute a promising platform for the design of next‐generation lightweight and multifunctional devices. MXenes themselves serve as the fundamental building blocks of these aerogels and comprise a broad family of 2D transition‐metal (TM) carbides, nitrides, and carbonitrides [6, 7, 8]. MXenes were 1^st^ introduced in 2011 through selective removal of Al layers from titanium carbide MAX phases. These materials exhibit metallic‐level electrical conductivity and intrinsically hydrophilic surfaces terminated with functional groups for instance ^‐^O, ^‐^OH, and ^‐^F [9, 10, 11]. As a result, MXenes possess high electrical conductivity and abundant active sites, enabling their use in electrodes, EMI shielding, sensing devices, and biocompatible systems [12]. In contrast, aerogels are ultralight solid materials produced by replacing the liquid phase of a wet gel with gas while preserving the 3D porous framework. Historically, aerogels have been defined as highly porous solids (porosity exceeding 90%) produced under supercritical conditions through supercritical drying. However, advances in alternative fabrication methods, such as freeze drying and ambient‐pressure drying, have broadened this definition to encompass a wide range of inorganic, carbon (C)‐based, and polymeric aerogels [13]. Silica aerogels exemplify both the advantages and limitations of conventional aerogels, exhibiting extremely low density and thermal conductivity while suffering from poor mechanical robustness [14]. Incorporating MXenes into aerogel architectures mitigates this mechanical fragility and simultaneously enhances electrical conductivity and functional diversity.

The preparation of MXene aerogels generally begins with the exfoliation of layered MAX precursors to obtain negatively charged MXene nanosheets (NSs). These NSs are then organized in 3D porous networks using techniques such as sol–gel routes, freeze‐casting, chemical crosslinking, or combinations thereof [15]. Control over porosity and structural organization, ranging from macroporous to mesoporous networks and from isotropic to unidirectionally aligned architectures, enables precise tuning of mechanical properties, mass transport behavior, and electrical conduction pathways. In addition, surface functionalization strategies allow modification of MXene flakes or the incorporation of polymers, C‐based materials, metal nanoparticles (NPs), and metal‐organic frameworks (MOFs) onto their surfaces. Aerogels composed solely of MXenes retain high electrical conductivity; however, they are typically formed from stacked flakes and lack elastomeric components, which limits their mechanical resilience. Incorporating graphene, reduced graphene oxide (rGO), C nanotubes (CNTs), or amorphous C yields composite aerogels with improved structural tunability and mechanical robustness [16, 17, 18]. To further enhance flexibility and biodegradability, polymeric binders such as cellulose, chitosan, polyimide, and polylactic acid (PLA) can be introduced. For instance, a biodegradable MXene‐tissue paper aerogel was fabricated by sandwiching a MXene layer between PLA NSs, enabling force sensing up to 30 kPa with stable performance under minimal power operation [19, 20]. Additionally, hybrid aerogels combining high electrical conductivity with specific chemical functionality can be realized by incorporating catalytic or adsorption sites, including metal NPs or MOFs.

The versatility of MXene aerogels is evident from the wide range of applications they support. Their exceptional conductivity, high specific surface area (SSA), and hierarchically porous frameworks make them highly suitable as electrode materials for supercapacitors (SCs) and batteries. In particular, composite MXene aerogels effectively suppress NSs restacking, resulting in enhanced capacitance and high‐power density [21, 22, 23]. Beyond energy storage, their hydrophilic surfaces and tunable interlayer spacing facilitate selective ion transport and adsorption, enabling applications in water treatment and desalination. MXene aerogels have been integrated into membrane‐based systems such as capacitive deionization, ion desalination, and solar‐driven purification, where their porous structures and strong photothermal response improve solar absorption and steam generation efficiency [24, 25]. In sensing technologies, MXenes display a strong piezoresistive response, whereby applied mechanical stress modifies charge transport pathways and interlayer distances, resulting in detectable variations in electrical resistance. This property underpins the design of highly responsive pressure and strain sensors based on MXene aerogels. For instance, MXene/rGO aerogel sensors exhibit sensitivities on the order of 22 kPa^−1^ with response times shorter than 200 ms, whereas biodegradable MXene‐tissue paper sensors can detect pressures down to 10 Pa and are readily adaptable to wearable motion‐monitoring systems [26, 27]. In addition, textile‐integrated sensors produced by dip‐coating cotton fibers with MXenes achieve gauge factors of approximately 6 and retain stable performance over thousands of loading‐unloading cycles, demonstrating their potential for electronic skins and smart healthcare textiles [28]. Beyond mechanical sensing, MXene aerogels are also being explored for gas detection, biosensing, and photothermal therapeutic applications, owing to their high SSA and redox‐active surface chemistry.

Further MXene aerogels exhibit outstanding performance in EMI shielding and thermal management applications. Their high metallic conductivity, layered architectures, and porous frameworks promote multiple internal reflections and efficient absorption of incident electromagnetic (EM) radiation. For example, nacre‐inspired MXene/cellulose aerogels demonstrate EMI shielding effectiveness (SE) values ranging from 35.5 to 74.6 dB at ultralow densities of 1.5–8 mg cm^−3^ [29]. In addition to EMI shielding, these aerogels provide effective thermal insulation, making them attractive for electronic packaging and wearable devices. Conductive MXene frameworks, either alone or combined with magnetic or dielectric fillers, can also function as broadband microwave (MW) absorbers. Moreover, MXene aerogels exhibit excellent electrical conductivity and a high density of accessible active sites, making them attractive platforms for catalytic processes. The introduction of metal NPs or heteroatom dopants creates synergistic interfacial effects that enhance catalytic reactions such as the oxygen reduction (ORR), hydrogen evolution (HER), and carbon dioxide reduction (CO_2_RR). In addition, coupling MXene aerogels with phase‐change materials (PCMs) enables simultaneous thermal storage and temperature regulation [30].

Despite the rapid growth of research on MXenes and aerogels, existing review articles have primarily addressed these materials either independently or with a limited focus on specific applications. A comprehensive framework that integrates the unique structural features of MXenes with aerogel architectures, while systematically correlating design parameters with multifunctional performance, remains insufficiently developed. In this context, the present review distinguishes itself by providing a unified perspective that bridges material design, structural engineering, and application‐driven performance of MXene‐based aerogels. Particular emphasis is placed on the interplay between pore architecture, surface chemistry, and hybrid composition and how these factors collectively govern electrical, mechanical, thermal, and electrochemical properties. Furthermore, this review goes beyond conventional summaries by critically analyzing performance trade‐offs, durability challenges under realistic environmental conditions, and scalability considerations. In addition, emerging directions such as bioinspired assembly strategies, sustainable material integration, and data‐driven approaches including machine learning (ML)‐assisted materials design are highlighted to provide forward‐looking guidance. Through this integrative and application‐oriented perspective, the present work aims to offer a clearer roadmap for the rational design and practical implementation of next‐generation MXene aerogels.

## 2 MXenes: Tunable High‐Performance 2D Material

2D materials are a class of atomically thin solids in which strong covalent bonding exists within the basal plane, while adjacent layers are held together by relatively weak interactions [31]. The combination of minimal thickness and large lateral dimensions results in a high SSA and a substantial fraction of exposed atoms, leading to distinctive mechanical and electronic properties, including high stiffness, mechanical strength, and intrinsic flexibility, compared with their bulk counterparts [32]. Following the successful isolation of graphene, a wide range of additional 2D materials have been developed whose ultrathin architectures have enabled diverse applications in areas such as energy storage, sensing, and photonics. Among the various 2D materials, MXenes represent a recently identified family. They were first discovered in 2011 [33] following the selective etching of Ti_3_AlC_2_, a MAX‐phase compound, to achieve Ti_3_C_2_ layers. MXenes are composed of 2D NSs of early TM carbides, nitrides, or carbonitrides, in which metal layers are coordinated with C or nitrogen (N) atoms and terminated by surface functional groups such as ^‐^O, ^‐^OH, and ^‐^F [34, 35]. Their parent MAX phases follow the general formula M_*n + 1*_AX_*n*_, where M denotes an early TM, A corresponds to a group 13 or 14 element, and X represents C or N. The robust M‐X bonds combined with relatively weak M‐A bonds enable selective removal of A layer through chemical etching (e.g., using HF), yielding thin M_*n + 1*_X_*n*_ NSs with functionalized surfaces. These terminations impart hydrophilicity, high conductivity, mechanical robustness, and optical activity to MXenes [36, 37]. Structurally, as‐prepared MXenes typically exhibit an accordion‐like multilayer morphology, which can be further delaminated into few‐layer or monolayer forms [38, 39].

**FIGURE 1:**
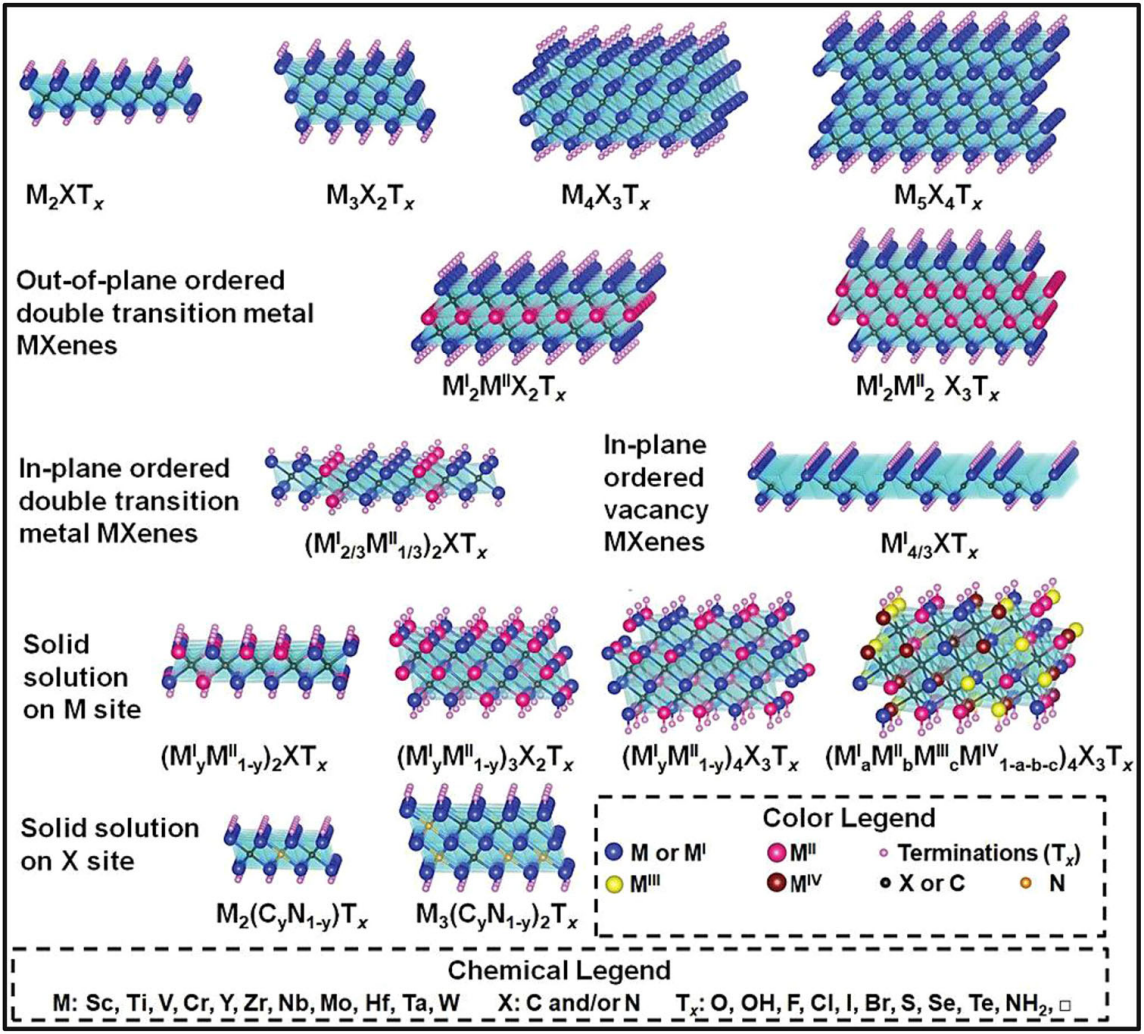
Schematic illustration of various MXene architectures. Adapted from [42]. Copyright 2021, Wiley‐VCH.

MXenes are commonly described by the general formula M_*n + 1*_X_*n*_T_*x*_, where surface functional groups arise from synthesis processes. In single TM systems, representative compositions such as M_2_C, M_3_C_2_, and M_4_C_3_ are derived directly from their parent MAX phases. Structurally, MXenes comprise closely stacked TM layers, with X atoms occupying octahedral coordination sites and metallic A layers inserted between them. Because the bonding between the M and A layers is relatively weak, selective etching removes the A component, yielding layered M_*n + 1*_X_*n*_ NSs that are held together primarily by van der Waals forces and H‐bonding. These weak interactions facilitate subsequent exfoliation into few‐layer or monolayer sheets through intercalation or ultrasonic treatment [40, 41]. The simultaneous presence of metallic M–M bonding within TM layers and strong covalent M–X interactions gives rise to the distinctive property profile of MXenes. As a result, these materials demonstrate excellent electrical conductivity, robust mechanical characteristics such as high Young's modulus, stiffness, and tensile strength, and pronounced electromechanical responses (Figure 1). The large number of possible combinations of TMs, X elements, and layer thicknesses (n) places MXenes among the most compositionally diverse 2D material families, surpassing traditional systems such as graphene, MoS_2_, h‐BN, and black phosphorus [42, 43]. To date, over 30 MXene compositions have been synthesized experimentally, with many more predicted to be stable based on theoretical calculations. Importantly, properties including work function, band structure, and charge transport can be deliberately tailored by modifying the metal chemistry, X species, or surface terminations [44, 45].

**FIGURE 2:**
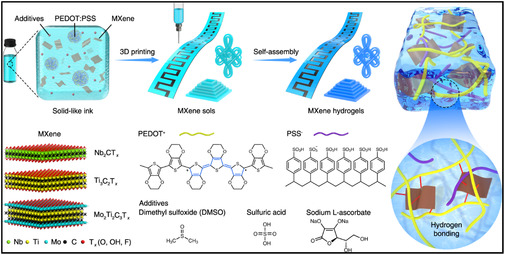
Hybrid inks containing MXenes, PEDOT: PSS, and various additives (DMSO, H_2_SO_4_, and sodium L‐ascorbate) are 3D printed into predetermined geometries and then undergo self‐assembly, during which MXene sols are transformed into MXene hydrogels. The MXene systems Nb_2_CT_*x*_, Ti_3_C_2_T_*x*_, and Mo_2_Ti_2_C_3_T_*x*_ are employed to demonstrate the broad applicability of this approach. Adapted from [54]. Copyright 2022, Springer Nature.

MXenes exemplify how a conceptually modest 2D material can be engineered to a highly tunable platform with multifunctional and adaptive frameworks. The presence of robust M‐X bonding within the atomic layers, together with chemically active surface terminations, enables MXenes to readily interact with a wide range of species [46, 47]. This rich surface chemistry supports diverse assembly approaches that transform individual MXene NSs into robust 3D frameworks. By cross‐linking or templating MXene layers into porous architectures, van der Waals driven restacking, commonly observed in many 2D materials, can be effectively suppressed. These 3D networks preserve the intrinsic conductivity and mechanical integrity of MXene monolayers while providing increased accessibility to active sites for ion and molecular transport [48]. For instance, porous MXene‐GO composite films can be fabricated by cross‐linking Ti_3_C_2_T_*x*_ NSs with GO scaffolds during vacuum‐assisted filtration, yielding a disordered 3D network that suppresses NSs restacking [49]. Alternatively, MXene coatings deposited on sacrificial polymer templates can be removed to generate hollow spheres or macroporous films. In such architectures, both wall thickness and mechanical compliance are tunable through adjustment of the MXene‐to‐template ratio, resulting in structures with high conductivity and facilitated ion diffusion [49, 50]. Hydrazine‐induced foaming expands the interlayer distance of vacuum‐assembled MXene films, producing ultralight foam structures in which density, thickness, and conductivity can be accurately controlled. These materials exhibit hydrophobicity and form continuous cellular networks, which contribute to outstanding EMI shielding performance [51]. In wet‐chemical processing, cross‐linking agents like ethylenediamine, divalent cations, cellulose, or polyimide can interconnect neighboring MXene NSs, yielding mechanically robust hydrogels, aerogels, or monolithic structures. After subsequent drying, these hydrogels transform into foams or dense monoliths with controllable porosity, high structural stability, and potential biocompatibility for applications such as drug delivery and photothermal therapy [52]. Moreover, soft‐templating strategies using emulsions or micelles enable MXenes to assemble at oil–water interfaces. After freeze‐drying, the emulsions are transformed into low‐density aerogels featuring porous, hydrophobic MXene networks with high electrical conductivity [53]. In parallel, additive manufacturing enables accurate control over 3D architectures through the printing of MXene‐based inks combined with conductive polymers (e.g., PEDOT) and functional additives, producing microlattices and micro‐SCs with complex geometries (Figure 2). Thermal treatment of the printed structures induces self‐assembly into cross‐linked MXene hydrogels, yielding programable 4D constructs whose shape, mechanical properties, and electrical behavior can be modulated by controlled thermal cycling. These 4D‐printed MXene hydrogels demonstrate high capacitance, elevated power density, and dynamic shape adaptability [54]. Together, these fabrication strategies highlight how MXene surface chemistry and layered structures facilitate the transition from 2D NSs to 3D multifunctional systems suitable for advanced electrodes, sensors, stimuli‐responsive hydrogels, and high‐performance hybrids.

## 3 Fundamental Concepts Underlying the Design Strategy

### 3.1 Pore Structure and Material Architecture

**FIGURE 3:**
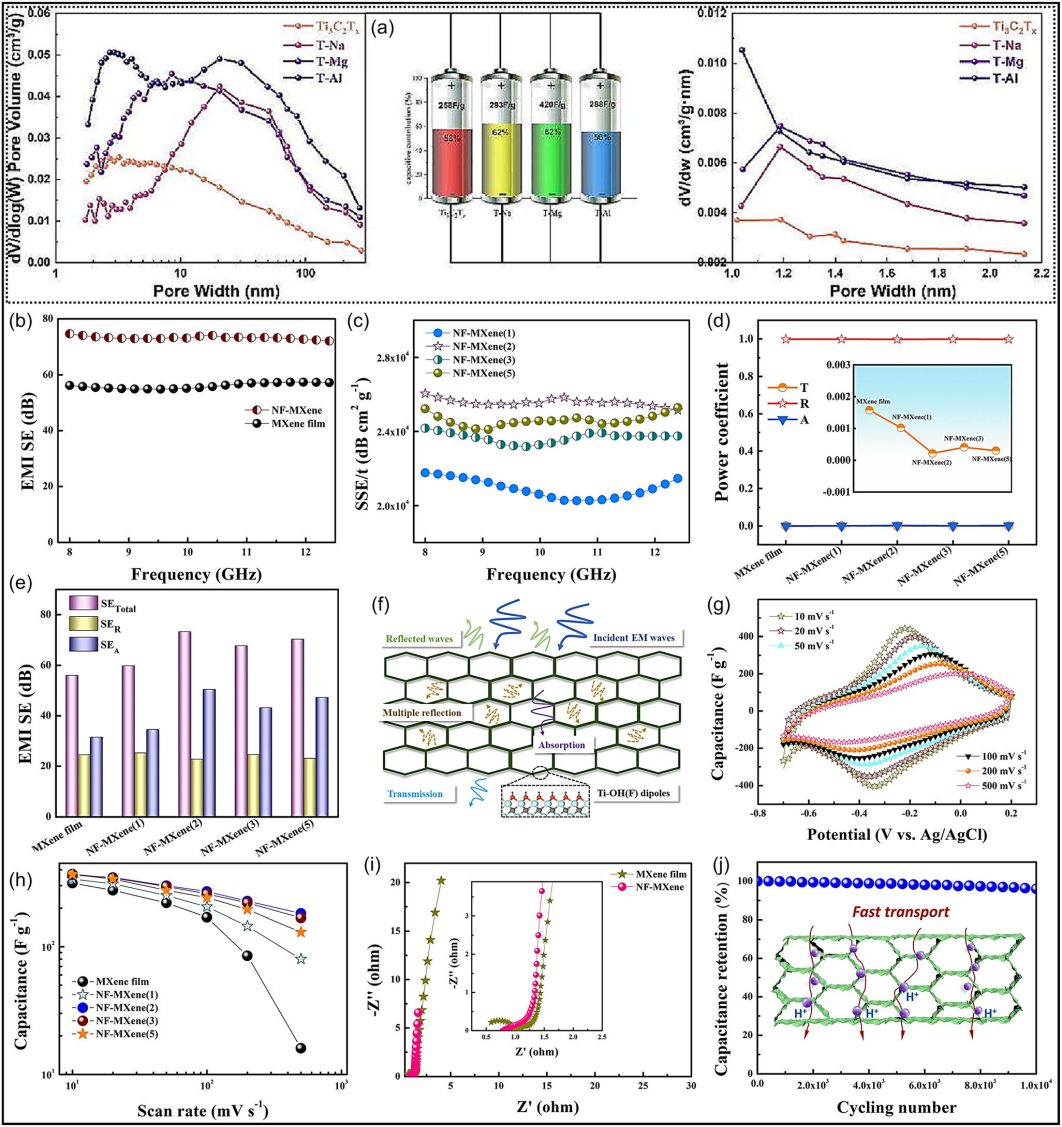
(a) Optimization of pore structure enhances the performance of 3D Ti_3_C_2_T_*x*_ electrodes. Adapted from [57]. Copyright 2023, Elsevier B.V. (b‐j) Electrochemical behavior and EMI shielding characteristics of NF‐MXene: (b) SSE/t of NF‐MXene, (c) comparison of EMI SE between NF‐MXene and MXene films, (d) reflection (R), transmission (T), and absorption (A) coefficients for NF‐MXene versus MXene films, (e) total SE, SER, and SEA comparisons, (f) schematics of possible EM wave paths in NF‐MXene, (g) CV plots of NF‐MXene at sweep rates from 10 to 500 mV s^−1^, (h) capacitance comparison of NF‐MXene and MXene films at different sweep rates, (i) Nyquist plots for NF‐MXene and MXene films with enlarged high‐frequency inset, and (j) capacitance retention of NF‐MXene at 0.2 V s^−1^ over 10 000 cycles with an inset showing ion transport pathways. Adapted from [58]. Copyright 2023, Cell press.

Incorporating internal porosity into MXene‐based assemblies, such as foams and aerogels, effectively mitigates NSs restacking while imparting favorable structural characteristics. Precise control over pore size, spatial distribution, orientation, and hierarchical arrangement plays a critical role in determining overall material performance [55, 56]. In MXene aerogels, pore size must be carefully optimized to balance accessible SSA against ionic transport resistance. When pore dimensions are comparable to the lateral size of Ti_3_C_2_T_*x*_ flakes (≈300 nm), total porosity is reduced while interflake bottlenecks are minimized, yielding shorter diffusion pathways without sacrificing surface exposure. As a result, aerogels templated with pores around 300 nm demonstrate enhanced electrochemical performance, achieving capacitances of 474 F g^−1^ at 2 mV s^−1^. In contrast, mesoporous structures with pore sizes below 50 nm increase SSA but restrict ion movement due to narrowed channels and overlapping electric double layers, leading to elevated resistance and slower kinetics. Excessively large macropores (>0.5 μm), while favorable for ion transport, diminish active surface density and consequently reduce charge storage capacity. Hierarchically organized meso‐macro porous structures offer an effective strategy to overcome these competing effects. In such architectures, macropores on the order of ∼300 nm serve as efficient ion‐transport channels that feed adjacent mesoporous domains where electrochemical reactions occur, while a limited fraction of micropores contributes to charge storage at low rates. Electrochemical behavior is further improved when electronic conduction pathways are spatially coordinated with ion‐transport routes. The introduction of CNT frameworks creates uninterrupted electron networks, enabling capacitances of 462 F g^−1^ at 2 mV s^−1^ and retaining 205 F g^−1^ at 1 V s^−1^. Additionally, synthesis approaches based on metal‐ion‐mediated assembly generate meso‐ and microporous regions that suppress NSs restacking and facilitate rapid ion delivery from macro‐porous reservoirs to active MXene surfaces (Figure 3a) [57]. Fan and colleagues reported that EMI shielding behavior of NF‐MXene is highly sensitive to its pore architecture. A well‐defined, uniform honeycomb‐like structure enhances internal multiple scattering, ohmic dissipation, and interfacial polarization, thereby delivering strong and frequency‐stable shielding across X‐band. In contrast, excessive porosity promotes pore merging and interrupts conductive networks, which reduces attenuation efficiency even at increased thicknesses. These findings highlight that precise control over pore size and connectivity outweighs the benefits of simply thickening the material (Figure 3b‐j) [58].

**FIGURE 4:**
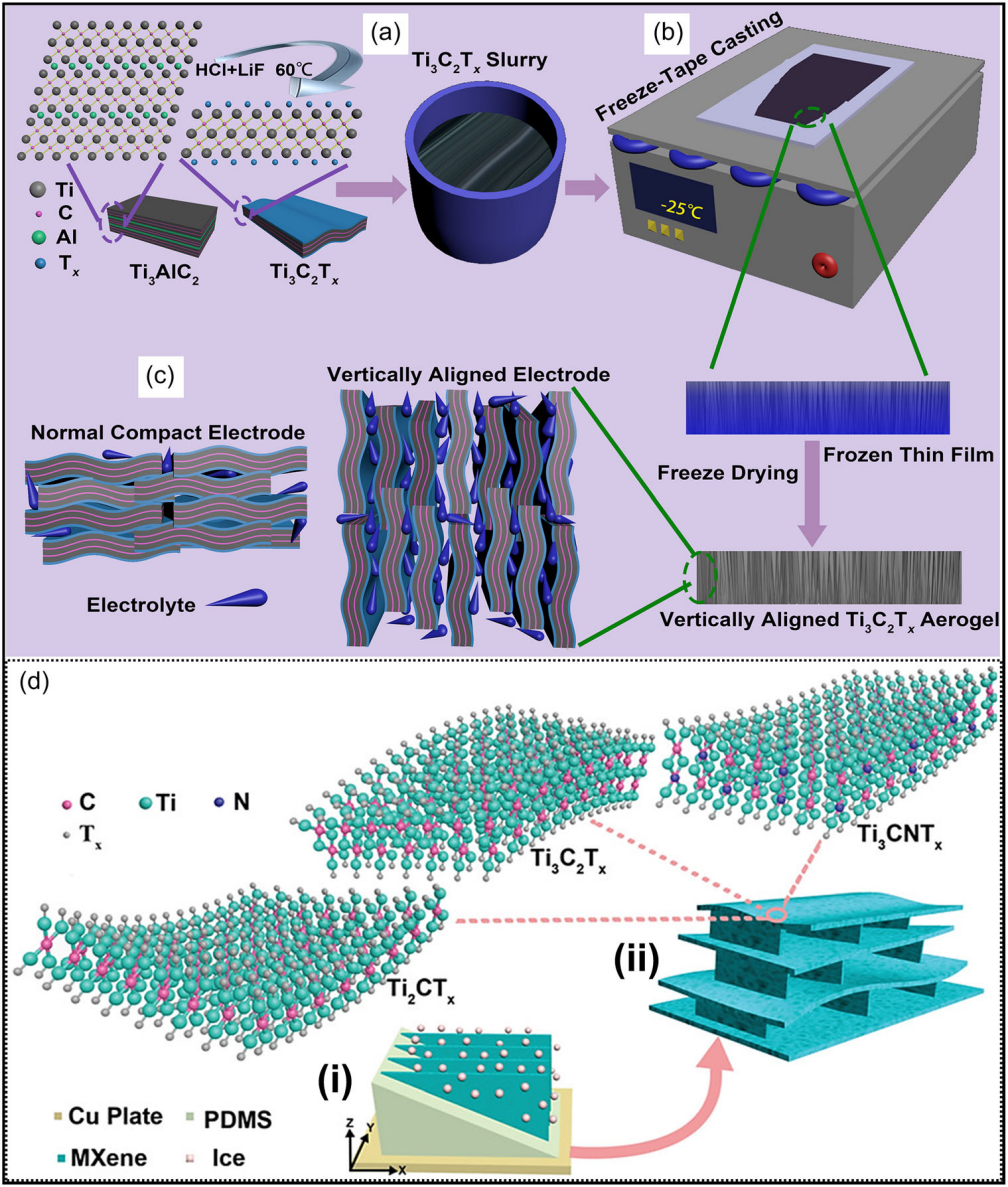
Schematic illustrations of freeze‐casting based strategies for fabricating vertically aligned MXene architectures. (a–c) Unidirectional freezing‐assisted tape casting (FaTC) process for producing vertically aligned Ti_3_C_2_T_*x*_ films: (a) preparation of few‐layer Ti_3_C_2_T_*x*_ via the MILD method at 60°C and slurry formulation; (b) formation of vertically aligned aerogel films through FaTC; and (c) electrolyte infiltration pathways within the aligned electrode structure compared with conventional dense electrodes. Adapted from [59]. Copyright 2020, Wiley‐VCH. (d) Schematic of (i) bidirectional freeze‐casting, and (ii) the resulting lamellar MXene aerogel structure with interconnected bridges, composed of Ti_2_CT_*x*_, Ti_3_C_2_T_*x*_, and Ti_3_CNT_*x*_ flakes. T_*x*_ signifies surface terminations including ^‐^OH, ^‐^O, and ^‐^F. Adapted from [60]. Copyright 2019, Wiley‐VCH.

The orientation of pores, whether ordered or randomly arranged, plays a decisive role in governing material performance. Vertically aligned MXene channels shorten ion diffusion distances and increase the accessibility of electroactive surfaces relative to disordered pore networks. Freeze‐cast MXene films with lamellar, through‐thickness channel alignment exhibit electrochemical properties that are largely insensitive to electrode thickness when applied in SCs. Notably, increasing film thickness from 150 to 300 μm results in minimal loss of high‐rate capacitance, which remains near 60% (213 F g^−1^ at 3 V s^−1^ relative to 0.02 V s^−1^). This behavior is attributed to rapid through‐plane ion transport facilitated by straight, continuous pathways formed between aligned MXene layers (Figure 4a‐c) [59]. Likewise, the alignment of MXene NSs within 3D aerogels significantly improves EMI shielding performance. Oriented architectures attenuate incident radiation more effectively than isotropic foams by enhancing multiple internal reflections and polarization losses. In a representative study, bidirectionally freeze‐cast MXene aerogels possessing long‐range ordered lamellar channels reached a SE of 70 dB at a thickness of only 1 mm (density: 11 mg cm^−3^), corresponding to a specific SE of 8818 dB.cm^3^ g^−1^. Importantly, the aligned framework enabled modulation of reflection versus absorption contributions under compression while maintaining total SE (Figure 4d) [60]. More generally, the presence of aligned pore channels gives rise to anisotropic mechanical and functional properties, which can be deliberately leveraged in applications that demand direction‐specific performance, including sensing devices and thermal regulation systems.

In EMI shielding applications, achieving an optimal balance between ultralow density and high electrical conductivity remains a fundamental challenge, as increasing porosity often disrupts conductive pathways within the MXene framework. Recent advances in microstructural engineering have demonstrated that this trade‐off can be effectively mitigated through rational architectural design. One effective strategy involves the creation of anisotropically aligned pore structures, such as lamellar or vertically oriented channels formed via freeze‐casting [59]. These architectures enable the formation of continuous, directionally aligned conductive networks that preserve efficient electron transport even at low densities. At the same time, the aligned pores promote multiple internal reflections and prolonged propagation pathways for incident EM waves, thereby enhancing absorption‐dominated shielding mechanisms.

In addition, heterogeneous interface engineering plays a critical role in improving shielding performance without significantly increasing material density. The introduction of interfaces between MXene NSs and secondary components (e.g., polymers, C materials, or magnetic NPs) generates strong interfacial polarization and dipolar relaxation losses. These mechanisms contribute to EM wave attenuation independently of bulk electrical conductivity, enabling high SE even in relatively low‐density systems [60]. Furthermore, hierarchical and hybrid architectures provide a pathway to decouple density from functionality. By integrating macroscale porous frameworks for weight reduction with nanoscale conductive networks and interfacial regions for charge transport and polarization, MXene aerogels can simultaneously achieve lightweight structures and high EMI shielding performance. These design principles highlight the importance of multiscale structural control in overcoming intrinsic material trade‐offs and advancing MXene aerogels toward practical EMI shielding applications.

**FIGURE 5:**
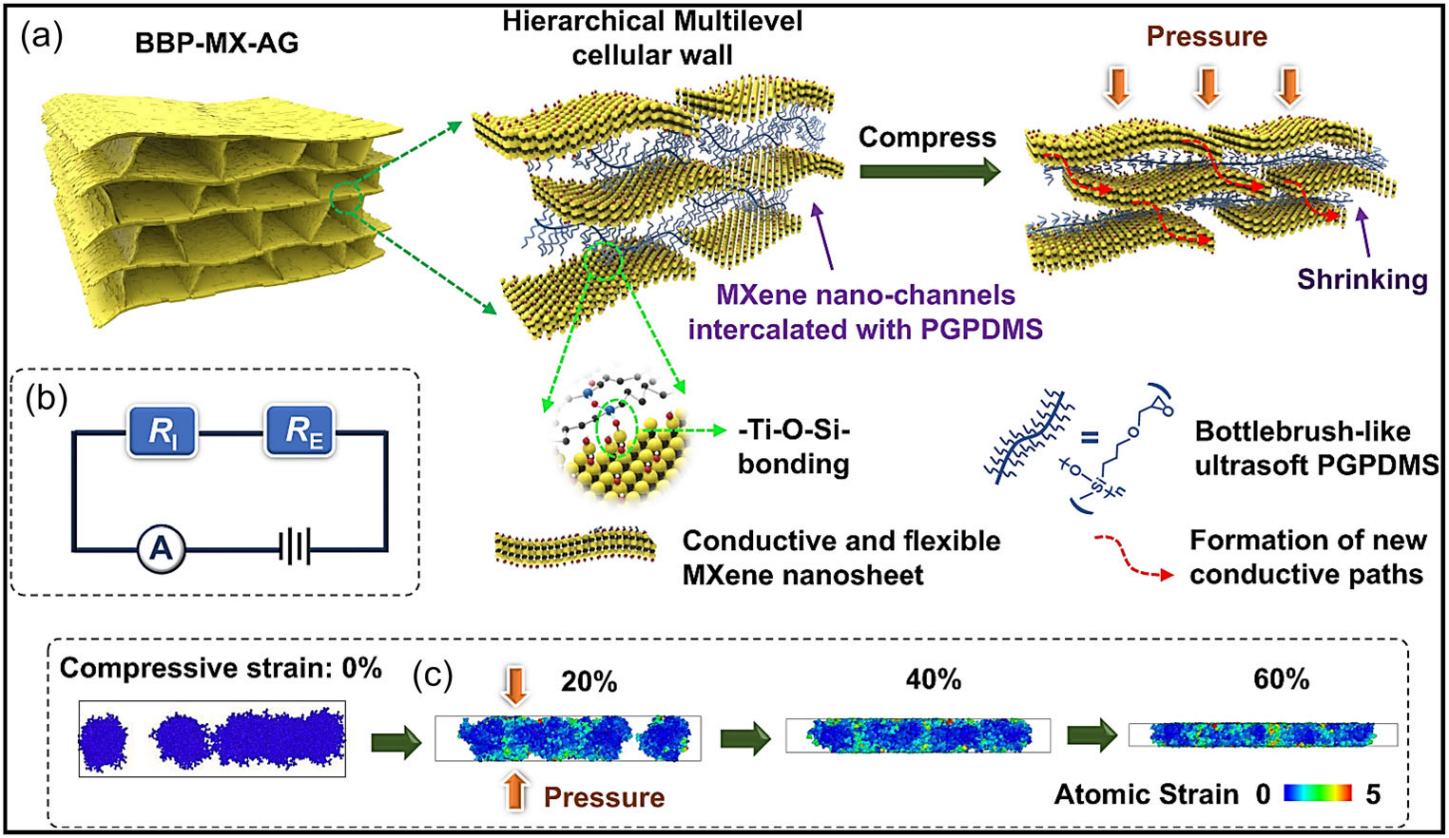
Pictorial demonstration of the BBP‐MX‐AG architecture and its deformation mechanism under applied pressure. (a) Structural illustration showing multilayer cellular walls containing bottlebrush‐type PGPDMS crosslinked MXene nanochannels and their contraction during compression. (b) Equivalent electrical circuit model of piezoresistive BBP‐MX‐AG sensor, where R_*I*_ corresponds to resistance changes arising from nanochannel contraction within cellular walls and R_*E*_ represents resistance variations caused by wall bending and buckling. (c) MD simulations depicting the configuration of PGPDMS chains confined within MXene nanochannels under compressive loading, with atoms colored according to local atomic strain. Adapted from [61]. Copyright 2022, Springer Nature.

Introducing hierarchical architectures with multiscale porosity provides an effective strategy to balance SSA, mass transport, and overall device efficiency. By integrating micro‐, meso‐, and macroporous features within a single framework, these structures maintain a large accessible SSA while preserving continuous transport channels and mechanical robustness. MXene monoliths engineered with hierarchical, layered, or bioinspired pore networks have exhibited outstanding performance across diverse applications. For instance, a recent study published in Nature reported a MXene aerogel featuring multilevel nanochannels embedded within its cellular walls, achieved through the intercalation of bottlebrush polymers between MXene NSs. This nanochannel‐rich wall architecture, combined with micron‐scale pores, resulted in an ultralow elastic modulus (∼140 Pa) and enabled highly compressible electrode behavior. Consequently, the aerogel‐based pressure sensor demonstrated the ability to resolve pressures as low as 0.0063 Pa and achieved an exceptional sensitivity exceeding 1900 kPa^−1^, substantially surpassing that of dense or single‐scale porous counterparts (Figure 5a‐c) [61].

**FIGURE 6:**
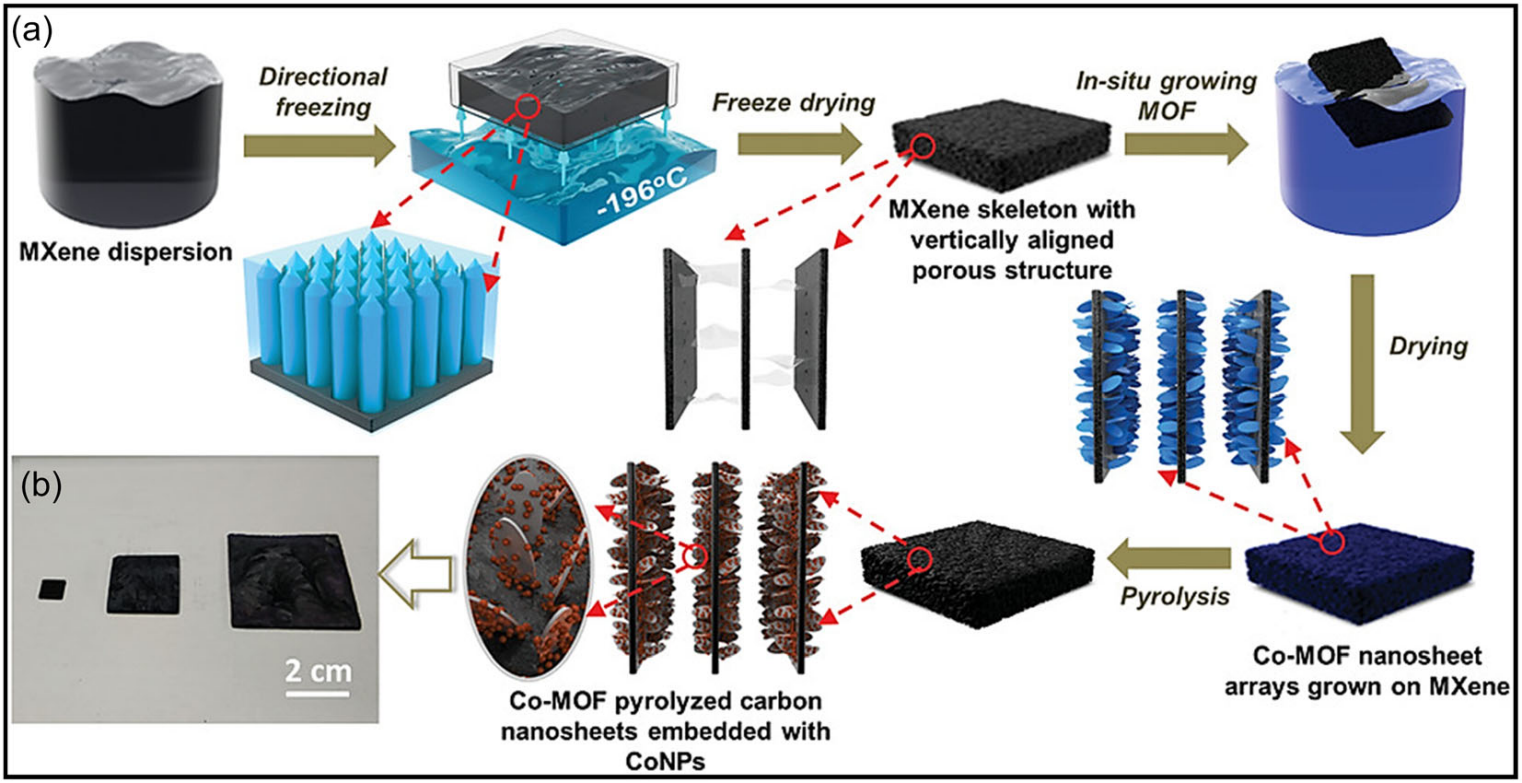
(a) Pictorial illustration of preparation route for Co‐CNS/M foam and (b) photographic images showing Co‐CNS/MXene foams fabricated in different sizes. Adapted from [62]. Copyright 2020, Wiley‐VCH.

Hierarchically porous MXene‐based architectures have also demonstrated outstanding performance in energy storage and solar‐driven desalination applications. In one example, a MXene foam coupled with MOF‐derived C nanoplates providing micro‐ and meso‐porosity, together with a macro‐porous MXene framework, achieved a solar‐vapor conversion efficiency of 93.4% under one‐sun illumination, while maintaining efficiencies above 91% over 100 h of continuous operation. This multiscale porous configuration enabled efficient light harvesting, rapid water transport, and effective salt exclusion, underscoring the advantages of hierarchical pore engineering (Figure 6a,b) [62]. Similarly, in LIB anodes, 3D MXene foams featuring interconnected pores provide abundant electroactive sites and shortened Li‐ion diffusion pathways, leading to improved capacity and rate performance. A sulfur (S)‐templated, flexible Ti_3_C_2_T_*x*_ foam electrode, for instance, delivered 455 mAh g^−1^ at 0.05 A g^−1^ and maintained ∼101 mAh g^−1^ at 18 A g^−1^, while retaining 220 mAh g^−1^ over 3500 cycles at 1 A g^−1^, far surpassing dense MXene films [63]. These findings collectively illustrate the effectiveness of combining nanoscale reactivity with micro‐ and macro‐scale transport channels in hierarchical MXene systems.

Overall, precise engineering of pore size, orientation, and hierarchical organization is critical for balancing accessible SSA, ion transport, and electrical conductivity, thereby directly determining the electrochemical and EM performance of MXene aerogels.

### 3.2 Surface Chemical Features

**FIGURE 7:**
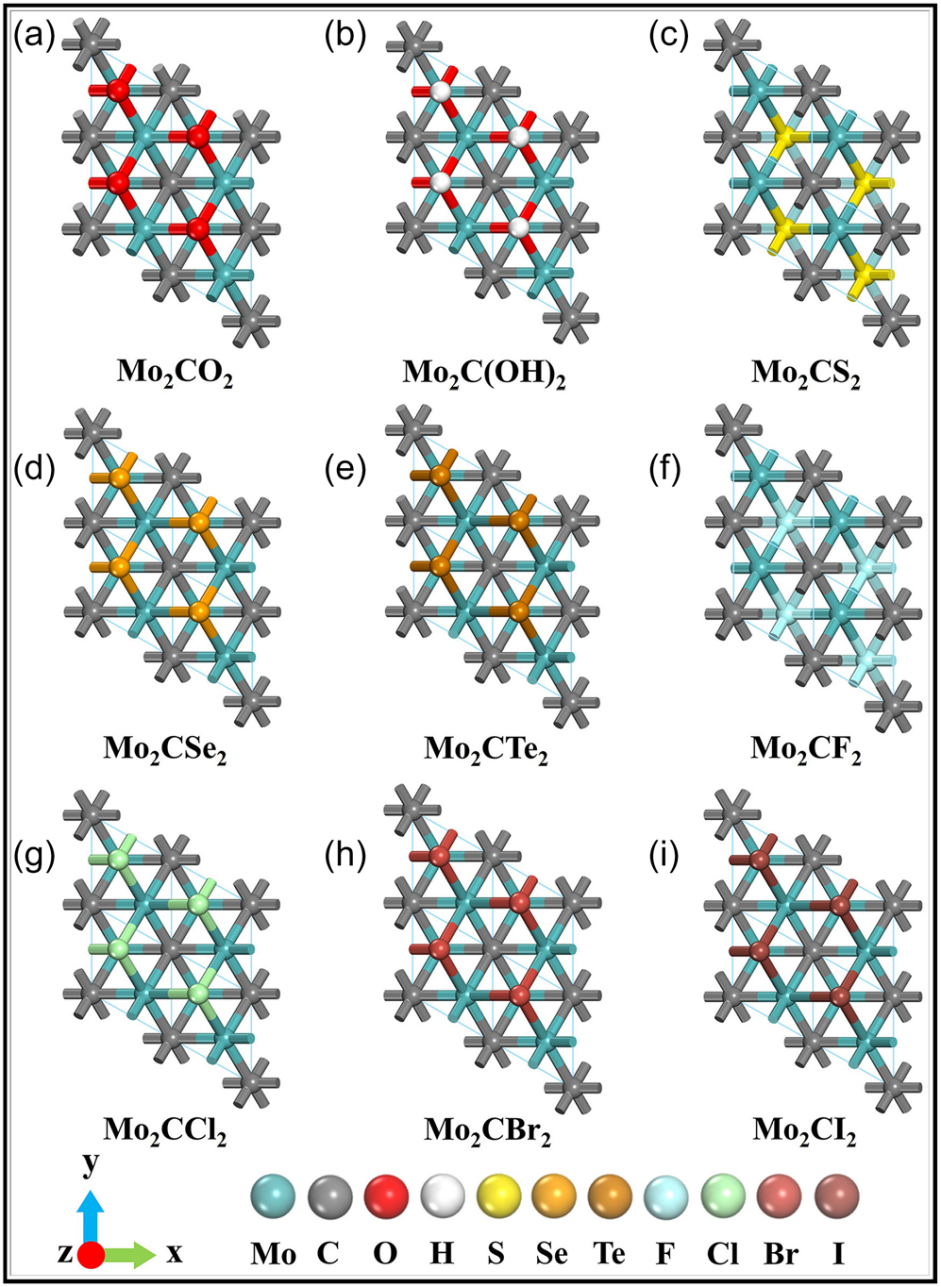
Top‐view structural models of Mo_2_CT_*x*_ MXene with different surface terminations: (a)‐O, (b) ‐OH, (c) ‐S, (d) ‐Se, (e) ‐Te, (f) ‐F, (g) ‐Cl, (h) ‐Br, and (i) ‐I. Adapted from [64]. Copyright 2024, Elsevier B.V.

MXenes typically possess surface terminations (T_*x*_ = ^‐^OH, ^‐^O, ^‐^F) that originate from their chemical etching and delamination processes (Figure 7a‐i). These functional groups play a critical role in governing interfacial interactions with polymers, NPs, and ionic species, thereby providing an effective means to tailor the properties and performance of MXene‐based composites [64].

Hydrophilic surface terminations such as ^‐^OH and ^‐^O on MXene NSs readily form hydrogen bonds with polymer chains, enabling strong interfacial interactions within polymer matrices. These interactions play a critical role in improving MXene dispersion and interfacial adhesion in MXene‐polymer hybrids. Even at low filler loadings, such interfacial compatibility can markedly enhance the mechanical performance of polymers. For example, the addition of only 0.5 wt % Ti_3_C_2_T_*x*_ to a polyurethane (PU) matrix increased the yield strength by approximately 70%, from 1.3 MPa for pristine PU to more than 2.2 MPa [65, 66]. Moreover, functional group‐driven interfacial polarization in these systems contributes to superior EMI shielding performance compared with analogous composites based on graphene fillers. MXene‐reinforced polymer composites have demonstrated EMI effectiveness far exceeding that of graphene‐based systems at comparable filler loadings. For example, shielding values as high as 92 dB have been reported for MXene‐filled polymers, compared with approximately 35 dB for graphene, which is attributed to the more diverse surface chemistry and enhanced dipolar loss mechanisms of MXenes [67]. In addition, polymer encapsulation can significantly improve the operational durability of these composites by limiting MXene oxidation [67]. High electrical conductivity has also been achieved in MXene‐polymer films; notably, a PVDF composite incorporating hybrid MXene/graphene fillers exhibited a conductivity of 13.7 S cm^−1^ while retaining low density and excellent mechanical flexibility [68]. Furthermore, the polymer matrix acts as a protective barrier against oxygen and moisture, reducing long‐term degradation. Beyond electrical applications, MXene‐polymer membranes have shown enhanced pollutant adsorption performance, where contaminants such as dyes and metal ions interact with MXene surface groups, while the polymer provides structural integrity and ease of processing.

The surface terminations of MXenes serve as effective nucleation and anchoring sites for inorganic NPs, enabling their uniform growth or deposition on MXene NSs. Interactions between metal cations or oxide clusters and surface ^‐^OH/^‐^O groups inhibit NP agglomeration in solution and instead promote heterogeneous nucleation on the MXene surface [69]. This interfacial coupling facilitates the integration of complementary functionalities. As an example, magnetic Fe‐based NPs were deposited onto MXene to construct a hybrid material with enhanced EMI shielding performance. The improved shielding originates from a dual‐loss mechanism: strong interfacial polarization induced by the polar MXene surface terminations and magnetic losses contributed by Fe_3_O_4_ NPs. As a result, the hybrid material achieves an attenuation of 86 dB at a thickness below 0.5 mm, demonstrating efficient dielectric‐magnetic synergistic shielding [70]. Likewise, the high SSA of Nb_2_C MXene has been leveraged to support the growth of Co_3_O_4_ nanocrystals, resulting in hybrid materials with improved MW absorption performance. In this system, the intrinsic electrical conductivity of the MXene complements the magnetic loss behavior of Co_3_O_4_, producing effective dielectric‐magnetic coupling for EM wave attenuation [71]. In addition to physical anchoring, MXene surface terminations can be chemically utilized to immobilize noble metal or oxide catalysts, such as Pt or Au, through Ti‐O‐M bonding, thereby generating hybrid active sites. For gas‐sensing applications, MXenes functionalized with ^‐^O groups and decorated with metallic NPs enhance surface reaction kinetics, while the conductive MXene framework efficiently transduces these reactions into electrical signals, enabling sensitive detection at room temperature. Consequently, MXenes serve as versatile 2D support platforms for dispersing NPs and achieving optimized functional synergy through controlled surface chemistry.

Surface functional groups on MXenes critically influence ion adsorption and interlayer transport behavior. In energy storage systems, for instance LIBs, ^‐^F and ^‐^OH terminations have been identified as detrimental to Li^+^ diffusion within MXene interlayers [72]. These terminations either act as physical barriers or occupy coordination sites that would otherwise accommodate Li^+^ ions. First‐principles calculations further reveal that Ti_3_C_2_ fully terminated with ^‐^F or ^‐^OH exhibits substantially lower theoretical Li storage capacities (130 and 67 mAh g^−1^, respectively) than its O‐terminated counterpart (447 mAh g^−1^) [73].

Consequently, replacing or eliminating ^‐^F/^‐^OH groups in favor of O‐based terminations markedly improves Li^+^ transport to active sites, leading to higher storage capacities and enhanced rate behavior. From a practical standpoint, both thermal and chemical modification strategies that enrich ^‐^O terminations have shown to increase the electrochemical capacity of MXene anodes while alleviating diffusion‐related kinetic constraints [72, 73, 74]. Moreover, surface termination composition plays a critical role in regulating MXene electrochemical behavior in applications beyond Li^+^ storage. For instance, ^‐^O‐terminated MXene surfaces provide a higher density of active sites for the HER owing to favorable hydrogen (H) intermediate adsorption, whereas surfaces with high ^‐^F coverage exhibit higher electrical conductivity but reduced H‐adsorption activity [75]. Notably, Tang et al. [76]. indicate that partially defluorinated MXenes display even greater HER performance. This enhancement is attributed to the exposure of abundant catalytically active ^‐^O or ^‐^OH sites following partial ^‐^F removal, while the intrinsic conductivity of the MXene is largely preserved. In aqueous environments, MXene NSs typically carry a negative surface charge and expose a high density of ^‐^OH and ^‐^O functional groups, imparting strong hydrophilicity and making them well suited for ion sieving and adsorption applications. For example, membranes based on Ti_3_C_2_T_*x*_ have demonstrated selective cation exclusion and effective heavy metal ion removal by leveraging surface charge interactions [77]. Nevertheless, O‐containing surface terminations also increase the susceptibility of MXenes to oxidation in aqueous environments, as ^‐^OH/^‐^O‐terminated surfaces readily interact with dissolved oxygen. Consequently, optimizing MXene performance requires balancing surface reactivity with chemical durability. In applications where a high density of ^‐^OH groups is essential for adsorption, strategies such as synthesis under mildly oxidizing environments or incorporation into polymer‐based protective matrices can be employed to mitigate oxidative degradation. Overall, precise control over MXene surface terminations provides a versatile strategy for tailoring interactions with polymers, NPs, or solvated ions, enabling customized design of composite materials with optimized conductivity, reactivity, and long‐term stability.

Despite the significant advantages offered by structural and surface engineering, optimizing MXene aerogels requires careful consideration of inherent performance trade‐offs, as discussed in the following subsection.

### 3.3 Performance Trade‐Offs in MXene‐Based Aerogels

The design of MXene‐based aerogels inherently involves balancing multiple interdependent properties, where the optimization of one parameter often leads to the compromise of another. Understanding and rationally managing these performance trade‐offs is therefore essential for the development of aerogels tailored to specific applications [78, 79, 80].

One of the most prominent trade‐offs arises between ultralow density and electrical conductivity. Aerogels with extremely low densities (<10 mg cm^−3^) exhibit high porosity and superior thermal insulation due to the large volume fraction of air [81, 82]. However, excessive porosity disrupts continuous conductive pathways within the MXene framework, resulting in diminished electrical conductivity and reduced EMI SE. Conversely, increasing density enhances intersheet contact and electron transport but compromises lightweight characteristics and thermal insulation performance [83, 84]. To address this limitation, hierarchical architectures that combine micro‐, meso‐, and macropores have been developed, enabling the coexistence of efficient charge transport pathways and low‐density structures. Additionally, the incorporation of conductive fillers such as CNTs [85], graphene [86], or metallic nanowires [87] helps establish percolating networks that maintain conductivity even at reduced densities.

Another critical trade‐off exists between mechanical robustness and porosity. Highly porous MXene aerogels are advantageous for mass transport and surface accessibility; however, they are mechanically fragile and prone to structural collapse under compression or cyclic loading [88]. Enhancing mechanical strength through densification or crosslinking can improve compressive modulus and fatigue resistance but may reduce pore accessibility and limit ion diffusion. Recent strategies to overcome this challenge include the introduction of polymeric binders (e.g., cellulose, polyimide, chitosan) and nanofibrous scaffolds, which provide mechanical reinforcement while preserving hierarchical porosity [89, 90]. Bioinspired architectures, such as lamellar or honeycomb‐like structures, further enable efficient stress distribution and elastic recovery without significantly sacrificing porosity [91, 92].

A further trade‐off is observed between electrical conductivity and environmental stability. Pristine MXene aerogels exhibit high electrical conductivity due to their metallic nature; however, their exposed surfaces and large SSAs make them highly susceptible to oxidation, particularly under humid or aqueous conditions [93, 94, 95]. Strategies that improve environmental stability, such as polymer encapsulation, surface passivation, or hybridization with graphene and metal oxides, often introduce insulating components or interfacial barriers that can reduce overall conductivity [96]. Optimizing this balance requires careful control over surface chemistry and interface engineering to retain conductive pathways while limiting oxidative degradation.

Trade‐offs are also evident in surface chemistry and electrochemical performance. Surface terminations (e.g., ^‐^O, ^‐^OH, ^‐^F) strongly influence ion adsorption, charge transfer, and catalytic activity [97, 98, 99]. For example, ^‐^O‐terminated MXenes favor enhanced ion storage and catalytic reactions [100], while ^‐^F terminations may improve conductivity but hinder ion transport [101]. Modifying surface terminations to optimize one function can therefore negatively impact another, necessitating targeted chemical treatments or partial functionalization strategies.

In thermal management applications, a balance must be achieved between thermal conductivity and insulation performance. While MXene frameworks can provide efficient heat conduction, particularly in dense or well‐connected networks, aerogel architectures dominated by air‐filled pores act as thermal insulators [102, 103]. Hybrid systems incorporating polymers or ceramics can tune this behavior, enabling materials that either dissipate heat effectively or minimize thermal transport depending on application requirements [104, 105].

Overall, these trade‐offs highlight that no single MXene aerogel configuration can simultaneously maximize all performance metrics. Instead, application‐specific design strategies must be employed, leveraging hierarchical structuring, compositional hybridization, and surface/interface engineering to achieve an optimal balance. Future research should focus on predictive design approaches, including computational modeling and ML, to systematically navigate these complex interdependencies and accelerate the development of next‐generation MXene aerogels with tailored multifunctional performance.

## 4 Durability and Degradation under Environmental Conditions

Among the various performance trade‐offs discussed above, environmental stability remains one of the most critical challenges. Pure MXene aerogels suffer from poor environmental stability, as they readily undergo oxidation and structural degradation under ambient environments [63]. Exposure to air, particularly in the presence of moisture, accelerates oxidation processes in MXene architectures. During this process, interlayer species such as adsorbed water are lost, and exposed reactive surface terminations (e.g., ^‐^OH groups) facilitate subsequent oxidation of the MXene lattice, ultimately leading to the transformation of Ti‐C bonds into TiO_2_ [106]. In situ spectroscopic analyses reveal that degradation proceeds through multiple stages, including depletion of intercalated ethanol, an increase in C–C bonding within the C framework, and the formation of TiO_2_ phases, initially anatase and later rutile, as final decomposition products. Exposure to high humidity or direct contact with liquid water significantly accelerates MXene degradation pathways. Under aqueous conditions, water initiates hydrolysis reactions that become the dominant mechanism of MXene oxidation [107]. This hydrolysis‐driven oxidation proceeds particularly rapidly in neutral and alkaline environments, leading to pronounced losses in both conductivity and mechanical integrity of MXene aerogels. Elevated temperatures further accelerate oxidation kinetics. Although densely stacked MXene NSs provide partial intrinsic protection, acting as diffusion barriers to oxygen and offering limited surface passivation through terminations, these effects are insufficient under prolonged thermal or oxidative stress [4]. Over extended exposure, unprotected MXene aerogels undergo structural degradation, including NSs oxidation and restacking, which disrupts the porous 3D architecture. This degradation manifests as pore collapse, reduced SSA, and eventual disintegration of the aerogel architecture, ultimately compromising material behavior.

To mitigate environmental degradation, composite MXene aerogels incorporating environmentally benign biopolymers have been developed. The inclusion of polysaccharides such as cellulose and agar, as well as natural polymers like chitosan, markedly improves both structural stability and sustainability. Within these composites, MXene NSs are interconnected with the biopolymer matrix through H‐bonding and electrostatic interactions, forming a protective network that limits direct contact between MXenes and the surrounding environment [108, 109, 110]. For example, MXene NSs anchored within chitosan‐cellulose fiber scaffolds are effectively protected against oxidative degradation, as the H‐bonded polymer network serves as a molecular barrier that restricts the penetration of oxygen and moisture [108]. This conformal biopolymer coating substantially suppresses MXene oxidation and hydrolytic processes, thereby prolonging the operational lifetime of aerogel relative to uncoated MXene assemblies. Moreover, the interconnected biopolymer framework improves mechanical stability by inhibiting NSs restacking and maintaining the structural integrity of the 3D architecture under applied stress. In addition to performance benefits, these composite systems provide environmental advantages, since cellulose and chitosan are renewable, biodegradable, and nontoxic biopolymers [111]. Recent investigations have shown that MXene‐cellulose composite aerogels are capable of complete biodegradation following their service life, thereby substantially reducing their environmental footprint [112]. As a result, MXene aerogels incorporating biopolymer components not only demonstrate enhanced resistance to environmental aging but also support sustainability objectives by decreasing dependence on petroleum‐based materials. Continued research efforts are focused on advancing these systems to facilitate the environmentally responsible application of MXene aerogels. Overall, while MXene aerogels exhibit exceptional functional properties, their susceptibility to oxidation and structural degradation remains a critical limitation, necessitating protective strategies such as surface modification, hybridization, and encapsulation for long‐term stability.

## 5 Comparative Evaluation of Pristine and Composite MXene Aerogels

Pristine MXene aerogels are constructed solely from MXene NSs, either as a single MXene phase or as mixed MXene compositions. In contrast, hybrid MXene aerogels incorporate additional components, such as metal oxides, graphene, or polymeric materials, within the MXene framework [113]. The choice between pristine and hybrid systems depends largely on targeted material properties: while pristine MXene aerogels maximize MXene content and offer relatively straightforward fabrication, hybrid architectures enable tunable property optimization to achieve more dependable performance. Growing evidence in the literature indicates that rationally designed hybrids can impart enhanced mechanical robustness, elasticity, and structural resilience without sacrificing key MXene attributes, including high SSA and electrical conductivity [1, 114, 115]. This comparative understanding provides valuable guidance for the development of next‐generation MXene aerogels with improved stability and multifunctionality for advanced material applications.

### 5.1 Mechanical Characteristics

Pristine MXene aerogels generally exhibit limited mechanical strength and elasticity. Although MXene NSs possess abundant surface functional groups (e.g., ^‐^OH) that can promote H‐bonding, single‐component MXene frameworks typically display weak interlayer interactions and insufficient gelation, resulting in fragile structural frameworks. Compression testing commonly shows that pristine MXene aerogels lack effective elastic recovery [114]. By contrast, the introduction of secondary components markedly improves mechanical integrity. In hybrid MXene aerogels, polymers, nanofibrous (NFs) materials, or other 2D additives act as binders or supporting scaffolds, forming strong interactions with MXene NSs and reinforcing the overall structure. For example, hydrophilic polymers like PVA and cellulose NFs can establish extensive H–bonding interactions with MXene NSs, thereby suppressing NS aggregation and promoting effective stress transfer within the network [116]. As a result, composite MXene aerogels exhibit enhanced tensile strength, compressibility, and elastic recovery compared with both pristine MXene aerogels and polymer‐only aerogels. Notably, a reported MXene/cellulose aerogel maintained structural integrity under 50% compressive strain over 5000 loading‐unloading cycles, demonstrating excellent fatigue resistance [117]. These findings indicate that intrinsically brittle MXene frameworks can be transformed into mechanically robust and highly elastic structures through appropriate hybridization.

### 5.2 Tunable Characteristics Governed by Structural Design

**FIGURE 8:**
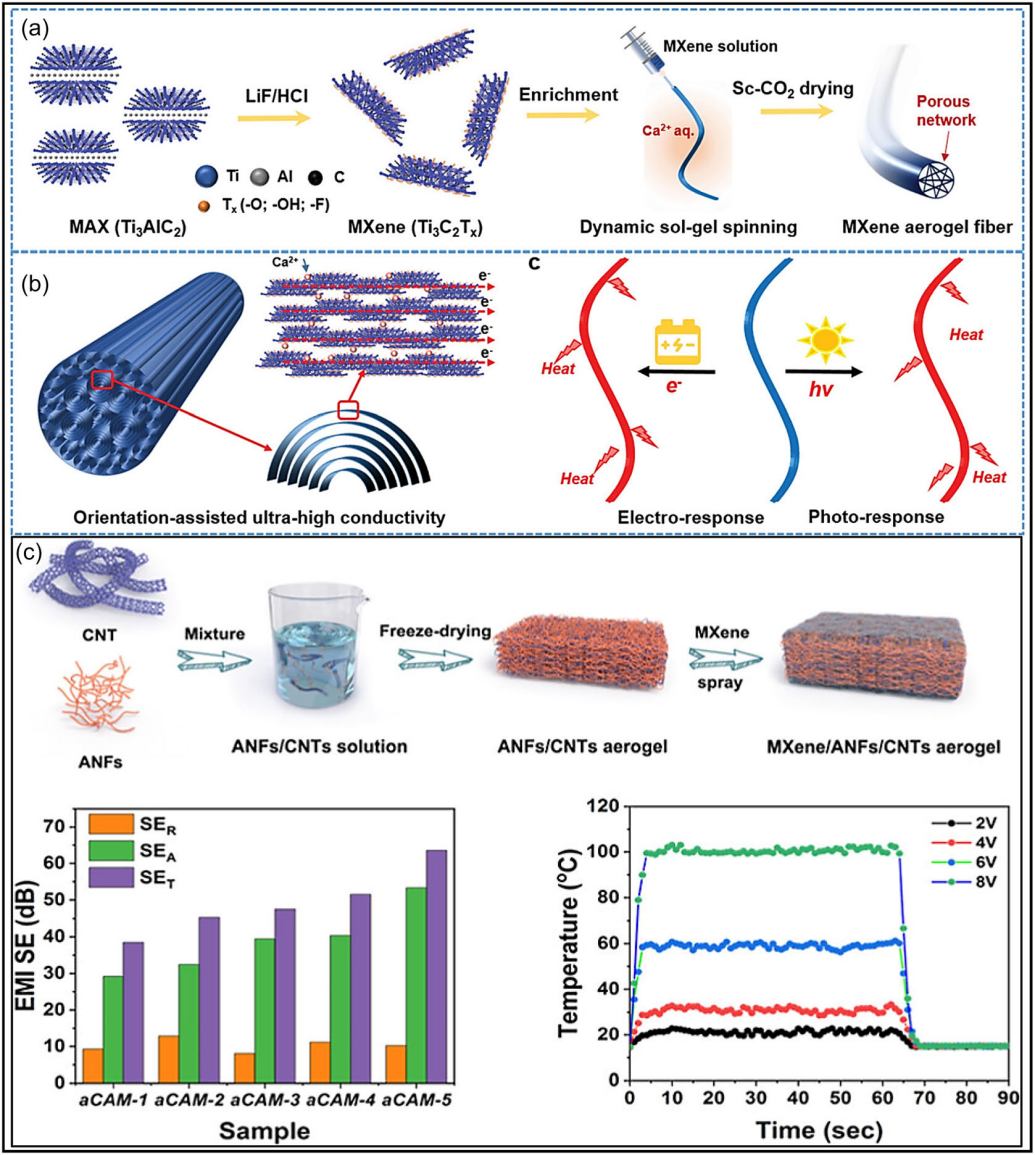
(a,b) Highly oriented Ti_3_C_2_T_*x*_ aerogel NFs exhibiting conductivity and optical responsiveness. Adapted from [118]. Copyright 2022, Wiley‐VCH. (c) MXene/CNT/aramid composite aerogels designed for EMI shielding and Joule heating applications. Adapted from [119]. Copyright 2023, American chemical society.

Pristine MXene aerogels can exhibit extremely high porosity (>95%) along with very low bulk density. Using sol–gel routes combined with controlled drying techniques, such as freeze casting or supercritical drying, all‐MXene aerogels with adjustable pore architectures and SSAs up to 142 m^2^ g^−1^ have been successfully fabricated. For example, oriented Ti_3_C_2_T_*x*_ aerogel NFs maintain a continuous 3D framework, achieving porosities of approximately 99% and ultralow densities of about 0.035 g cm^−3^ (Figure 8a,b) [118]. However, MXene‐only frameworks are prone to structural collapse or severe sheet restacking in the absence of secondary supports, leading to reduced interlayer spacing, loss of accessible porosity, and compromised mechanical durability [120]. Hybrid MXene aerogels offer significantly greater structural tunability than their pristine counterparts. By incorporating 1D or 2D fillers, such as CNFs or GO, the pore size, orientation, and overall architecture can be precisely engineered. These additives function as supporting scaffolds that suppress MXene NSs restacking and promote the formation of hierarchical pore structures. Strong interfacial interactions, including H‐bonding and van der Waals forces, between MXene NSs and the fillers contribute to the development of stable, interconnected networks. For instance, the introduction of CNFs into MXene gels has been shown to increase interlayer spacing and generate biomimetic lamellar architectures with thinner pore walls and higher SSAs than pristine MXene aerogels (Figure 8c) [119]. Likewise, MXene‐coated GO assemblies exhibit layered structures with improved compressive modulus and structural stability [121]. In summary, hybridization enables precise control over pore architecture and interlayer spacing, overcoming the structural limitations of pristine MXene aerogels and enhancing functional performance.

### 5.3 Thermal Robustness and Conductive Behavior

**FIGURE 9:**
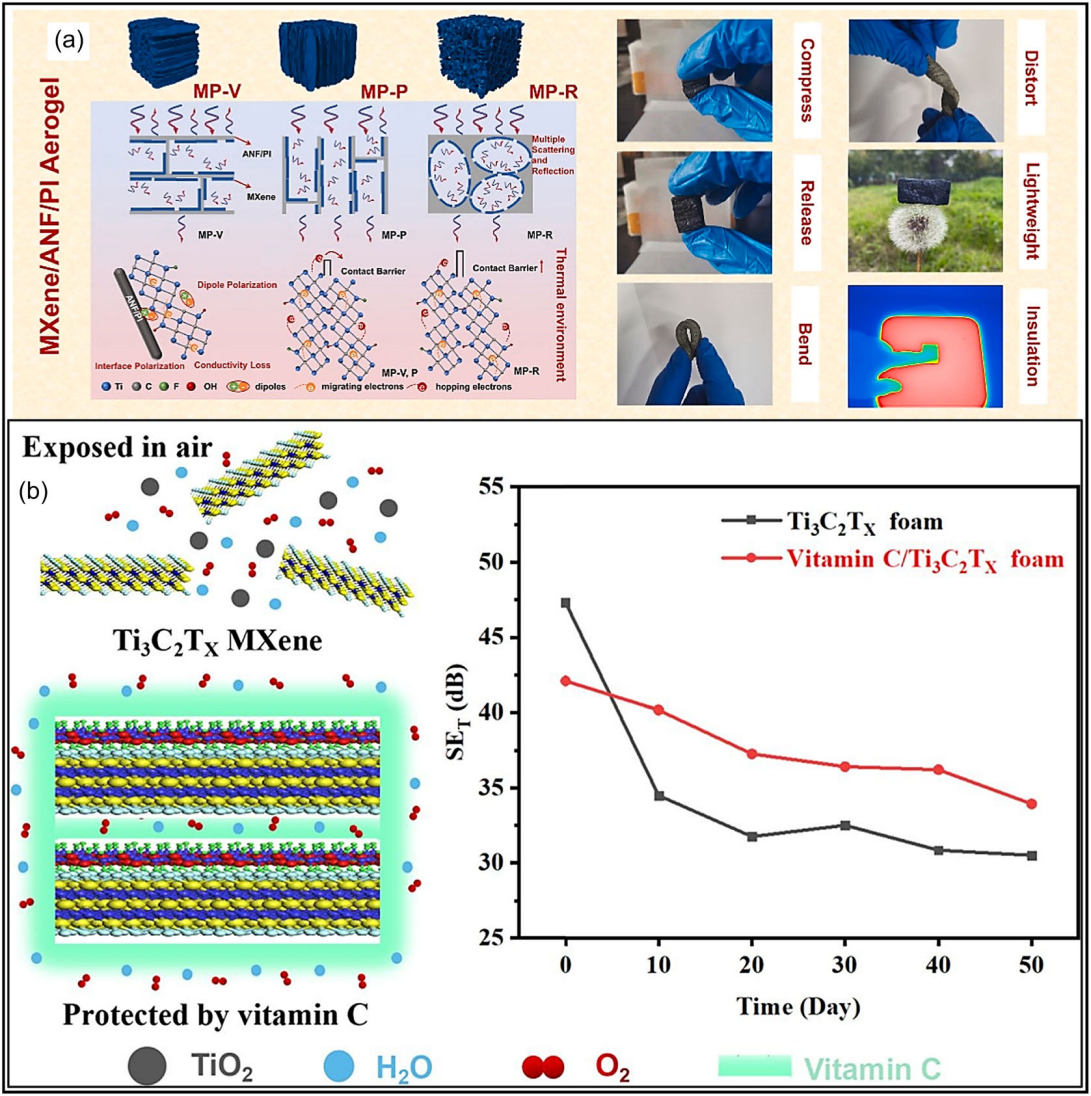
(a) Ti_3_C_2_T_*x*_/aramid NF/PI composite aerogels exhibiting effective thermal insulation and tunable EM wave absorption under thermal conditions. Adapted from [128]. Copyright 2022, Elsevier B.V. (b) Vitamin C‐modified MXene aerogel with enhanced oxidation resistance. Adapted from [129]. Copyright 2023, Elsevier B.V.

A primary drawback of pristine MXene aerogels is their limited resistance to thermal oxidation. MXene structures, particularly Ti_3_C_2_T_*x*_, are highly sensitive to oxygen; when configured as porous aerogels with great SSAs, they readily undergo oxidation upon exposure to air or moisture, resulting in a loss of electrical conductivity and mechanical integrity [122]. Even in the absence of severe conditions, pristine MXene structures can gradually transform into metal oxides at moderate temperatures, thereby compromising long‐term stability. In contrast, hybrid MXene aerogels can be engineered to exhibit enhanced thermal tolerance. The incorporation of thermally stable components, such as ceramic NFs, C‐based architectures, or high‐temperature polymers, confers improved resistance to heat and flame. For example, an MXene/CNF aerogel coated with a flame‐retardant poly(phosphate)/PU layer remained structurally intact over a wide temperature range from −196 to 300°C [123]. Such composite strategies also enhance fire safety and suppress thermal degradation by enabling char formation or thermal insulation, while protective coatings limit oxygen and moisture penetration during heating [124]. Pristine MXene aerogels are expected to exhibit higher intrinsic thermal conductivity than polymer‐based aerogels due to the metallic nature of MXene NSs. When assembled into a continuous MXene framework, these aerogels provide efficient pathways for heat transport, analogous to their high conductivity. Indeed, previous studies have reported that all‐MXene aerogels combine relatively high thermal conductivity with ultralow density [125, 126]. However, despite this intrinsic behavior, the overall heat transport in such highly porous materials remains limited in practice, as heat transfer is largely governed by the stagnant air trapped within the pore network. Consequently, pristine MXene aerogels function as poor thermal insulators, similar to other aerogel systems. Thermal conductivity in MXene aerogels can be effectively modulated through hybridization. Incorporation of thermally insulating phases, such as polymers or metal oxides, reduces overall heat transport, thereby enhancing thermal insulation performance [127]. For example, an MXene/aramid NF/polyimide (PI) composite aerogel demonstrated excellent thermal insulating behavior, as the PI matrix disrupted heat conduction pathways despite the presence of thermally conductive fillers (Figure 9a) [128]. Conversely, thermal conductivity can be significantly increased by introducing highly conductive additives, including CNTs, GO, or Ag nanowires (NWs), provided that a continuous percolating framework is established [124, 128, 130]. In summary, pristine MXene aerogels typically exhibit high thermal conductivity suitable for heat dissipation, whereas hybrid MXene aerogels offer tunable thermal behavior, spanning from ultralow conductivity for insulation to enhanced conductivity for thermal regulation, depending on hybrid composition.

### 5.4 Resistance to Oxidation

Pristine MXene aerogels are well known to be highly prone to oxidative degradation when exposed to air, particularly under humid conditions. Their low packing density and large SSA accelerate oxidation processes, such as the formation of TiO_2_, which progressively deteriorate both electrical conductivity and mechanical integrity, ultimately leading to structural degradation over time (Figure 9b) [129]. Consequently, ambient stability remains a critical challenge for MXene aerogels, as unprotected MXene frameworks experience substantial performance losses under oxidative environments. In contrast, hybrid MXene aerogels exhibit significantly improved stability. One effective strategy involves physical shielding, whereby secondary components encapsulate or surround MXene NSs, thereby limiting their exposure to oxygen and moisture. For example, in MXene/graphene hybrid aerogels, graphene layers act as protective barriers around MXene NSs, effectively suppressing oxidation [121]. In addition, composite MXene aerogels are often protected by polymer coatings that function as effective barriers against moisture and oxygen. It has been demonstrated that encapsulating MXene/CNF networks with a thin PU/phosphate layer generates a roughened surface that suppresses moisture penetration and significantly inhibits MXene oxidation [121, 131]. Beyond physical shielding, chemical stabilization also plays a critical role in improving oxidation resistance. Certain additives inherently enhance stability by scavenging oxygen or slowing degradation reactions when incorporated into MXene‐based hybrid structures [122]. Furthermore, the strong H–bonding interactions in MXene‐cellulose aerogels not only impart great mechanical strength but also increase surface hydrophobicity, thereby improving resistance to oxidative degradation and extending aerogel lifetime relative to pristine MXene systems [122]. In general, composite MXene aerogels exhibit substantially improved resistance to oxidation and environmental degradation. As a result, they maintain electrical and mechanical performance over extended periods, whereas pristine MXene aerogels are highly sensitive and require careful handling to prevent rapid property deterioration. The incorporation of MXene NSs into protective matrices or surface coatings has enabled the development of aerogels that are stable in ambient and humid atmospheres, which is critical for practical applications.

## 6 Characterization Tools

**FIGURE 10:**
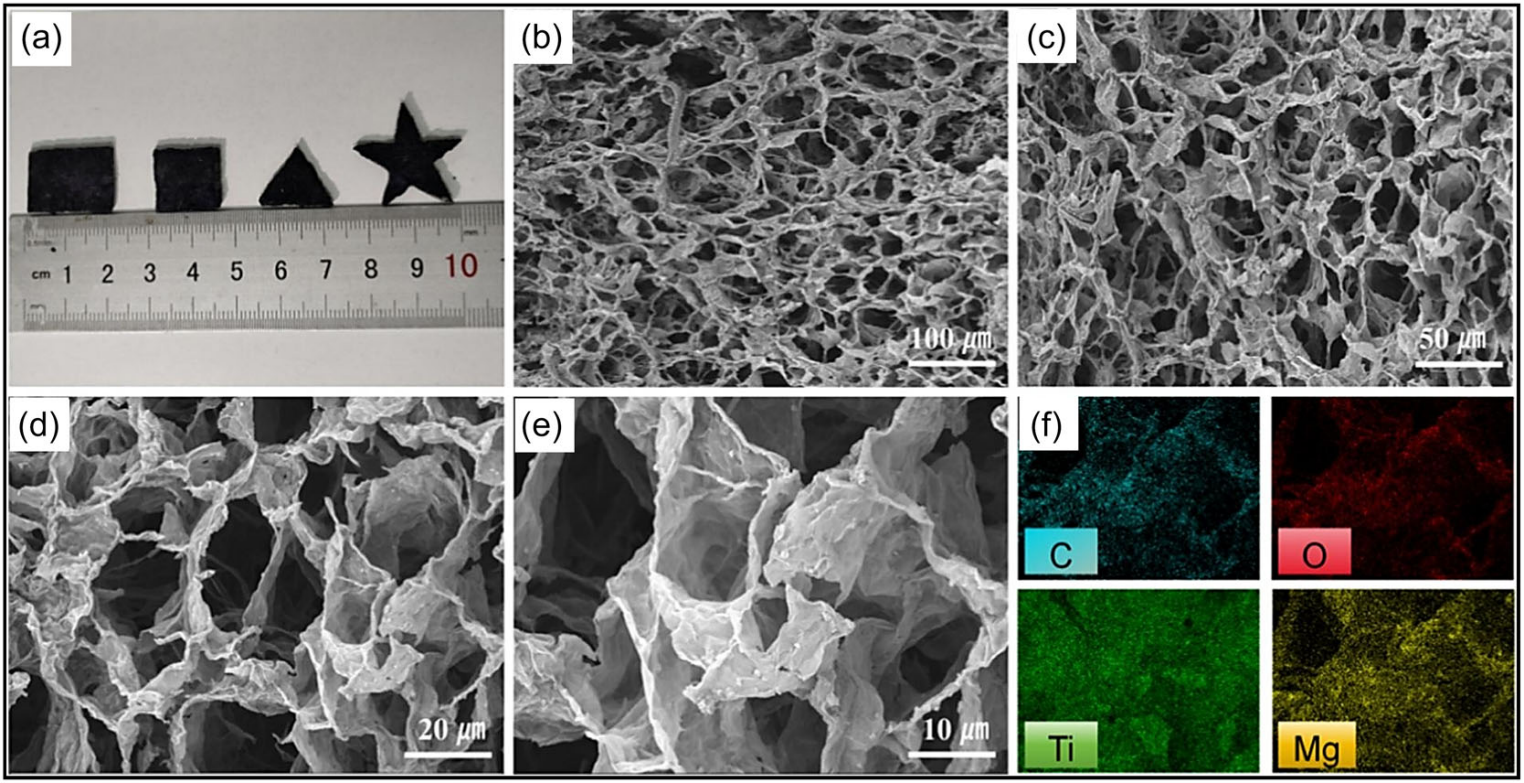
(a) Mg‐crosslinked cellulose/Ti_3_C_2_T_*x*_‐melamine sponge aerogels fabricated in different macroscopic geometries. (b–e) SEM images illustrating the porous architecture and microstructural features, and (f) elemental mapping showing the spatial distribution of constituent elements. Adapted from [132]. Copyright 2024, Elsevier B.V.

Comprehensive structural and compositional characterization is essential for understanding MXene aerogels (Figure 10a), and a range of analytical tools is routinely applied to examine their morphology, internal structure, surface chemistry, and functional characteristics [133]. SEM is used to visualize the hierarchical porosity and lamellar frameworks at the microscale, whereas transmission electron microscopy (TEM) offers nanoscale resolution of NSs stacking, lattice fringes, and local crystallinity within the 3D network [134]. X‐ray photoelectron spectroscopy (XPS) provides detailed information on surface composition and chemical bonding, enabling identification of MXene constituents (e.g., C, Ti, O, and F) and monitoring of oxidation states or dopant incorporation during aerogel synthesis [135]. In parallel, DFT measurements support experimental findings by elucidating atomic‐level interactions and predicting structure‐property relationships, such as charge–transfer‐driven binding between MXene surface terminations and polymer chains, thereby rationalizing the observed electronic and mechanical behaviors [136].

SEM is a key technique for examining MXene aerogels, as it provides direct visualization of 3D framework, pore architecture, and interconnection quality that strongly influence mass transport and mechanical behavior. As shown in Figure 10b‐d, the aerogel exhibits a well‐preserved open‐cell structure in which Ti_3_C_2_T_*x*_ NSs and microfibrillated cellulose uniformly coat the melamine scaffold, forming continuous struts and interconnected junctions rather than collapsed pore walls. The pore network displays a hierarchical distribution, with large macropores facilitating electrolyte penetration and smaller mesoporous channels reducing transport resistance and enabling continuous electron and ion pathways. High‐magnification graphs (Figure 10e) further reveal corrugated MXene lamellae decorating the pore surfaces; such nanoscale roughness increases the effective SSA and introduces additional edge or defect sites for charge storage or interfacial interactions. Elemental mapping (Figure 10f) confirms homogeneous Mg^2+^ distribution across the aerogel, indicating effective ionic crosslinking that suppresses MXene restacking, maintains interlayer spacing, and stabilizes the porous framework during compression and cyclic wetting/drying processes [132].

**FIGURE 11:**
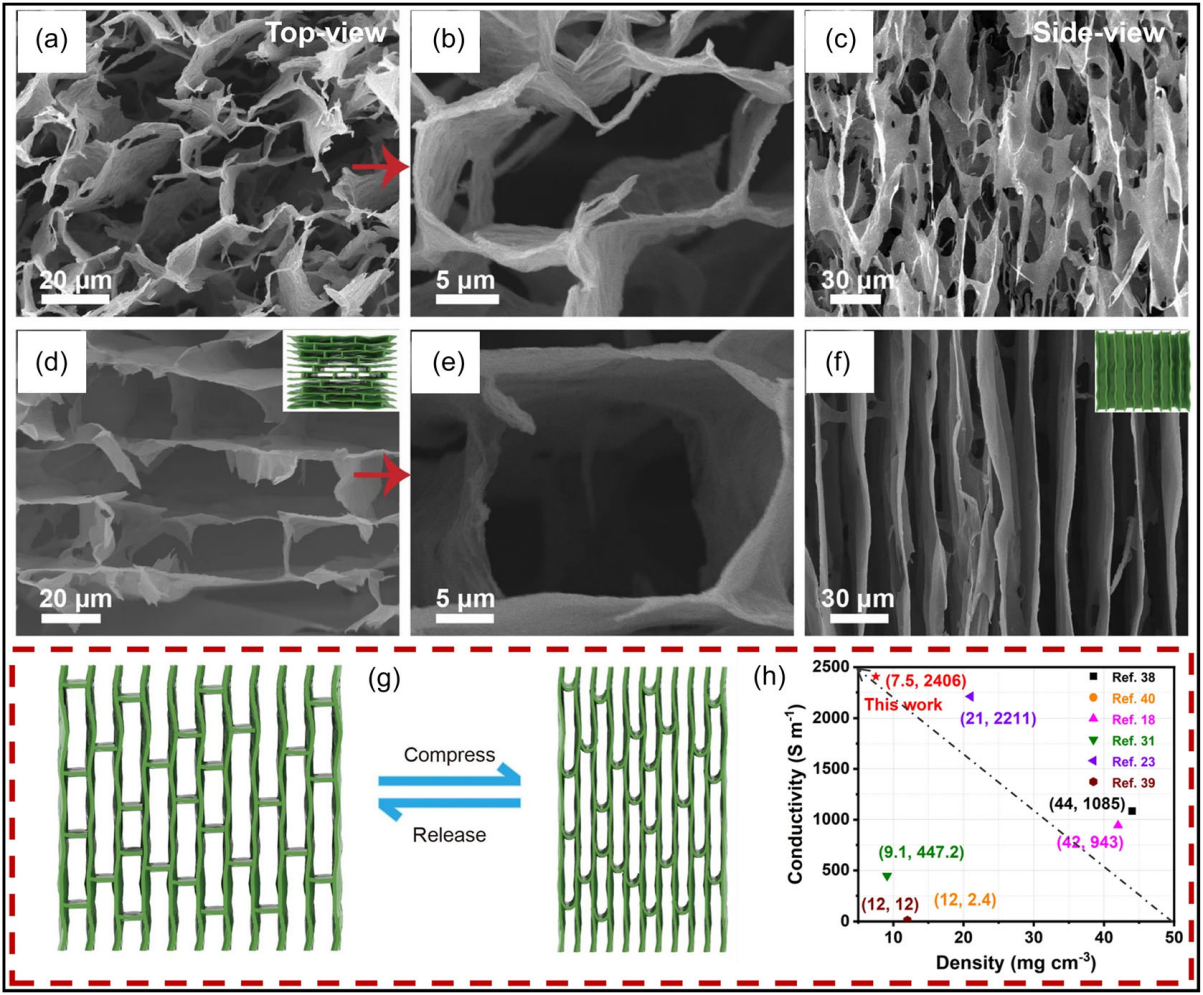
(a–c) SEM micrographs (top and side views) of CNT/MXene aerogel. (d–f) Corresponding SEM micrographs of CNF/CNT/MXene aerogels with an inset showing the pore‐structure schematic. (g,h) Illustration of compressive deformation and recovery and conductivity comparison among MXene‐related aerogels. Adapted from [123]. Copyright 2023, Springer Nature.

SEM analysis of the CNT/MXene aerogel reveals a loosely arranged and irregular pore structure (Figure 11a‐c), characterized by weak intersheet contacts that compromise mechanical integrity and promote fracture under high compressive strain. In contrast, the introduction of cellulose NFs (CNFs) results in CNF/CNT/MXene aerogels with a highly organized, tracheid‐like lattice structure featuring smooth pore surfaces and strong interfacial connectivity (Figure 11d‐f). This structural evolution is primarily driven by extensive H–bonding interactions, which function as a binding medium to integrate CNTs and MXene NSs into a cohesive network. The mechanical model presented in Figure 11g illustrates effective stress propagation throughout the interconnected framework, explaining the rapid elastic recovery and exceptional resilience observed during cyclic compression. Remarkably, despite an ultralow density of 7.5 mg cm^−3^, aerogel exhibits a high bulk electrical conductivity of 2400S m^−1^ with low anisotropy (vertical: 2080S m^−1^; longitudinal: 2300S m^−1^). As illustrated in Figure 11h, this combination of properties places the material among the highest‐performing MXene‐based aerogels, highlighting the importance of CNF‐mediated junction stability and alignment in achieving both mechanical robustness and metallic‐level electrical transport [123].

**FIGURE 12:**
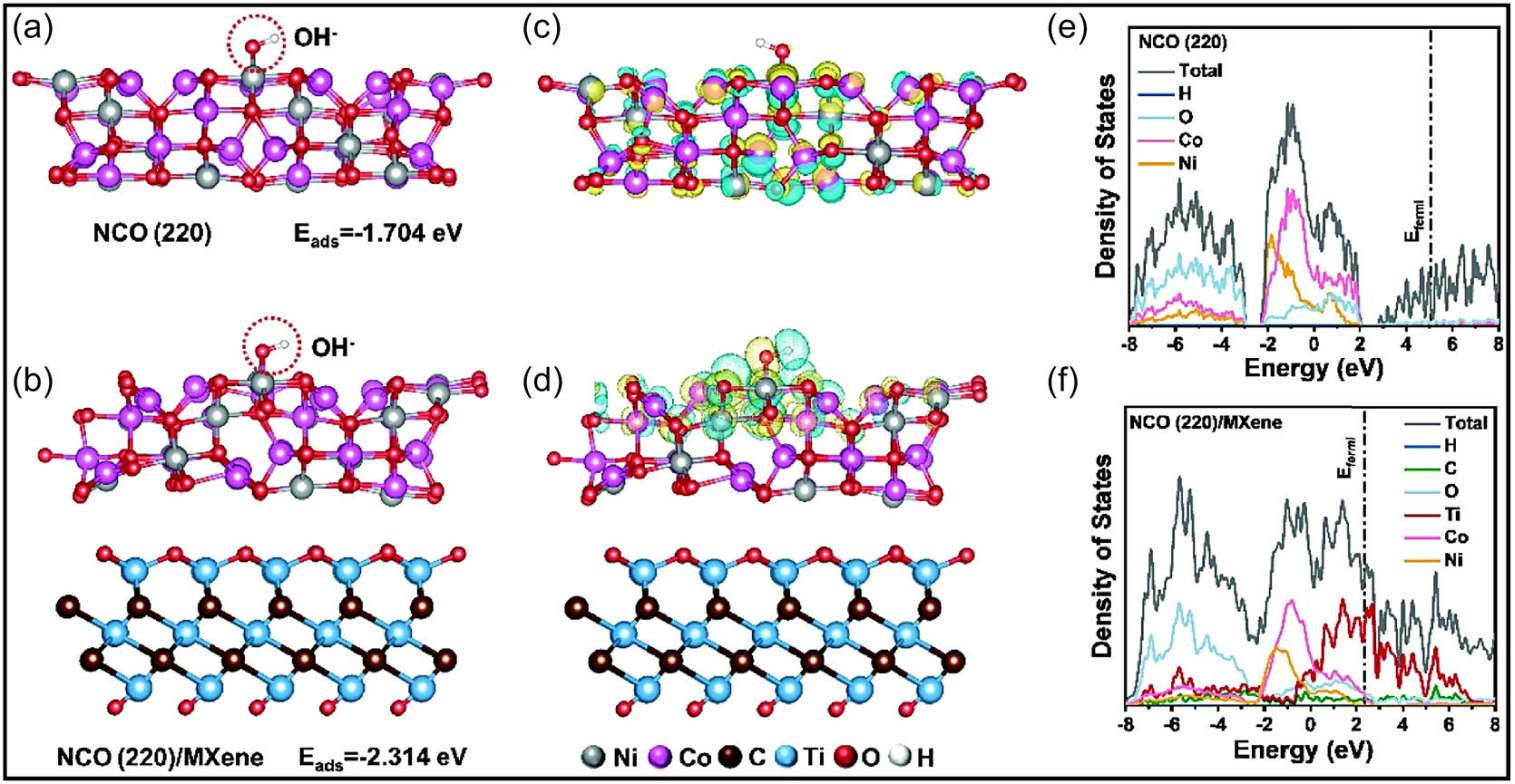
Calculated OH^‐^ adsorption energies on (a) NCO (220) and (b) the NCO (220)/MXene heterostructure. (c,d) Charge‐density difference maps for NCO (220) and NCO (220)/MXene, where cyan and yellow regions denote electron depletion and accumulation, respectively. (e,f) DOS profiles of NCO (220) and the NCO (220)/MXene interface; dashed lines mark the Fermi level. Adapted from [136]. Copyright 2023, Elsevier B.V.

To elucidate the origin of enhanced electrochemical performance of NiCo_2_O_4_/Ti_3_C_2_T_*x*_‐rGO aerogel, DFT calculations were carried out on an easy NCO (220)/MXene heterointerface that represents the MGA network. The results reveal substantially stronger OH^‐^ adsorption at the heterojunction, with an adsorption energy of −2.314 eV, compared with −1.704 eV for pristine NCO (220), indicating more effective reactant adsorption and faster interfacial redox kinetics (Figure 12a,b). Charge‐density difference plots (Figure 12c,d) show pronounced electron accumulation on NCO side, confirming significant interfacial charge transfer. Furthermore, DOS analysis (Figure 12e,f) indicates that while bare NCO (220) exhibits a low electronic density near the Fermi level, the NCO (220)/MXene heterostructure displays a markedly increased DOS in this region. This enhancement reflects strong electronic coupling between NCO and the MXene support, leading to improved electrical conductivity. Taken together, the strengthened OH^‐^ chemisorption and interfacial electronic interaction effectively reduce kinetic barriers and enhance electron/ion transport, providing a mechanistic explanation for high capacitance and rate capability of mechanically resilient hybrid aerogel [136].

**FIGURE 13:**
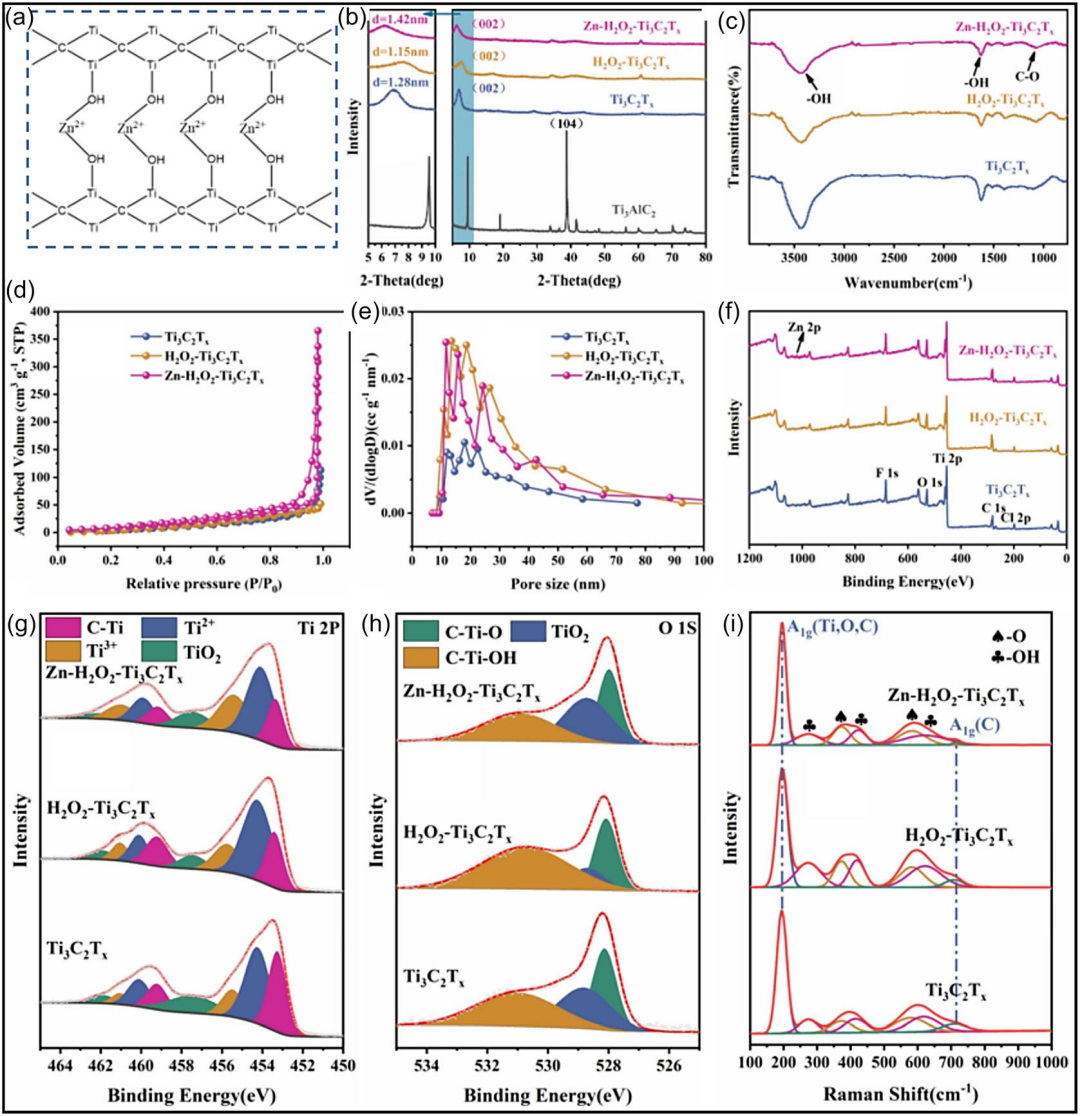
(a) Illustration of Zn^2+^ coordination with MXene. Comparative characterization of Ti_3_C_2_T_*x*_, H_2_O_2_‐Ti_3_C_2_T_*x*_, and Zn‐H_2_O_2_‐Ti_3_C_2_T_*x*_ by (b) XRD, (c) FTIR, (d) N_2_ adsorption–desorption analysis, and (e) BJH pore size distribution. (f) XPS survey spectra with corresponding (g) Ti 2p and (h) O 1s spectra. (i) Raman spectra of the three MXene samples. Adapted from [137]. Copyright 2024, Elsevier B.V.

In a separate investigation, a MXene‐based composite aerogel was systematically examined using a suite of characterization tools, which uncovered strong interactions between MXene surface terminations and Zn^2+^ species released during the removal of a Zn powder sacrificial template (Figure 13a) [137]. These interactions induce changes in the interlayer distance of MXene NSs and facilitate cross‐linking with surface hydroxyl groups, thereby constructing a 3D macroporous framework. XRD analysis (Figure 13b) reveals a shift in the Ti_3_C_2_T_*x*_ (002) diffraction peak following etching, confirming successful MXene formation. The subsequent introduction of Zn^2+^ leads to an additional shift of the (002) peak toward lower diffraction angles, consistent with an increased interlayer distance. fourier transform infrared (FTIR) and Raman analyses (Figure 13c,i) demonstrate significant alterations in surface chemistry of the MXene aerogel. Specifically, FTIR spectra reveal weakened hydroxyl stretching and bending modes in Zn‐H_2_O_2_‐Ti_3_C_2_T_*x*_, consistent with coordination between surface ^‐^OH groups and Zn^2+^ ions. N_2_ adsorption–desorption isotherms (Figure 13d) indicate that Zn‐H_2_O_2_‐Ti_3_C_2_T_*x*_ exhibits a higher SSA compared with pristine Ti_3_C_2_T_*x*_, which is advantageous for electrochemical applications. BJH pore size distribution analysis (Figure 13e) confirms the presence of a hierarchical pore system comprising micro‐, meso‐, and macro‐pores. XPS results (Figure 13f) verify the successful introduction of Zn^2+^ species, evidenced by the emergence of Zn 2p peaks. Additionally, binding energy shifts in the Ti 2p and O 1s regions (Figure 13g,h) indicate strong chemical interactions between Zn^2+^ ions and MXene surface functional groups. Raman spectra (Figure 13i) show an upshift of the A_1_g vibration, suggesting inhibited NSs restacking and the formation of a well‐intercalated, structurally stable MXene aerogel. In summary, advanced characterization techniques provide critical insights into the structure‐property relationships of MXene aerogels, enabling rational design and performance optimization at multiple length scales.

## 7 Applications of MXene‐Based Aerogels across Multiple Disciplines

Over the past decade, MXene‐based aerogels have overcome traditional disciplinary boundaries and emerged as versatile platform materials for multifunctional applications spanning energy, environmental remediation, healthcare, and advanced technologies [138]. Initial research focused primarily on energy storage, where these aerogels enabled the development of SCs with long cycle life and high charge storage, as well as improved battery systems (Li‐, Na‐, K‐ion, and Li‐S) owing to its great conductivity and tunable porous architectures. Subsequent studies rapidly expanded into environmental applications [139], demonstrating the effectiveness of MXene aerogels in pollutant adsorption, oil–water separation, and desalination. Their hierarchical porosity and chemically active surfaces promote efficient contaminant capture, yielding great removal efficiencies [140]. Concurrently, MXene aerogels have been incorporated into diverse sensing and biomedical platforms, such as physical and biochemical sensors, controlled drug delivery systems, biomolecule enrichment, and wearable health‐monitoring devices, benefiting from their flexibility, biocompatibility, and tunable electrical responses [141, 142, 143].

MXene‐based aerogels are reshaping electronic technologies through applications in EMI shielding and MW absorption, while their rich surface chemistry and high density of catalytically active sites enable efficient electro‐ and photo‐catalytic processes for energy conversion and pollutant mitigation [142, 144]. Their use has also expanded into thermal management, including personal wearable heating and cooling textiles as well as industrial thermal insulation, benefiting from their lightweight and thermally adaptive characteristics [145, 146]. This progression reflects a broader transformation in materials science, in which MXene aerogels no longer belong to a single discipline but instead function as a multidisciplinary platform addressing challenges in energy storage, environmental sustainability, healthcare, and industrial technologies. Their versatility arises from inherent properties like metallic‐level electrical conductivity and tunable mechanical and surface features, which foster cross‐disciplinary innovation. Future advances are expected to emerge from integrated applications, such as self‐powered biosensing systems combining energy storage and biomedicine, or solar‐driven desalination aerogels that merge catalytic functionality with environmental engineering, positioning MXene aerogels as key materials for sustainable technologies.

### 7.1 Energy Storage Systems

**FIGURE 14:**
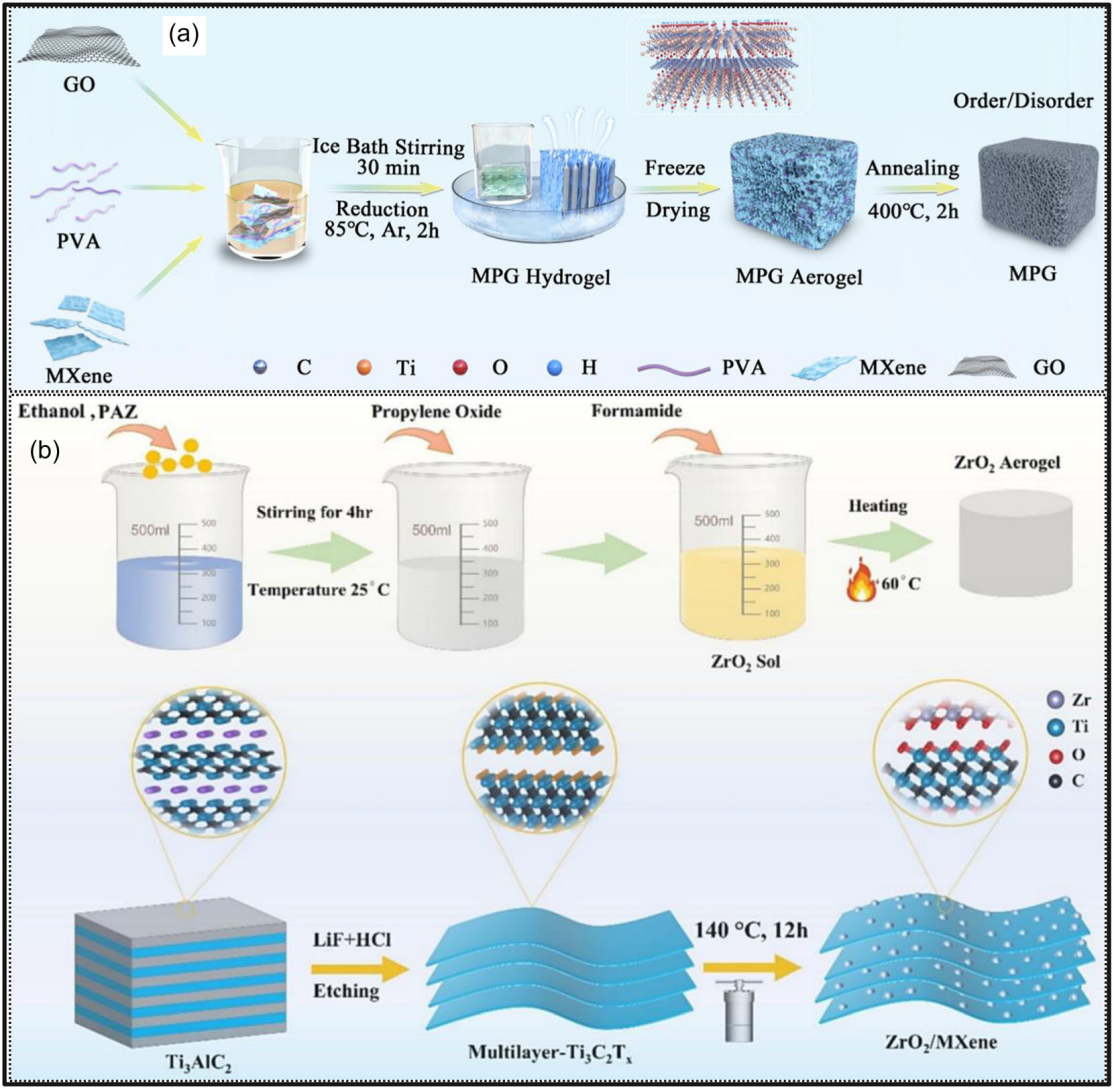
(a) Representation of an ultra‐stable MXene/PVA/rGO composite aerogel with high electrochemical behavior for SC applications. Adapted from [154]. Copyright 2025, American chemical society. (b) Schematic depiction of the interfacial interaction within a ZrO_2_ aerogel/MXene composite used as an anode for LIBs. Adapted from [155]. Copyright 2025, Elsevier B.V.

The outstanding electrochemical behavior of MXene aerogels in energy storage applications is primarily attributed to their distinctive structural and physicochemical features. Their 3D porous, interconnected frameworks efficiently suppress the restacking of MXene NSs, thereby maintaining continuous ion transport channels and enhancing electrolyte penetration. In parallel, the inherently high conductivity of MXene networks promotes efficient electron transfer across the electrode matrix [147, 148]. Furthermore, a tunable hierarchical pore system, where mesopores facilitate ion diffusion and macropores serve as ion reservoirs, significantly improves charge storage kinetics [149, 150, 151]. In addition, the presence of abundant surface functional groups offers active sites for electrochemical reactions and enables integration with polymers, C‐based materials, or metal oxides, leading to enhanced mechanical robustness and cycling durability. Collectively, these characteristics render MXene aerogels promising candidates for advanced SCs and battery technologies requiring high‐rate performance, long‐term durability, and high specific capacitance [10, 152, 153]. Wang and coworkers [154] fabricated a 3D MXene/PVA/rGO composite aerogel employing an amalgamation of directional freeze‐drying and a one‐step hydrothermal treatment to mitigate MXene NSs restacking and enhance ion passage (Figure 14a). In this structure, rGO served as an electrically conductive scaffold, while PVA improved mechanical integrity and contributed additional pseudocapacitance after carbonization. This synergistic architecture facilitated efficient electrolyte infiltration and enhanced electrochemical performance. As a result, the composite aerogel exhibited great capacitance, favorable energy density, and excellent cycling durability, highlighting its potential as an advanced electrode material for next‐generation SCs. Hierarchical MOFs@MXene aerogels can be transformed via thermal treatment into hollow CoS nanobox architectures integrated within a conductive MXene framework. The resulting (CoS NP@NHC)@MXene exhibit a high reversible capacity of 1145.9 mAh g^−1^ at a current density of 1 A g^−1^ over 800 charge–discharge cycles in LIBs, while also showing strong electrochemical behavior in spectroscopy, and sodium ion batteries (SIBs) and PIBs, highlighting the structural adaptability of these aerogels [156]. In a related study, full Li‐ion capacitors assembled using Fe_2_O_3_ hollow nanospheres embedded in the aerogel‐type MXene network and paired with a dual‐doped porous C cathode achieved an energy density of 216 Wh kg^−1^ at a power density of 400 W kg^−1^, while retaining 96.5 Wh kg^−1^ at 20 kW kg^−1^, demonstrating the simultaneous realization of high energy and power output [157]. Tan et al. [155]. reported the fabrication of a ZrO_2_ aerogel/MXene architecture via a wet‐chemical synthesis to address MXene NSs restacking and limited charge storage capacity (Figure 14b). Strong interfacial interactions within the heterostructure promote the formation of interconnected open pores and efficient transport pathways, thereby enhancing electron conduction and ion diffusion. As a result, the electrode exhibits a high reversible capacity of 561.1 mAh g^−1^ at a current density of 2 A g^−1^ after 400 cycles. DFT calculations further reveal increased ion adsorption energies, reduced diffusion barriers, and improved charge–discharge kinetics. These findings highlight the potential of heterostructural design strategies for advancing MXene‐based materials in next‐generation energy storage systems. To further highlight the advantages of MXene‐based aerogels, a quantitative comparison with other representative 3D conductive aerogels is summarized in Table 1.

**TABLE 1:** Comparison of MXene aerogels with other 3D conductive aerogels for energy storage applications.

Material system	Structural characteristics	Electrical conductivity	Electrochemical performance	Major advantages	Key limitations	Representative features
MXene aerogels	3D porous networks of stacked 2D NSs; tunable interlayer spacing; surface terminations (^‐^O, ^‐^OH, ^‐^F)	High (up to ∼10^3^–10^4^ S m^−1^)	Capacitance: 300–500 F g^−1^; Energy density: 20–60 Wh kg^−1^	Metallic conductivity; rich surface chemistry; strong pseudocapacitance; hydrophilicity enabling ion accessibility	Prone to oxidation; structural instability without reinforcement; conductivity‐porosity trade‐off	Fast charge transfer; tunable interfacial chemistry; high redox activity
MXene‐based hybrid aerogels (MXene/rGO, MXene/CNT, etc.)	Hierarchical porous frameworks with multiscale connectivity; suppressed restacking	Moderate‐High	Capacitance: 400–600 F g^−1^; Energy density: 30–80 Wh kg^−1^	Synergistic effects (conductivity + mechanical strength); improved stability; enhanced ion transport pathways	Increased system complexity; possible interfacial resistance; reduced MXene content	Optimized electron/ion transport; improved cycling stability
Graphene aerogels	3D interconnected C networks; high SSA; mostly meso‐/macroporous	Moderate (∼10–10^2^ S m^−1^)	Capacitance: 100–300 F g^−1^; Energy density: 5–20 Wh kg^−1^	High SSA; excellent chemical stability; lightweight; good mechanical resilience	Limited pseudocapacitance; hydrophobicity limits electrolyte interaction	Electric double‐layer capacitance dominant
CNT aerogels	Entangled 1D fibrous conductive networks; open porous structure	High (∼10^2^–10^3^ S m^−1^)	Capacitance: 50–200 F g^−1^; Energy density: 5–15 Wh kg^−1^	Excellent electrical pathways; high mechanical flexibility; fast electron transport	Low SSA utilization; limited electrochemical activity	Highly conductive scaffolds; good for current collectors
Graphene/CNT hybrid aerogels	Hierarchical hybrid networks combining 1D and 2D C structures	Moderate‐High	Capacitance: 200–400 F g^−1^; Energy density: 10–40 Wh kg^−1^	Synergistic conductivity and structural stability; improved electron transport	Still limited redox activity; performance relies on architecture optimization	Balanced conductivity and porosity
Conductive polymer‐based aerogels (e.g., PEDOT, PANI hybrids)	Polymer‐coated porous frameworks; often combined with C supports	Moderate	Capacitance: 300–700 F g^−1^ (pseudocapacitive)	High pseudocapacitance; tunable chemistry; flexible	Poor cycling stability; swelling/shrinkage; lower conductivity than MXenes	Redox‐active charge storage

**FIGURE 15:**
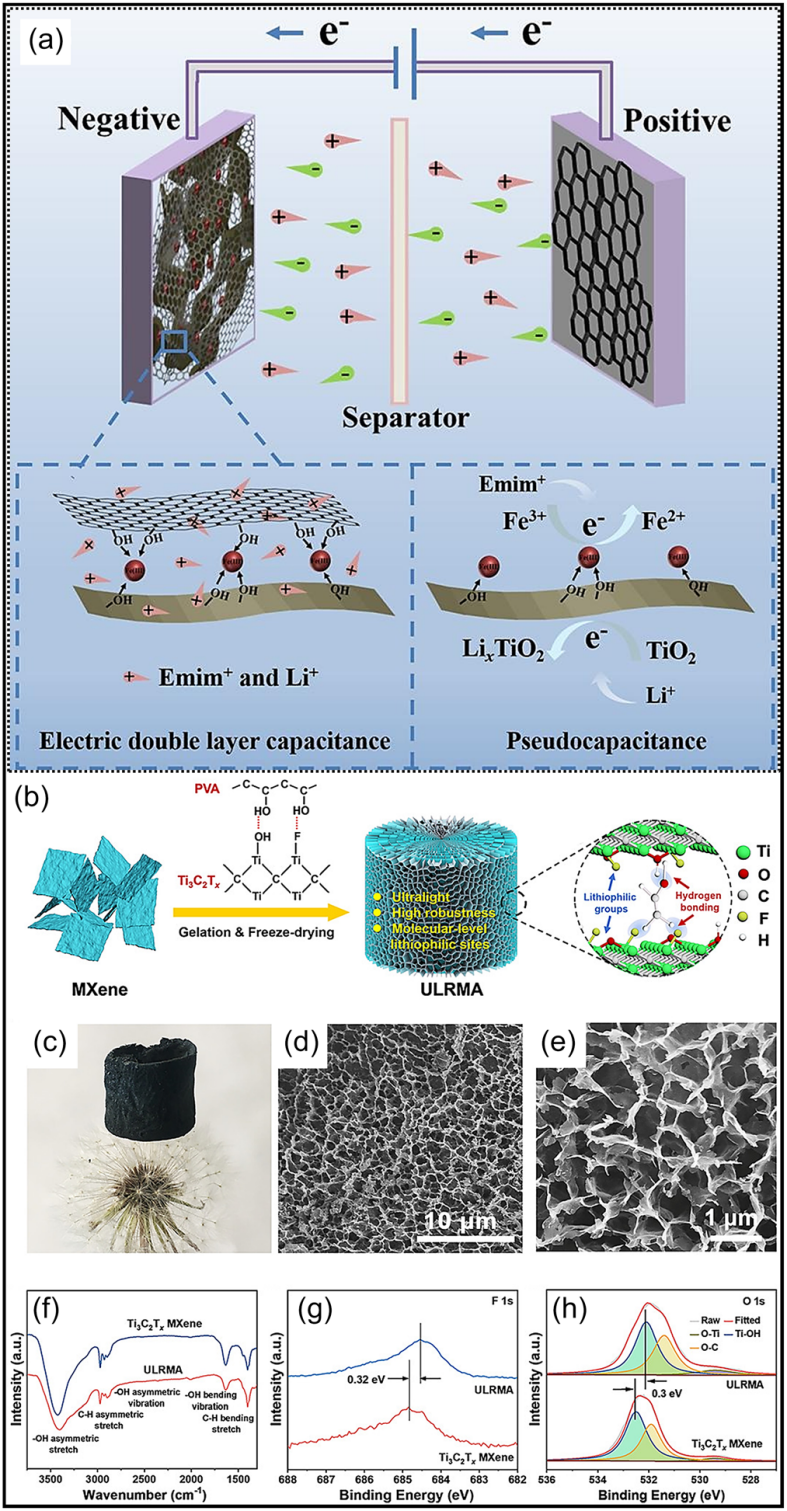
(a) Illustration of Fe^3+^/Fe^2+^‐aided assembly of MXene/TiO_2_‐graphene aerogels designed for IL‐based SCs. Adapted from [158]. Copyright 2023, Elsevier B.V. (b) Schematic overview of synthesis process for ultralight MXene‐based aerogels (ULRMA). (c) Optical pictures highlighting the extremely low density of ULRMA, demonstrated by a monolith supported on a dandelion flower. (d,e) SEM micrographs showing the 3D framework of ULRMA. (f) FTIR spectra together with, (g) F 1s, and (h) O 1s XPS spectra comparing ULRMA and pristine Ti_3_C_2_T_*x*_ MXene. Adapted from [159]. Copyright 2021, Wiley‐VCH.

A ferric ion‐directed self‐assembly approach has been employed to fabricate MXene/TiO_2_‐graphene aerogels, in which an amorphous TiO_2_ layer contributes additional pseudocapacitive behavior. When paired with a wide voltage window ionic liquid (IL) electrolyte, asymmetric SCs based on this architecture delivered an energy density of 54 Wh kg^−1^ at a power density of 1737 W kg^−1^ (Figure 15a) [158]. In a related strategy, crumpled MXene aerogels formed via Mg^2+^‐mediated assembly exhibited a high SSA of 140.5 m^2^ g^−1^ alongside an electrical conductivity of 758 S m^−1^. These combined properties enabled multifunctional applications, including capacitive deionization with a salt adsorption capacity of 33.3 mg g^−1^ and on‐chip micro SCs achieving an areal capacitance of 409 mF cm^−2^ [160]. Qiu's group [159] reported a rapid, room‐temperature H‐bonding approach to gel Ti_3_C_2_T_*x*_ MXene with PVA within 1 h, producing an ultralight aerogel that functions as a Li‐metal anode (Figure 15b). The exceptionally low density of the resulting monolith is illustrated by its ability to rest on a flower petal (Figure 15c). Structural characterization (Figure 15d,e) reveals a hierarchical porous architecture in which crumpled MXene NSs assemble into interconnected micrometer‐scale channels that facilitate efficient ion and electron transport. Evidence for strong H‐bonding between PVA hydroxyl groups and MXene surface terminations is provided by the red‐shift and broadening of the ^‐^OH absorption bands following gelation (Figure 15f). Further analysis (Figure 15g,h) shows that PVA suppresses MXene restacking, as indicated by the shift of the (002) diffraction peak, and forms F/O‐hydroxyl H‐bonds, reflected by a ∼0.3 eV binding energy shift. This synergistically engineered scaffold enables fast‐charging and dendrite‐free Li‐metal batteries [159]. Together, these investigations highlight the critical role of rational structural engineering, through strategies such as freeze casting, templating, heterostructure construction, and ion‐mediated assembly, in unlocking the electrochemical capabilities of MXene aerogels and advancing next‐generation high‐energy and power devices.

**FIGURE 16:**
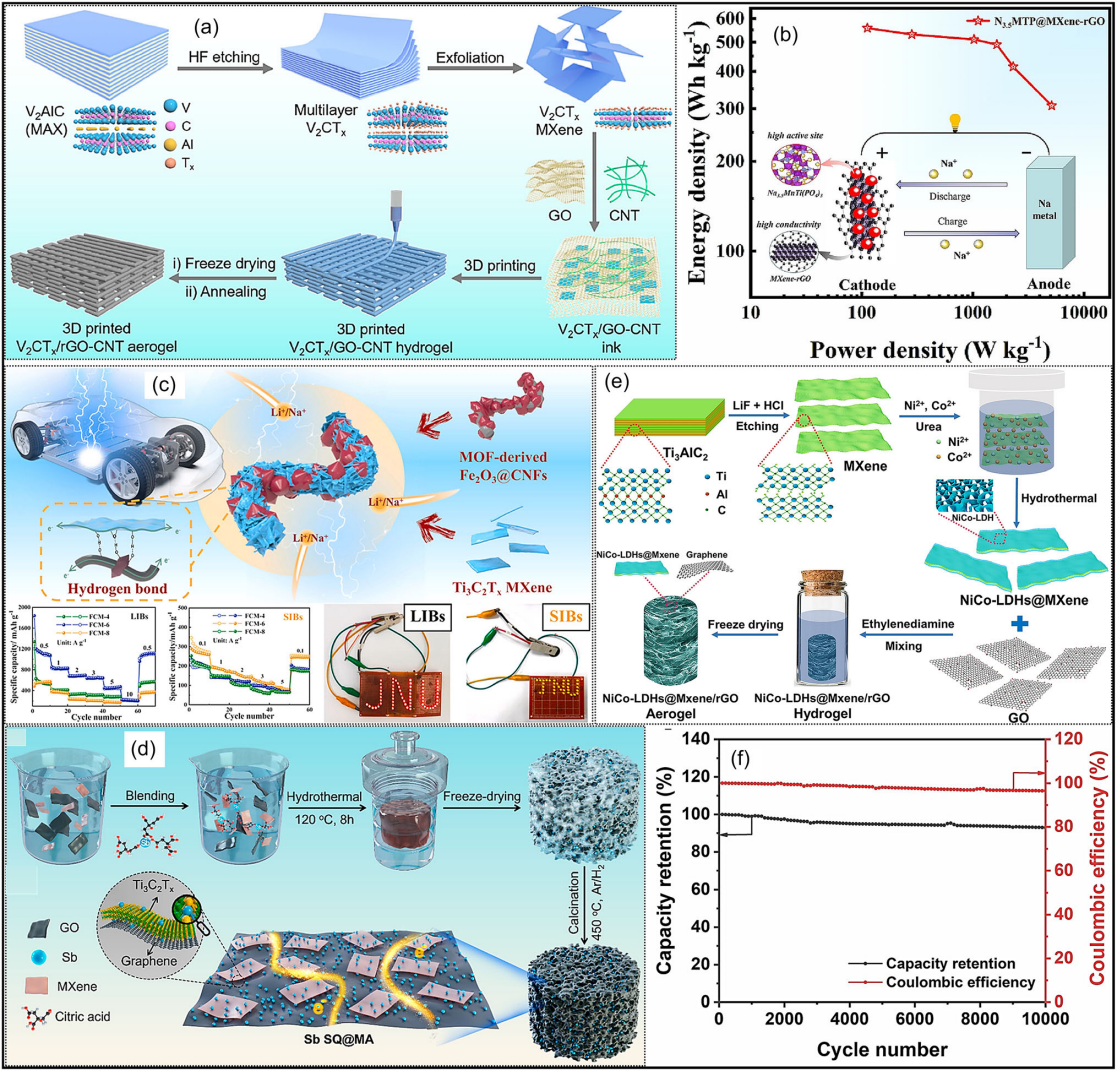
(a) 3D‐printed V_2_CT_*x*_/rGO‐CNT MXene microgrid aerogel as a sodiophilic host for stable Na metal anodes with great areal loading. Adapted from [161]. Copyright 2022, American chemical society. (b) MXene‐rGO aerogel anchored Na_3.5_MnTi(PO_4_)_3_ cathode for Na^+^ storage. Adapted from [162]. Copyright 2023, Elsevier B.V. (c) Bead‐like CNF heterostructured Ti_3_C_2_T_*x*_ aerogel derived from MOFs for improved Li^+^/Na^+^ storage. Adapted from [163]. Copyright 2024, Elsevier B.V. (d) Sb quantum‐dot decorated MXene aerogel for high‐performance PIBs. Adapted from [164]. Copyright 2022, American chemical society. (e,f) NiCo_2_‐LDHs@MXene/rGO composite aerogel design and electrochemical behavior in hybrid SCs. Adapted from [165]. Copyright 2020, Elsevier B.V.

MXene‐based aerogels have expanded far beyond their early applications in Li‐ion systems, serving as versatile networks for next‐generation SIBs and hybrid electrode architectures. Huang's team [161] addressed the intrinsic limitations of Na‐metal anodes by developing a 3D‐printed V_2_CT_*x*_/rGO‐CNT microgrid aerogel with sodiophilic surface groups that guide homogeneous Na nucleation and growth (Figure 16a). This architected host supports dendrite‐free Na plating/stripping for more than 3000 h at 10 mAh cm^−2^ and maintains structural and electrochemical stability even at current densities up to 50 mAh cm^−2^. When paired with Na_3_V_2_(PO_4_)_3_ cathodes, the resulting full cells delivered a reversible capacity of 86 mAh g^−1^ after 400 charge–discharge cycles. In a related effort, Zhu and coworkers [162] improved Na^+^ cathode performance by directly growing a NASICON‐type Na_3.5_MnTi(PO_4_)_3_ phase on a conductive MXene/rGO aerogel scaffold (Figure 16b). The interconnected 3D network suppresses particle agglomeration, mitigates lattice strain during cycling, and accelerates electron/ion transport, enabling a high capacity of 189 mAh g^−1^ at 50 mA g^−1^, outstanding durability over 5000 cycles, and robust low‐temperature performance (98 mAh g^−1^ at ‐30°C).

Recent progress by Long and colleagues [163] demonstrated suppression of MXene NSs restacking through the integration of MOF‐derived Fe_2_O_3_ NPs within N‐doped CNFs, forming a porous Ti_3_C_2_T_*x*_ aerogel that facilitates rapid ion transport while accommodating volume variations during cycling (Figure 16c). This heterostructured aerogel exhibits high‐rate storage capabilities, delivering 202 mAh g^−1^ at 10 A g^−1^ for LIBs and 98 mAh g^−1^ at 5 A g^−1^ for SIBs. Extended cycling tests further show capacities of 401 mAh g^−1^ after 2000 cycles for LIBs and 197 mAh g^−1^ after 1000 cycles for SIBs. In a related advancement, Wang's team [164] reported Ti_3_C_2_T_*x*_ aerogels decorated with Sb quantum dots (∼5 nm) as high‐performance PIB electrodes (Figure 16d). Atomic‐scale Sb dispersion modulates the electronic architecture, accelerating interfacial charge transport and enhancing K^+^ storage kinetics. The MXene aerogel framework supplies abundant anchoring sites, preserves structural integrity, and ensures efficient electron movement, enabling high Sb loading (60.3 wt %) and short ion diffusion pathways that together promote effective K^+^ storage. This approach highlights the potential of interfacial engineering for tailoring MXene aerogels toward advanced electrochemical advantages.

Yang's group [165] reported the fabrication of a 3D NiCo_2_‐LDHs@MXene/rGO aerogel via a combination of hydrothermal synthesis and wet‐chemical assembly of MXene NSs, NiCo_2_‐LDHs, and rGO (Figure 16e). Owing to its conductive and porous framework, the aerogel delivers a specific capacity of 332.2 mAh g^−1^ at 1 A g^−1^ and retains 87.5% of its capacity after 5000 charge–discharge cycles. When configured as a hybrid SCs using an MXene/rGO negative electrode (Figure 16f), the device achieves an energy density of 65.3 Wh kg^−1^ at a power density of 700 W kg^−1^, while maintaining 92.8% capacity retention over 10 000 cycles. These results demonstrate an economical and scalable route toward high‐performance MXene‐based electrode architectures for advanced energy‐storage applications. Taken together, recent advances demonstrate that rational design of MXene aerogel architectures, through approaches such as sol–gel growth on MXene‐rGO frameworks, 3D printing of sodiophilic networks, or MOF‐derived bead incorporation, can enable high capacities, excellent low‐temperature performance, suppression of dendrite formation, and exceptional cycling stability across diverse battery chemistries, positioning MXene aerogels as a versatile platform for next‐generation energy‐storage systems.

### 7.2 Wastewater Purification

**FIGURE 17:**
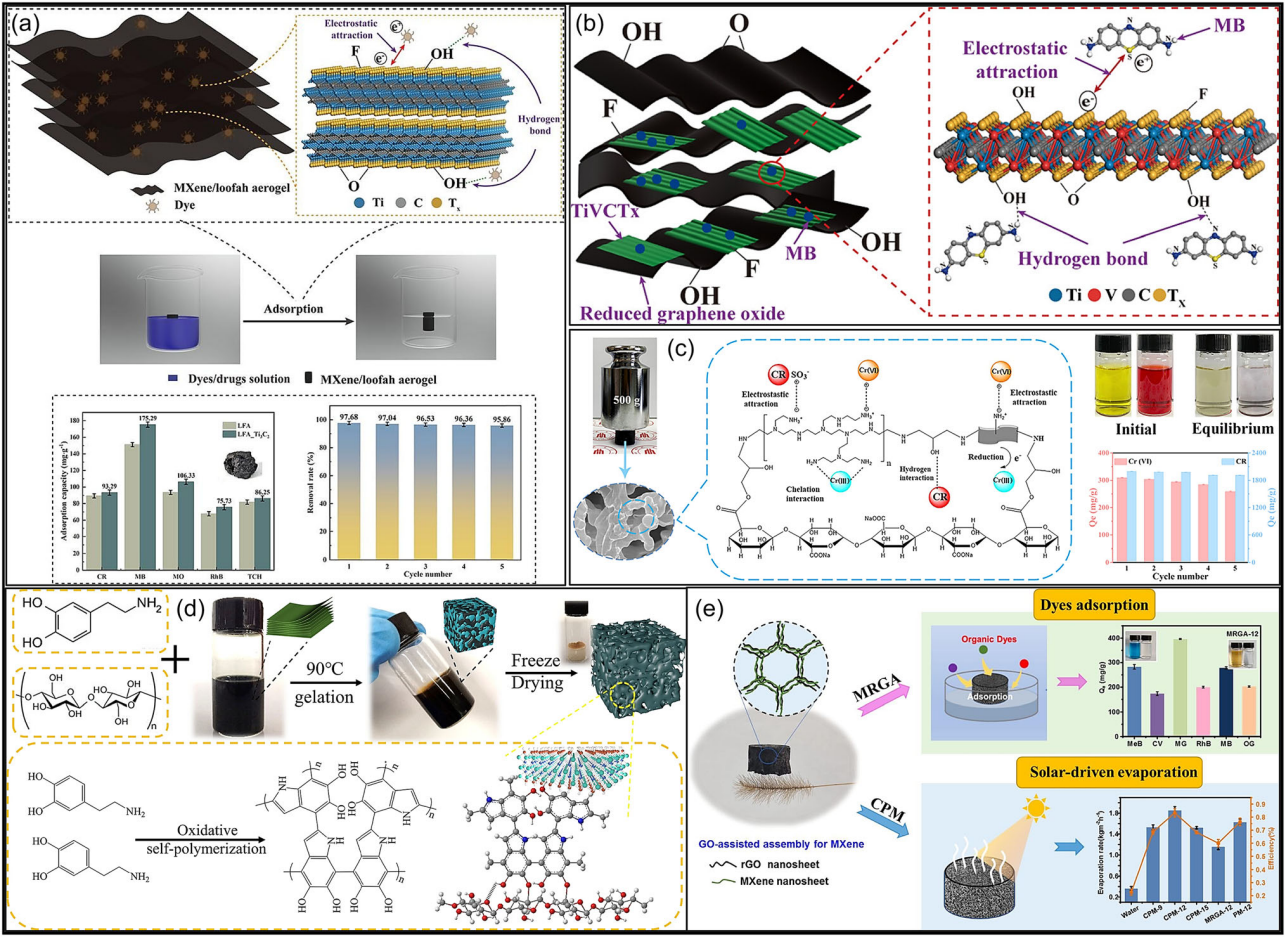
(a) Loofah‐derived C aerogel loaded with Ti_3_C_2_T_*x*_ for organic contaminant adsorption in wastewater. Adapted from [173]. Copyright 2025, Elsevier B.V. (b) TiVCT_*x*_/graphene NSs aerogels for aqueous organic pollutant removal. Adapted from [171]. Copyright 2024, American chemical society. (c) MXene/PEI‐modified SA aerogel showing ultrahigh CR adsorption. Adapted from [174]. Copyright 2021, Elsevier B.V. (d) Cellulose‐MXene aerogel functionalized with PDA for efficient MB removal. Adapted from [175]. Copyright 2021, Springer Nature. (e) Multifunctional MXene/rGO aerogels for solar‐driven water evaporation and wastewater purification. Adapted from [170]. Copyright 2024, Elsevier B.V.

MXene aerogels feature hydrophilic frameworks with low tortuosity and hierarchically organized pores, which collectively accelerate mass transport and maximize exposure of active sites for pollutant uptake. Surface terminations such as Ti‐O and ‐OH, together with expanded interlayer galleries, favor strong complexation and inner‐sphere binding with heavy metal ions including Pb^2+^ and Cd^2+^. In addition, negatively charged surfaces and π‐π as well as H‐bonding interactions facilitate efficient adsorption of cationic dyes and aromatic pharmaceutical compounds [166]. Adjustable interlayer distance and Donnan exclusion effects further enable selective ion sieving and low‐potential electrosorption for desalination. For oil–water separation, rough, underwater superoleophobic pore surfaces destabilize emulsions and suppress oil adhesion, enabling gravity‐ or pressure‐driven demulsification with limited fouling [167, 168, 169]. The high conductivity of MXene‐based aerogels enables the integration of adsorption with insitu electrochemical oxidation and reduction processes, thereby accelerating dye decolorization and pharmaceutical degradation. Conductivity also allows polarity‐switch‐assisted regeneration, promoting ion desorption and enabling repeated reuse. Collectively, these attributes result in rapid removal kinetics, high uptake capacities, broad selectivity toward dyes, heavy metals, salts, oils, and pharmaceuticals, as well as effective regeneration under complex wastewater conditions [170, 171, 172]. For example, a loofah‐derived C aerogel fabricated via hydrothermal carbonization and subsequently loaded with Ti_3_C_2_T_*x*_ exhibited adsorption capacities of 175.29 mg g^−1^ for methylene blue (MB), 75.73 mg g^−1^ for Rhodamine B (RhB), 106.33 mg g^−1^ for methyl orange (MO), 93.29 mg g^−1^ for Congo red (CR), and 86.25 mg g^−1^ for tetracycline hydrochloride (TCL), while maintaining excellent recyclability (Figure 17a) [173].

In a related study, TiVCT_*x*_ MXene/graphene NSs aerogels (TiVCT_*x*_/GAs) fabricated via hydrothermal followed by freeze‐drying exhibited high adsorption capacities of 319.67, 303.45, 229.97, 217.87, and 283.38 mg g^−1^ for MB, RhB, CR, and MO, respectively (Figure 17b) [171]. Sodium alginate (SA) aerogels modified with MXene and polyethyleneimine (PEI) demonstrated exceptionally high uptake capacities of 538.97 mg g^−1^ for Cr(VI) and 3568 mg g^−1^ for CR, which were attributed to the mechanically robust dual‐network architecture and intrinsic antibacterial properties (Figure 17c) [174]. In addition, MXene/C foam hybrid aerogels achieved adsorption capacities of 356.97 mg g^−1^ for MB and 647.75 mg g^−1^ for CR, with distinct adsorption behaviors observed for monomeric and dimeric dye species [176]. Under high‐salinity conditions, functionalized cellulose/MXene aerogels maintained a MB uptake of 168.93 mg g^−1^ (Figure 17d) [175], while Ti_3_C_2_T_*x*_/SA aerogels enabled nearly complete cationic dye removal within 10 min, reaching a capacity of 571.43 mg g^−1^ [177]. Jiang and coworkers [170] developed robust and multifunctional MXene/rGO aerogels further functionalized with polydopamine (PDA)‐chitosan via a deposition process combined with ice‐templated self‐assembly (Figure 17e). The resulting aerogels exhibit a thin yet mechanically stable 3D macroporous architecture, which enables efficient dye adsorption, achieving a capacity of 396.05 mg g^−1^ for malachite green (MG). By lowering the water evaporation enthalpy to 1617.18 J g^−1^, the aerogels delivered an exceptional solar steam generation rate of 1.86 kg m^−2^ h^−1^ and a high solar‐to‐vapor efficiency of 83.28% under one‐sun illumination. These performances arise from enhanced GO reduction and the synergistic effects of PDA and chitosan. In addition, the aerogels demonstrated effective oil–water separation, highlighting their potential for simultaneous solar‐driven desalination and wastewater treatment [64].

These aerogels typically follow Langmuir isotherms and pseudo‐2nd‐order kinetic behavior, with adsorption driven by electrostatic interactions, H‐bonding, and interlayer diffusion. Such mechanisms enable spontaneous uptake and endow the materials with recyclability and versatility, making them promising candidates for sustainable wastewater treatment. MXene‐based aerogels have emerged as highly effective and sustainable adsorbents for the removal of heavy metal ions and other ionic species, such as phosphates, from wastewater, contaminants that pose serious environmental risks and limit resource recovery. Owing to their large SSA, tunable surface chemistry, and robust 3D porous networks, these materials exhibit excellent adsorption capacity, selectivity, and recyclability in environmental remediation applications. For instance, a composite aerogel composed of leather collagen and MXene@PDA/oxidized SA achieved Cr(VI) adsorption capacities of 80.73, 100.80, and 112.86 mg g^−1^ at initial concentrations of 30, 70, and 100 mg L^−1^, respectively, within 1 h. The adsorption behavior was well described by a two‐level kinetic model and the Freundlich isotherm, and performance was further enhanced under solar illumination due to photothermal effects [178].

**FIGURE 18:**
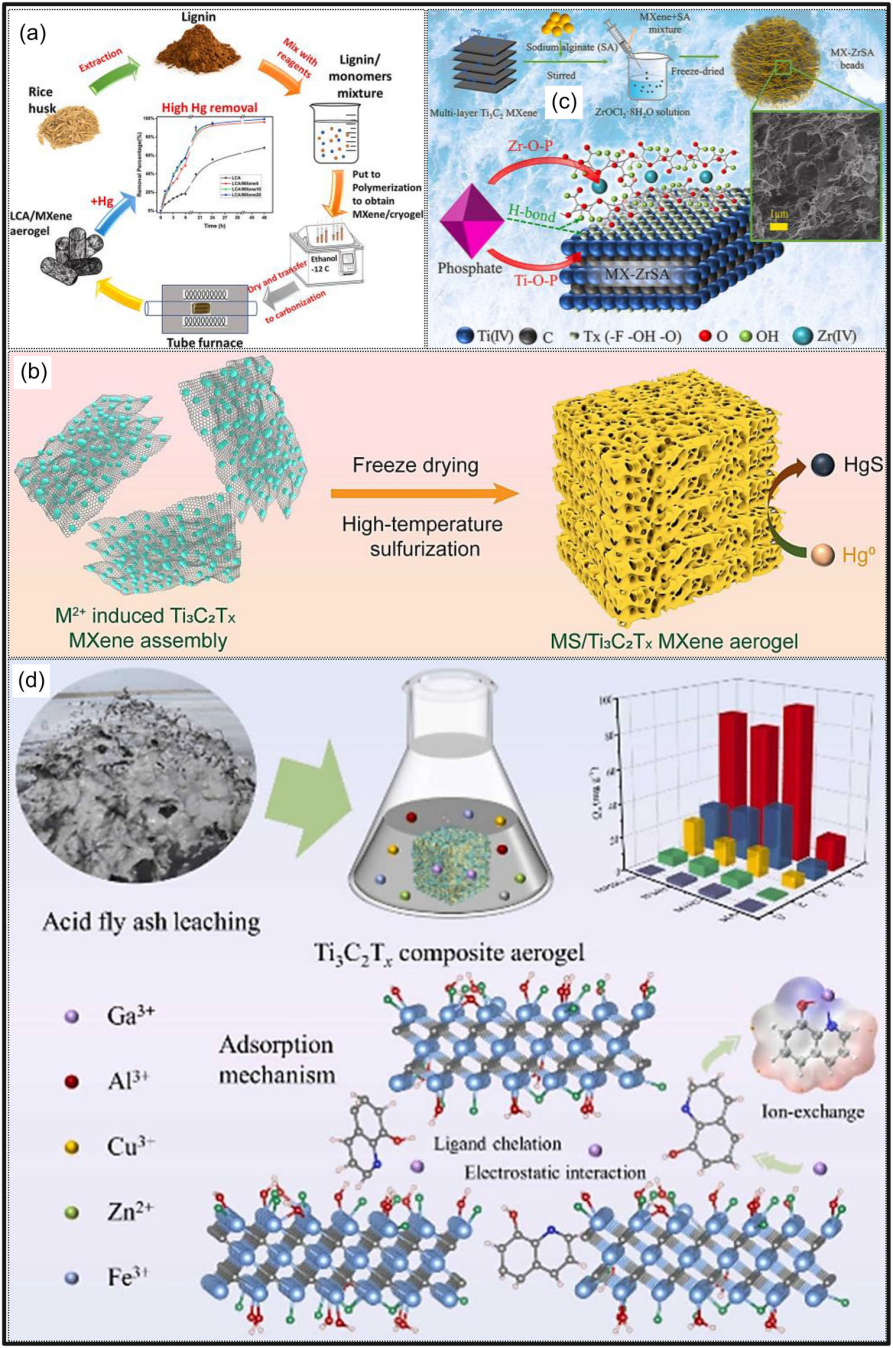
(a) MXene‐modified C aerogel fabricated from renewable biomass for aqueous Hg^2+^ removal. Adapted from [180]. Copyright 2025, Elsevier B.V. (b) Metal sulfide‐functionalized Ti_3_C_2_T_*x*_ aerogel with interconnected porosity for gas‐phase Hg^2+^ adsorption. Adapted from [181]. Copyright 2023, Elsevier B.V. (c) 3D Zr‐alginate aerogel embedded with Ti_3_C_2_ as an efficient phosphate scavenger. Adapted from [182]. Copyright 2023, Elsevier B.V. (d) Dual‐crosslinked Ti_3_C_2_T_*x*_ composite aerogels enabling selective Ga^3+^ adsorption during acid fly ash leaching. Adapted from [183]. Copyright 2024, Elsevier B.V.

A cellulose/MXene/alginate aerogel prepared from date palm leaf biomass and reinforced via Ca^2+^‐crosslinked SA demonstrated effective heavy‐metal adsorption, with capacities of 72.9, 114.4, 92.9, and 123.9 mg g^−1^ for As, Cd, Ni, and Zn, respectively. Optimal adsorption was observed within a pH window of 6–8, and the aerogel maintained stable performance over three consecutive regeneration cycles [179]. In a related work, a rice husk lignin‐derived aerogel containing 20 wt % MXene achieved a Hg^2+^ uptake of 135.8 mg g^−1^, outperforming several commercial adsorbents while retaining high efficiency in tap water environments (Figure 18a) [180]. Furthermore, a CuS/Ti_3_C_2_T_*x*_ aerogel synthesized through Cu^2+^‐induced assembly followed by sulfurization exhibited exceptional Hg capture, reaching 90 596.4 g m^−3^ with a rapid adsorption rate of 120.64 g m^−3^ min^−1^, which was attributed to its large SSA and homogeneous dispersion of CuS NPs (Figure 18b) [181]. Prussian blue decorated MXene aerogel spheres exhibited outstanding Cs^+^‐ion capture, reaching an adsorption capacity of 315.91 mg g^−1^, while maintaining excellent selectivity in simulated seawater and across a wide pH range [184]. In a related study, a Ti_3_C_2_/Zr‐crosslinked SA aerogel delivered a remarkable phosphate adsorption capacity of 492.55 mg g^−1^, primarily governed by chemisorption through Zr‐O‐P and Ti‐O‐P coordination, and demonstrated robust selectivity in complex aqueous systems (Figure 18c) [182]. Additionally, a Ti_3_C_2_T_*x*_ MXene aerogel applied to acidic fly ash leachates achieved a Ga^3+^ uptake of 132.52 mg g^−1^ and retained 95.65% of its original adsorption capacity after five regeneration cycles (Figure 18d) [183]. Highly selective uranium (U) removal was realized using a ZIF‐67‐modified MXene/GO aerogel, which delivered a U(VI) uptake of 869.59 mg g^−1^ at pH 3, driven by cooperative interactions between oxygenated functional groups and Co‐OH active sites [185]. Overall, MXene‐derived aerogels typically conform to Langmuir or Freundlich isotherms and follow pseudo‐2^nd^‐order kinetics, reflecting adsorption mechanisms dominated by chemisorption, electrostatic attraction, and ion exchange, thereby highlighting their promise for sustainable water purification and resource recovery applications.

**FIGURE 19:**
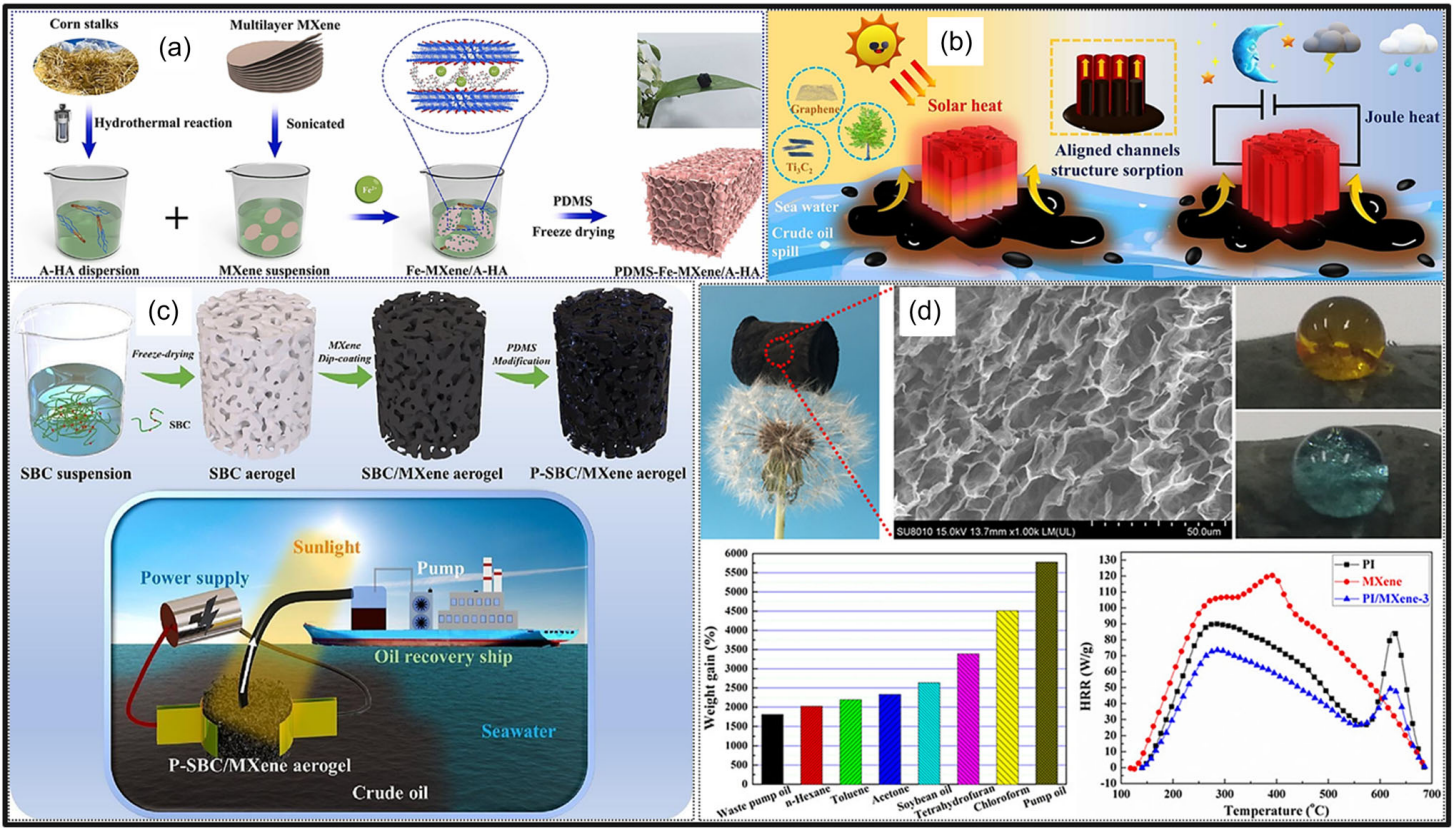
(a) Fire‐retardant MXene‐based hybrid aerogels designed for high‐efficiency oil–water separation. Adapted from [186]. Copyright 2023, Elsevier B.V. (b) Cellulose aerogel modified with MXene and graphene enabling photo‐ and electro‐assisted removal of large‐viscosity crude oil under all‐weather conditions. Adapted from [187]. Copyright 2023, Elsevier B.V. (c) Environmentally benign bacterial cellulose (BC)/MXene aerogel exhibiting strong photothermal and electrothermal transformation for effective crude oil‐seawater separation. Adapted from [188]. Copyright 2024, Elsevier B.V. (d) Lightweight and mechanically robust PI/MXene aerogels with hydrophobic and flame‐resistant properties for oil–water separation. Adapted from [189]. Copyright 2019, American chemical society.

MXene‐based aerogels have emerged as highly effective materials for oil–water separation, owing to their robust mechanical integrity, durable hydrophobic surfaces, and strong photothermal response that enables efficient removal of oils and hydrophobic organic contaminants from wastewater. These advanced architectures offer a sustainable strategy for mitigating oil spills, which represent a significant threat to marine ecosystems and human health. For example, a hydrophobic PDMS‐Fe‐MXene/A‐HA aerogel fabricated via Fe^2+^‐induced self‐assembly of MXene NSs exhibited long‐term water repellency (WCA ≈ 135°), high adsorption uptake (64–121 g g^−1^), and exceptional separation flux (23478 L h^−1^ m^−2^), together with excellent reusability (Figure 19a) [186]. In another study, a PI NF/MXene composite aerogel displayed ultralow density (9.98 mg cm^−3^), extreme compressibility (till 90% strain), and high oil uptake (55.85–135.29 g g^−1^), outperforming spider‐web like structures in adsorption efficiency [190].

A Ti_3_C_2_T_*x*_/gelatin aerogel with vertically oriented microchannels and hydrophilic yet oleophobic surface characteristics achieved a solar‐driven evaporation rate of 1.7 kg m^−2^ h^−1^ under one‐sun conditions. The material also exhibited excellent structural stability in wet environments, allowing efficient freshwater extraction from oil–water mixtures [191]. Separately, wood‐mimetic MXene aerogels constructed from surface‐modified cellulose nanocrystals and waterborne PU demonstrated high mechanical resilience, maintaining 76.2% strain recovery after 100 compression cycles, along with pronounced superhydrophobicity (CA ≈ 152°). These aerogels showed strong oil sorption capacities of 63 g g^−1^ for low‐viscosity oils and 24.5 g g^−1^ for heavy crude under solar irradiation, while retaining 76% of their initial performance after five reuse cycles (Figure 19b) [187]. In addition, a green P‐SBC/MXene aerogel delivered rapid photothermal heating (93°C at 1 kW m^−2^) and electrothermal heating (124°C at 3 V), enabling an ultrahigh crude oil permeation rate of 630 kg m^−2^ h^−1^ and stable oil‐seawater separation across varied environmental conditions (Figure 19c) [188].

Manohar and coworkers [192] reported a robust ternary NFs membrane fabricated by electrospinning polyacrylonitrile (PAN) with hydrophilic candle‐soot NPs and MXene NSs to treat oil‐polluted water. Owing to the synergistic coupling between surface‐modified MXene and hydrophilic soot NPs, the membrane achieved an underwater oil CA of 155°, significantly exceeding that of analogous binary membranes (≈130°). The membrane delivered high water fluxes of 3813 L m^−2^ h^−1^ for emulsified oil–water systems and 4468 L m^−2^ h^−1^ for immiscible mixtures, while maintaining a separation efficiency close to 99% under gravity‐driven operation. Moreover, over 96% of the initial efficiency was preserved after 15 consecutive cycles, demonstrating strong operational stability and scalability for wastewater purification. In a complementary study, a freeze‐cast and thermally imidized hydrophobic PI/MXene aerogel exhibited a water CA of 119°, an ultralow density of 23 mg cm^−3^, excellent elastic resilience, and high uptake capacities for organic liquids (18–58 times its own weight), alongside good flame resistance and recyclability (Figure 19d) [189]. Together, these MXene‐enabled membranes and aerogels highlight the effectiveness of combining mechanically robust architectures with tailored surface wettability for efficient and sustainable oil–water separation.

### 7.3 Solar‐Assisted Desalination

MXene aerogels have demonstrated outstanding performance in solar‐assisted desalination due to their 3D porous frameworks, which integrate broadband solar absorption with efficient photothermal conversion and localized interfacial heating, thereby minimizing heat losses from conduction and convection. The interconnected meso‐ and macro‐porous networks facilitate rapid capillary transport of water to the evaporation surface and allow vapor to escape freely, while hydrophilic surface terminations enhance interfacial wettability and water flux [193, 194, 195]. In addition, the porous framework supports outward salt diffusion, and Janus‐type designs further localize thermal energy to the illuminated region, mitigating salt accumulation. Incorporation of polymers such as cellulose or PI, as well as carbonaceous or oxide components, including graphene and SiO_2_, improves mechanical integrity, oxidation resistance, pore regulation, surface wettability, and antifouling properties, enabling stable evaporation performance even in high‐salinity or contaminated water sources [196, 197, 198]. Collectively, both pristine and hybrid MXene aerogels enable efficient evaporation, long‐term operational stability, and lightweight, scalable desalination platforms.

**FIGURE 20:**
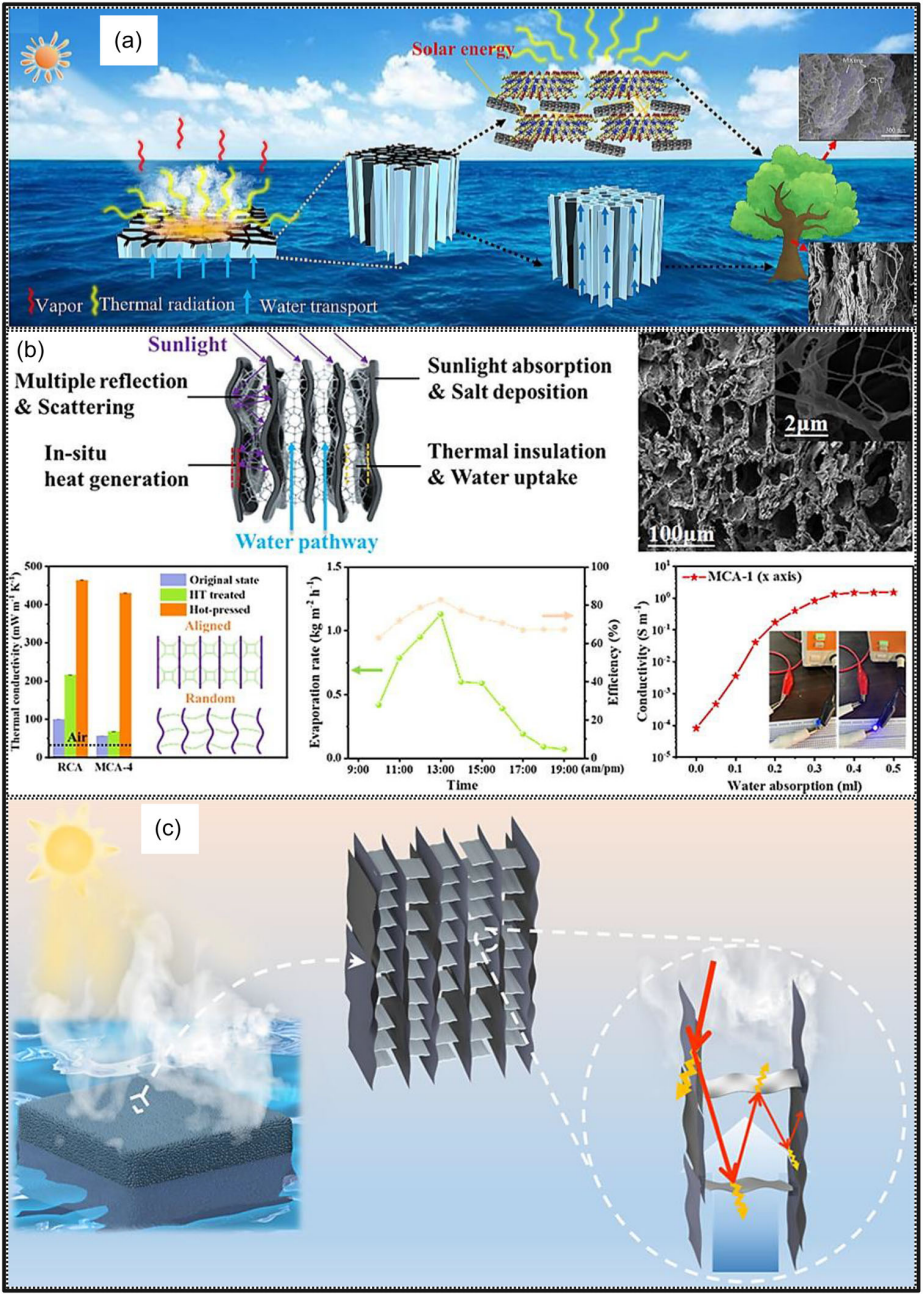
(a) Biomimetic aerogel with vertically aligned channels enabling enhanced photothermal synergy for high‐efficiency solar desalination. Adapted from [199]. Copyright 2023, Elsevier B.V. (b) MXene‐based hybrid aerogel incorporating an interconnected cellulose NFs network, forming a porous architecture for solar‐thermal desalination and humidity‐responsive water transport. Adapted from [200]. Copyright 2023, Elsevier B.V. (c) MXene/rGO aerogel structures employed as photothermal evaporators for solar‐driven seawater desalination. Adapted from [201]. Copyright 2023, American chemical society.

Fan's team [199] developed a biomimetic MXene‐based aerogel evaporator for solar desalination, featuring vertically aligned hydrophilic SA channels integrated with MXene‐CNT layers that function as an efficient light‐absorbing surface (Figure 20a). This architecture enables rapid water delivery and strong photothermal conversion, achieving an evaporation rate of 2.416 kg m^−2^ h^−1^ and a solar‐to‐vapor efficiency of 90.56%. In addition, the aerogel exhibited pronounced antibacterial activity and long‐term operational stability, highlighting its suitability for sustainable freshwater production via solar‐driven desalination. Separately, a Ti_3_C_2_T_*x*_ NSs‐based aerogel, prepared via ion exchange followed by freeze‐drying, exhibited exceptional water vapor uptake (5.86 g g^−1^ at 90% RH and 25°C), rapid heating (temperature rise to 37.4°C in 10 min under 0.5 kW m^−2^ illumination), and low desorption temperatures (<40°C), enabling efficient and energy‐efficient water removal. The material achieved a maximum water collection of 1.125 L kg^−1^, sufficient to meet an adult's minimum daily water requirement, demonstrating its potential utility for water harvesting in arid and semi‐arid regions [202]. In addition, MXene/rGO aerogels have demonstrated exceptional photothermal performance, achieving an energy conversion efficiency as high as 96%. Similarly, CNF/PVA‐based hybrid aerogels exhibited a solar absorptance of 97.9% and a water evaporation efficiency of 82.93%, corresponding to an evaporation rate of 1.13 kg m^−2^ h^−1^ under natural solar irradiation during solar‐driven desalination (Figure 20b) [200]. Furthermore, Chen and colleagues [201] developed a solar‐driven hygroscopic polymer aerogel incorporating MXene as a photothermal absorber for water harvesting. The aerogel displayed strong broadband light absorption, excellent wettability, and multiple confined water states that reduce evaporation enthalpy, enabling an evaporation rate of 2.84 kg m^−2^ h^−1^ under one‐sun illumination and demonstrating potential for seawater desalination and freshwater generation (Figure 20c).

**FIGURE 21:**
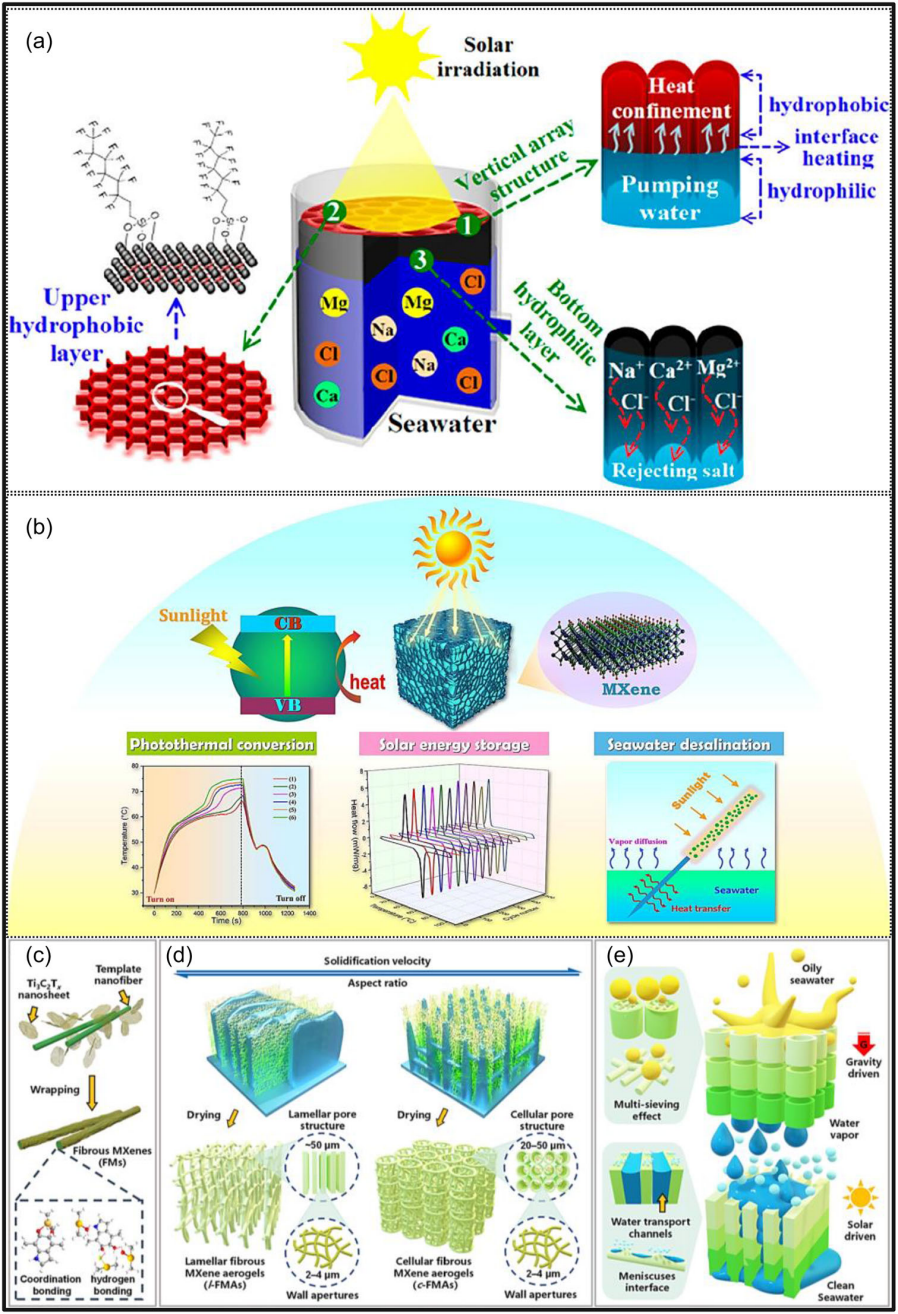
(a) Janus MXene aerogels with vertical alignment for enhanced solar desalination and salt tolerance. Adapted from [203]. Copyright 2019, American chemical society. (b) Phase change PI/MXene composite aerogels applied in solar‐assisted seawater desalination. Adapted from [204]. Copyright 2022, Elsevier B.V. (c–e) Pictorial representation of a modular solar evaporator constructed from NFs MXene aerogels with adjustable pore architectures for high‐performance desalination of oil‐contaminated seawater. Adapted from [205]. Copyright 2023, Springer Nature.

Quan and coworkers [203] reported vertically assembled hydrophilic‐hydrophobic Janus MXene aerogels tailored for solar‐driven desalination (Figure 21a). The layered configuration promotes efficient water supply, suppresses thermal dissipation, and prevents salt crystallization, achieving a light‐to‐heat conversion efficiency of 87%. The system maintained a stable freshwater production rate of 1.46 kg m^−2^ h^−1^ over 15 consecutive days, corresponding to an output of 6 L m^−2^ per day. Similarly, Shurong's group [206] fabricated a Janus CNF/Ti_3_C_2_T_*x*_ aerogel composed of a water‐wicking hydrophilic bottom layer and a photothermally active hydrophobic top layer. This asymmetric architecture ensures continuous water delivery while enhancing solar absorption and thermal confinement. Under 1 sun irradiation, the floating aerogel exhibited an evaporation rate of 2.287 kg m^−2^ h^−1^ with an efficiency of 88.2%, alongside excellent salt tolerance and long‐term stability in seawater, underscoring its potential for sustainable desalination.

Wang and colleagues [204] reported a fully eco‐friendly PI/MXene composite aerogel, further modified with a polyethylene glycol (PEG) coating, for efficient solar‐driven desalination of seawater (Figure 21b). In a related study, Yin et al. [207]. developed a Janus photothermal NFs membrane consisting of a hydrophobic MXene/PDMS surface layer and a hydrophilic PLA/TiO_2_ NPs‐integrated porous scaffold. The hydrophilic sublayer functions as an effective salt‐exclusion barrier, where TiO_2_‐supported microporous channels enable continuous seawater transport while suppressing salt accumulation during solar desalination. Upon solar exposure, MXene/PDMS photothermal layer generates localized heat at the interface, facilitating effective solar‐driven separation. Under one‐sun illumination in direct‐contact operation, the system delivers a solar‐to‐thermal efficiency of 60%, a freshwater production rate of 1 kg m^−2^ h^−1^, and an ion rejection rate exceeding 99.95%. In addition, the membrane exhibits complete antibacterial behavior and maintains excellent separation (>99.95% flux and selectivity) when treating surfactant‐stabilized oil–water emulsions. These synergistic functionalities highlight the potential of this membrane architecture for sustainable solar desalination and advanced wastewater purification. Furthermore, incorporation of Ti_3_C_2_T_*x*_ NSs enhances photothermal conversion efficiency to 87.28% and enables high PEG loading up to 97.68 wt %. The resulting composite demonstrates strong thermal energy storage capability, with a latent heat exceeding 170 J g^−1^, along with excellent stability and retention. An evaporation rate of 1.24 kg m^−2^ h^−1^ and an efficiency of 50.6% under one‐sun irradiation were achieved, underscoring its promise for desalination applications [204]. Bin's team [205] developed a modular solar evaporator employing a flexible MXene aerogel with adjustable cellular and lamellar pore configurations for the desalination of oil‐polluted seawater (Figure 21c‐e). In this design, cellular pores enable effective oil rejection through combined multisieving effects and superhydrophobic behavior, while lamellar channels facilitate rapid vapor transport and evaporation. The evaporator achieved an evaporation rate of 1.48 kg m^−2^ h^−1^ and a solar‐thermal conversion efficiency of 92.08%, outperforming previously reported MXene‐based materials for oily seawater desalination.

In summary, vertically aligned MXene‐based hybrid aerogels exhibit outstanding performance in solar‐assisted desalination by optimizing capillary water transport and enhancing photothermal conversion efficiency. Their carefully engineered porous architectures effectively minimize thermal losses and suppress salt crystallization, enabling stable long‐term operation. As a result, these aerogels achieve high evaporation rates and solar‐to‐vapor conversion efficiencies under standard solar illumination, facilitating efficient freshwater generation. The combination of tunable surface wettability and hierarchical pore architectures positions MXene‐based composite aerogels as leading candidates for sustainable seawater desalination methods.

### 7.4 Sensor Application

MXene‐based aerogels provide a versatile sensing platform by combining great electrical conductivity with a compressible, hierarchically porous 3D framework and abundant surface functional groups. In pressure and strain sensing applications, the interconnected skeletal network forms contact‐dependent conductive routes, resulting in high gauge factors, minimal hysteresis, and rapid response times suitable for tactile electronic skins, motion monitoring, and human–computer interaction (HCI). For gas detection (e.g., NO_2_, NH_3_, and VOCs), as well as humidity and temperature sensing, the high accessible SSA and hydrophilicity of MXene aerogels promote rapid adsorption–desorption kinetics and efficient charge transport. Moreover, the incorporation of catalytic NPs, MOFs, or heteroatom dopants improves selectivity and reduces detection limits [208, 209, 210]. Their broadband optical absorption also supports optical and infrared sensing and photothermally amplified chemi‐resistive responses. For biological sensing, MXene aerogels can be functionalized with enzymes, aptamers, or antibodies to enable electrochemical detection of biomarkers (e.g., glucose, lactate, cortisol) and pathogenic species through impedance‐ or amperometry‐based signals, with interconnected pores facilitating rapid mass transport. Incorporation of elastomers, cellulose‐ or ionogel‐based matrices, graphene, or metal oxides enhances mechanical flexibility, antifouling properties, and environmental robustness, while the inherent EMI shielding capability reduces signal interference. When combined with low‐power electronics or triboelectric and piezoelectric energy‐harvesting units, these systems enable self‐powered, wearable, and multimodal sensing platforms [211, 212]. Selected examples of MXene aerogel sensors are briefed in Table 2.

**TABLE 2:** Key characteristics of MXene‐based 3D aerogel sensors.

Sensor classification	Aerogel fabrication	Signal	Application area	Ref.
Humidity sensor	Bilayer: Ti_3_C_2_T_*x*_ MXene aerogel + PAM	Moisture‐driven DC voltage/current (MEG output) modulated by bending/RH	Wearable self‐powered electronics; motion and facial‐expression sensing; wide‐env. operation	[213]
	Biomimetic MWCNT‐COOH/MXene/TOCNF aerogel; directional freeze‐dried, axially aligned pores + spray‐coated interface	Electrical resistance/impedance change vs RH, pressure (piezoresistive), and temperature	Wearables, e‐skin, environmental and physiological monitoring	[214]
	Ti_3_C_2_T_*x*_ MXene/chitosan/PVDF aerogel	Electrical impedance changes vs RH	Exhaled‐breath monitoring; nasal comfort control	[215]
Temperature sensor	PI/MXene hybrid aerogel	Thermoelectric	Smart fire protection, thermal insulation, robots, aerospace, rail transport	[216]
	Layered porous CNF/CNT/MXene hybrid aerogel		Wearable e‐skin, material recognition, thermoelectric energy harvesting	[217]
Gas sensor	3D MoS_2_/Ti_3_C_2_T_*x*_ aerogel (freeze‐dried VdW)	Resistance change	Air‐quality NO_2_ detection	[218]
	3D Ti_3_C_2_T_*x*_/rGO/CuO aerogel (CuO NP‐decorated)	Electrical resistance change	Breath/air‐quality acetone monitoring	[219]
	3D Ti_3_C_2_T_*x*_/rGO/SnO_2_ aerogel (one‐step solvothermal)	Electrical resistance changes upon HCHO exposure	Industrial and environmental formaldehyde monitoring	[220]
Heavy metal detection	3D melamine‐doped GO/MXene aerogel	Stripping‐voltammetry peak current	Water and cereal contamination monitoring	[221]
	MXene@rGO hybrid aerogel doped with UiO‐66‐NH_2_		Cd^2+^/Pb^2+^ monitoring in water and grains	[222]
	MXene aerogel‐CuO on C cloth with O‐vacancies + Bi(III)		Cd^2+^/Pb^2+^ monitoring in water and food	[223]
Electrochemical biosensor	3D Ti_3_C_2_T_*x*_/GO/EDA aerogel on C fiber paper + electrodeposited AuNPs	Electrochemical	miRNA diagnostics; clinical sample testing	[224]
	Ti_3_C_2_T_*x*_/rGO aerogel	Electrochemical current (amperometric)	Continuous glucose monitoring	[225]
	3D Au/MXene porous foam composite	Amperometric current	Blood glucose monitoring, biomedical diagnostics, food analysis	[226]
Pressure sensor	Superhydrophobic MXene‐coated C‐CNTs/CCS honeycomb aerogel (FAS‐modified)	Resistance changes vs pressure	Wearable e‐skin, robotics, health monitoring	[227]
	CNF/CNT/Ti_3_C_2_T_*x*_ hybrid aerogel with oriented tracheid‐like texture	Resistance change	Flexible/wearable energy storage	[123]
	MXene/aramid NF (ANF) hybrid aerogel film		Wearable motion and microexpression sensing	[228]
	GO/PVA/MXene hybrid aerogel		Physiological signals (fingertip pulse, temporal artery); harsh‐environment sensing	[117]
	Hollow MXene spheres/rGO hybrid aerogel		Real‐time human activity	[229]

**FIGURE 22:**
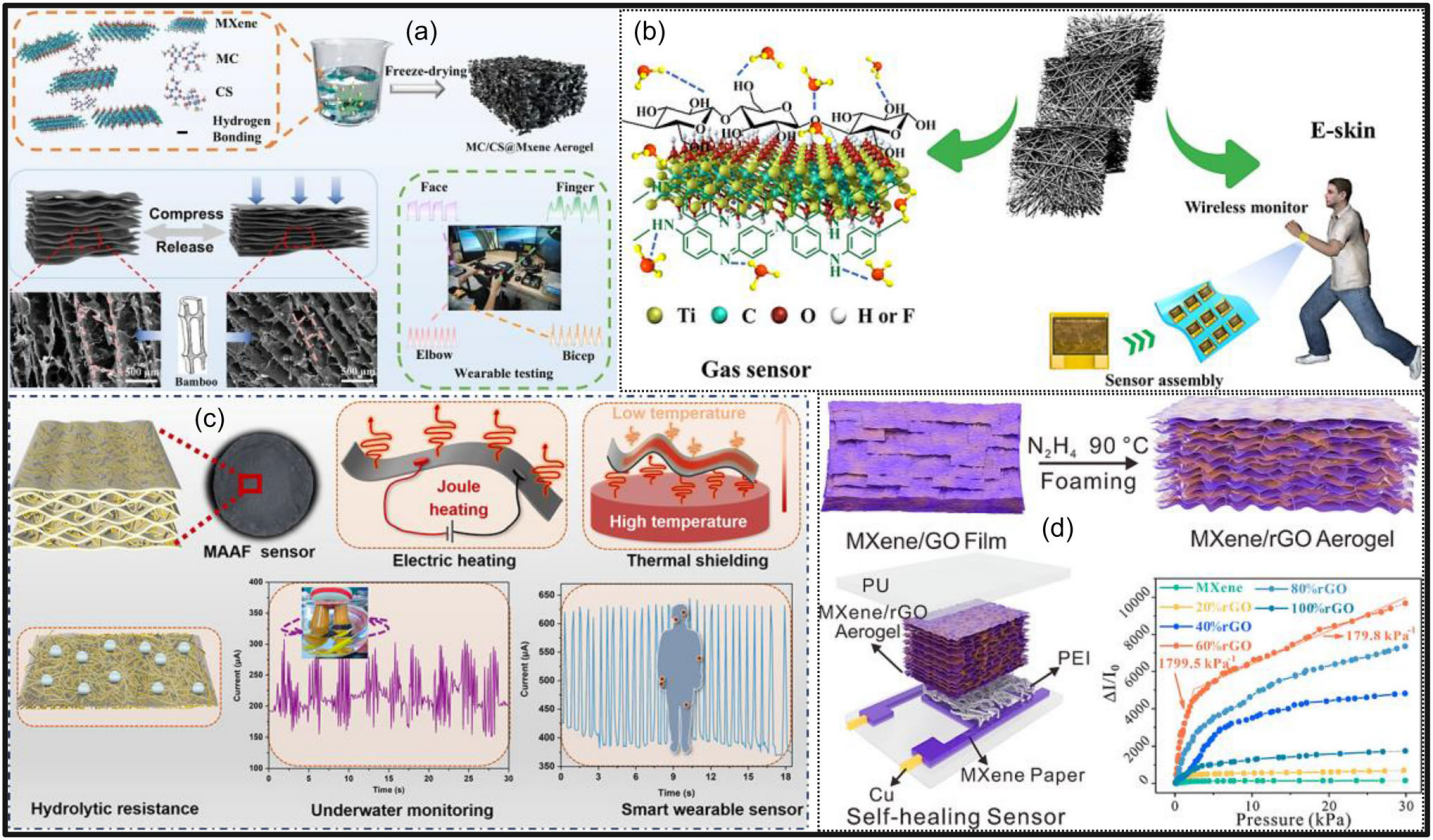
(a) Wearable methylcellulose/chitosan@MXene aerogel based piezoresistive sensor array with ultrahigh sensitivity for real‐time monitoring of pilots’ physiological signals. Adapted from [211]. Copyright 2025, Springer Nature. (b) Flexible Ti_3_C_2_T_*x*_/PANI/BC aerogel designed for electronic skin applications and gas sensing. Adapted from [230]. Copyright 2021, American chemical society. (c) Multifunctional pressure sensor based on a mechanically robust, hydrophobic MXene/ANF aerogel film. Adapted from [228]. Copyright 2022, American chemical society. (d) MXene aerogel with enhanced electron transport pathways enabling a flexible and self‐healable electronic skin. Adapted from [93]. Copyright 2023, American chemical society.

To overcome the shortcomings of traditional piezoresistive sensors, Zhao and coworkers fabricated an ultrasensitive, biocompatible MC/CS@MXene aerogel featuring a hollow, bamboo‐like microstructure via physical mixing followed by freeze‐drying (Figure 22a). The cooperative effects of electrostatic attraction and H‐bonding in the aerogel network produced a highly responsive resistive sensor with a sensitivity of 2.9 kPa^−1^, excellent durability exceeding 8000 compression cycles at 10 kPa, and fast response and recovery times of 119 and 91 ms, respectively. In addition, piezoresistive sensor arrays based on this aerogel exhibited considerable promise for wearable electronics, HCIs, and real‐time physiological monitoring of civil aviation pilots, highlighting a viable strategy for reliable, high‐performance sensing systems [211].

MXene/polyaniline (PANI)/BC aerogels fabricated through the self‐assembly of MXene NSs with 1D functional components have employed as active sensing layers in electronic‐skin pressure sensors. These devices exhibit sufficient sensitivity to detect subtle mechanical stimuli, including finger and wrist bending as well as pulse signals (Figure 22b). When combined with few‐layer or single‐layer MXenes, the aerogel‐based sensors enable Bluetooth‐assisted wireless transmission, allowing real‐time visualization of spatial pressure distributions on mobile platforms and demonstrating their suitability for electronic‐skin applications. In addition, the 3D porous framework and surface terminations of MXene facilitate efficient electron transport and gas adsorption, enabling NH_3_ sensing capability [230].

Bin and coworkers [228] reported an ultralight MXene/ANF aerogel film featuring a 3D porous architecture that combines great mechanical robustness, flexibility, and electrical conductivity (Figure 22c). With a MXene content of 30 wt %, the aerogel film withstood tensile stresses up to 14 MPa while maintaining a fast electromechanical behavior and a high gauge factor of 37 k Pa^−1^, enabling motion detection with a temporal resolution of ∼100 ms for joint movements and facial micro‐expressions. In addition, the conductive network functioned as an electrothermal layer capable of reaching temperatures from 32 to 242°C within 5 s, while the intrinsic porous structure provided effective thermal insulation against external heat transfer. A hydrophobic SiO_2_ surface coating further allowed stable sensing performance in aqueous environments, enabling accurate monitoring of swimming motions. In a related approach, Yongfa's group [93] employed a gas‐foaming strategy to enlarge interlayer spacing of MXene, producing an interlayer‐tunable aerogel with expanded accessible SSA and abundant electron transport pathways. The resulting self‐healable force sensor exhibited high sensitivity (1.8 × 10^3^ kPa^−1^), fast response (11 ms), and excellent durability over more than 25 000 loading cycles, demonstrating strong potential for subtle pressure sensing, tactile HCIs, and remote physiological monitoring (Figure 22d).

**FIGURE 23:**
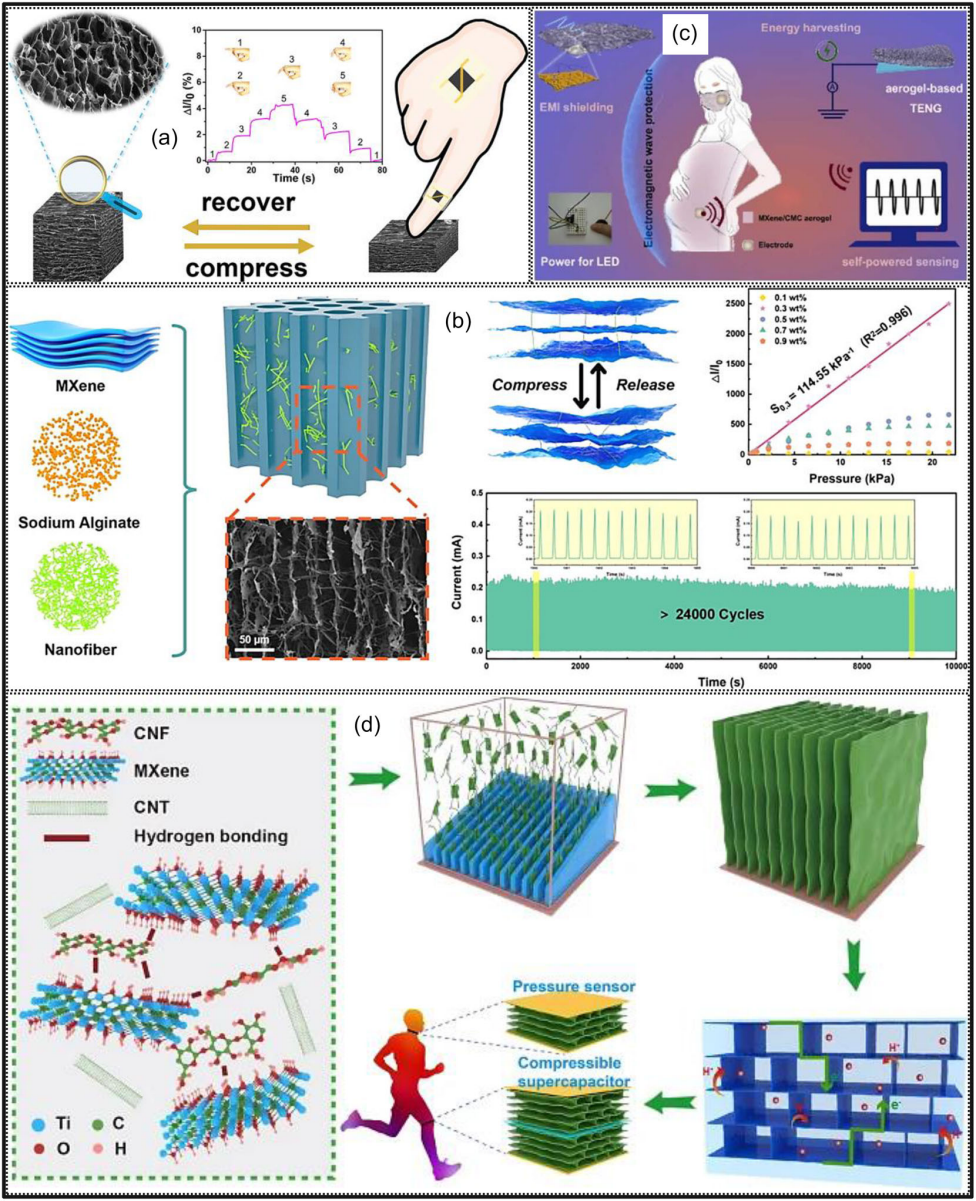
(a) PI‐reinforced graphene/3D MXene aerogels used as ultralight, high‐sensitivity piezoresistive frameworks for advanced pressure sensing. Adapted from [127]. Copyright 2022, American chemical society. (b) High‐performance wearable piezoresistive sensor constructed from NF/SA enhanced MXene aerogels. Adapted from [231]. Copyright 2023, Elsevier B.V. (c) Flexible MXene/CMC aerogels for EMI shielding, triboelectric energy harvesting, and self‐powered sensing. Adapted from [232]. Copyright 2022, Elsevier B.V. (d) Wood‐inspired MXene‐cellulose aerogels functioning as compressible electrodes with integrated pressure‐sensing capability. Adapted from [123]. Copyright 2023, Springer Nature.

MXene/ANF aerogel films fabricated via vacuum‐assisted filtration combined with ice‐crystal‐induced assembly exhibit excellent multifunctional sensing performance. These films deliver great tensile strength (14.1 MPa), elastic modulus (455 MPa), and electrical conductivity, along with a pressure sensitivity of 37.4 k Pa^−1^ and a fast response time of 100 ms for detecting human motion and subtle facial expressions. Introduction of a hydrophobic SiO_2_ coating further enhances underwater sensing capability, enabling applications such as swimming monitoring. In addition, the aerogel films demonstrate outstanding thermal insulation and rapid electrothermal response (32.7–242°C within 5 s), extending its utility to wearable sensors, healthcare systems, intelligent robotics, and underwater sensing platforms (Figure 23a) [127]. Separately, a lightweight cellulose acetate NF/SA‐modified MXene hybrid aerogel (17.33 mg cm^−3^), produced through liquid‐N_2_‐assisted unidirectional freezing, exhibits high compressive strength (16 kPa), ultrahigh sensitivity (114.55 k Pa^−1^) over a wide pressure range (up to 21.78 kPa), and excellent durability exceeding 24 000 compression cycles (Figure 23b). Its wireless functionality enables real‐time monitoring of human behaviors, making it well suited for wearable applications [231].

MXene/carboxymethyl cellulose (MXene/CMC) aerogels function as triboelectric nanogenerators and self‐powered sensors while simultaneously providing effective EMI shielding, with shielding efficiencies (SE) of 52.15 dB in the X‐band, 60.31 dB in the Ku‐band, and 80.36 dB in the K‐band (Figure 23c). These TENG devices deliver a peak‐to‐peak V_OC_ of 54.37 V, a I_SC_ of 1.22 μA, and a power density of 402.94 mW m^−2^, sufficient to drive commercial light‐emitting diodes. When attached to the human body, they enable real‐time health monitoring while simultaneously offering protection against EM radiation, thus integrating energy harvesting and health‐protection functions within a single platform [232]. In a separate study, Huo's team [221] reported a 3D melamine‐doped GO/MXene aerogel integrated into a screen‐printed C electrode for the electrochemical detection of Zn^2+^, Cd^2+^, and Pb^2+^ ions. The self‐assembled 3D architecture enhances electrical conductivity and ion adsorption, allowing sensitive detection over a broad concentration range. This sensing platform was successfully applied to water and cereal examples, demonstrating strong potential for environmental and agricultural monitoring of heavy metal pollution.

Dong and coworkers [223] reported an electrochemical sensor based on an MXene aerogel‐CuO/C cloth (MXA‐CuO/CC) composite for the detection of Cd^2+^ and Pb^2+^ ions. Enhanced ion adsorption was achieved through the presence of O‐vacancies in CuO and alloy formation facilitated by Bi(III). Using differential pulse anodic stripping voltammetry (DPASV), the sensor exhibited great sensitivity, simultaneous detection capability, and strong resistance to interference, along with excellent stability and reproducibility. Its analytical performance was validated by comparison with inductively coupled plasma mass spectrometry (ICP‐MS) and atomic absorption spectroscopy (AAS), demonstrating its reliability for monitoring Cd^2+^ and Pb^2+^ in food and water samples relevant to environmental and food safety applications.

Zhang's team [123] fabricated a CNF/CNT/MXene aerogel by intertwining CNFs and CNTs around MXene NSs through a bidirectional freezing approach, producing wood‐inspired tracheid‐like channels within the framework. The aerogel exhibits an ultralow density of 7.5 mg cm^−3^, maintains structural integrity under 80% compressive strain for over 1000 cycles, and displays an electrical conductivity of approximately 2.4 × 10^−3^ S m^−1^. Owing to its high linear pressure sensitivity (817 kPa^−1^), the aerogel converts mechanical stimuli into pronounced resistance changes, enabling accurate monitoring of human motion (Figure 23d). When compressed into solid‐state SCs, the same architecture delivers an areal capacitance of 849 mF cm^−2^ at 0.8 mA cm^−2^ and retains 88% of its capacitance after 10 000 compression cycles, demonstrating excellent electrode resilience and integrated pressure‐sensing functionality. Collectively, these results highlight MXene aerogels as promising candidates for advanced sensing systems that require high sensitivity and mechanical compliance across capacitive, chemical, and flexible sensing platforms.

### 7.5 Biomedical Field

MXene‐based hybrids, especially aerogels and hydrogels, provide flexible and electrically conductive 3D frameworks rich in surface functional groups, allowing precise chemical functionalization and responsive behavior to external stimuli. These platforms enable diverse biomedical applications, including targeted drug delivery, near‐infrared (NIR) photothermal therapy, antibacterial treatments, rapid hemostasis, tissue repair, and extracorporeal blood purification [41, 233]. Their hierarchical pore structures promote effective mass transport and cell migration, while efficient photothermal conversion supports controlled therapeutic release and wound disinfection, thereby inhibiting biofilm development. Together, these attributes facilitate the design of multifunctional implants, smart wound dressings, and regenerative scaffolds that offer localized, adaptable, and efficient alternatives to traditional therapies.

Surface modification strategies, such as PEGylation and the grafting of peptides or aptamers, can significantly improve the biocompatibility and targeting capability of MXene‐based materials while regulating their interactions with the immune system. The integration of antioxidants or protective polymer coatings enhances resistance to oxidative degradation and sterilization processes without compromising electrical conductivity. Furthermore, the incorporation of imaging agents, including photoacoustic, fluorescent, or magnetic resonance probes (MRIs), enables the development of theranostic platforms capable of real‐time monitoring of material distribution and therapeutic outcomes. In addition, additive manufacturing approaches using MXene‐containing inks facilitate the fabrication of patient‐specific constructs with tailored mechanical properties and degradation behaviors to meet precise clinical requirements [210, 233, 234].

**FIGURE 24:**
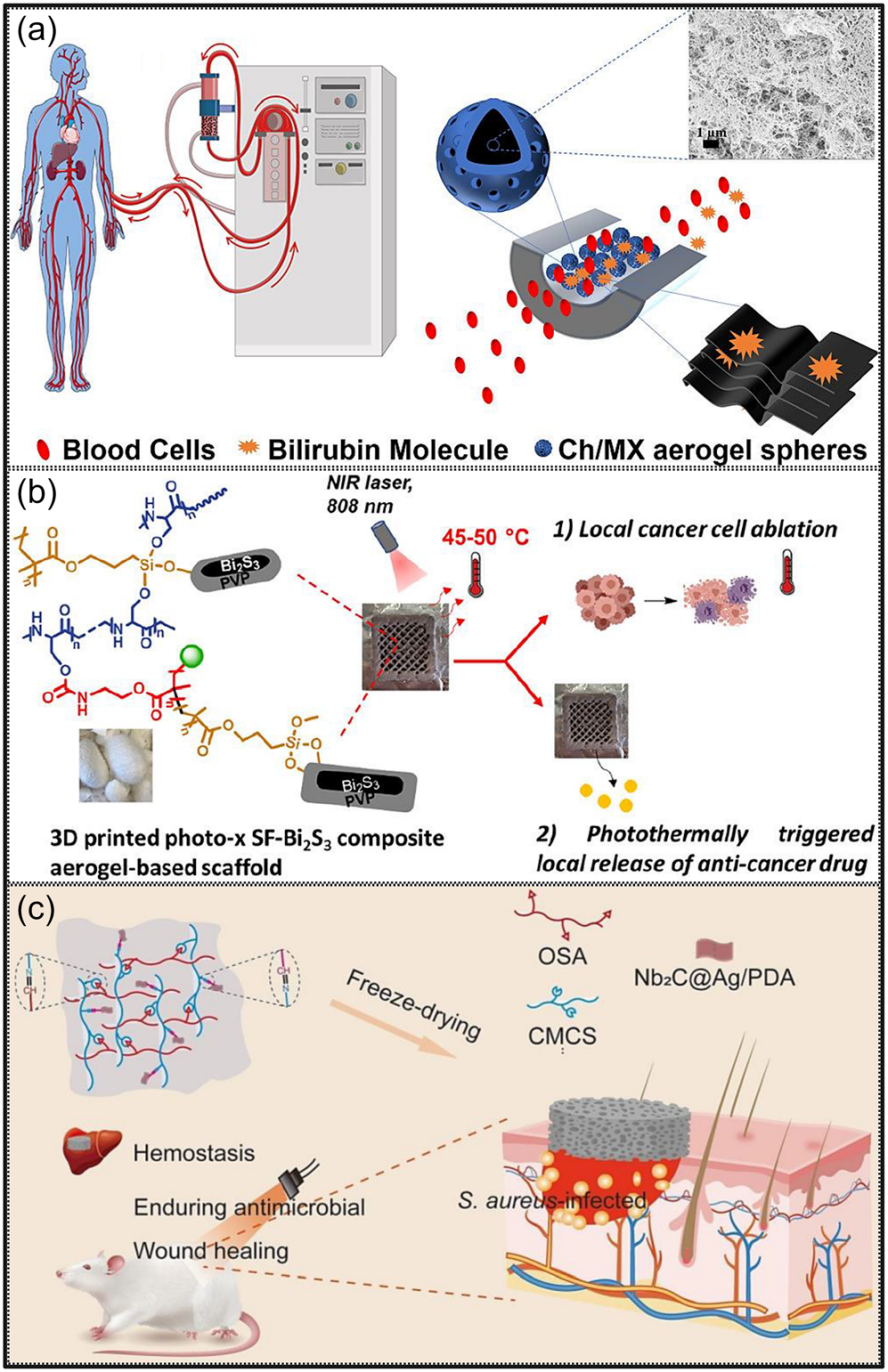
(a) MXene‐enhanced chitin‐based aerogel microspheres designed for efficient bilirubin adsorption. Adapted from [235]. Copyright 2022, American chemical society. (b) Self‐assembled Bi_2_S_3_ nanobelt integrated silk fibroin 3D‐printed aerogel framework for photothermal bone cancer treatment. Adapted from [236]. Copyright 2023, American chemical society. (c) MXene‐based multifunctional polysaccharide aerogel exhibiting prolonged antibacterial activity for infected wound healing. Adapted from [237]. Copyright 2024, Elsevier B.V.

Guiyin and colleagues [235] developed chitin/MXene hybrid aerogel microspheres capable of efficiently removing bilirubin from blood, thereby mitigating potential damage to the brain and nervous system (Figure 24a). Incorporation of single layer Ti_3_C_2_ NSs significantly enhanced both the mechanical robustness and adsorption performance of aerogels, enabling them to support loads up to 25 000 times their own mass. Compared with pristine chitin aerogels, the composites exhibited a 33% higher adsorption rate and a 40% increase in bilirubin uptake. Notably, the aerogels maintained a high adsorption capacity (142.86 mg g^−1^) even in the presence of high concentrations of competing proteins like bovine serum albumin. Overall, these MXene‐containing aerogels outperformed profitable clinical adsorbents and previously reported chitin/graphene hybrids in terms of bilirubin removal efficiency, selectivity, and biocompatibility, highlighting their promise for hyperbilirubinemia treatment. In a separate study, a silk fibroin‐based 3D‐printed framework incorporating Bi_2_S_3_ belts and MXene demonstrated photothermally triggered drug release, effective tumor ablation, and simultaneous bone tissue regeneration, underscoring its potential as an implantable scaffold for bone tissue engineering (Figure 24b) [236]. Li et al. [237]. fabricated a Schiff base‐crosslinked composite aerogel composed of SA oxide, CMC, and Nb_2_C@Ag/PDA for enhanced wound repair and infection control (Figure 24c). The resulting aerogel exhibited high swelling capacity, interconnected porosity, favorable biocompatibility, and long‐lasting antibacterial activity. Notably, antibacterial inhibition against *Staphylococcus aureus* and *Escherichia coli* remained close to 100% even after 25 hr of NIR laser irradiation. In vivo wound‐healing studies demonstrated nearly complete closure within 14 days, markedly outperforming untreated control groups. Moreover, both in vitro and in vivo assessments validated the strong hemostatic capability of aerogel, underscoring its promise as a multifunctional dressing for infected and chronic wounds.

**FIGURE 25:**
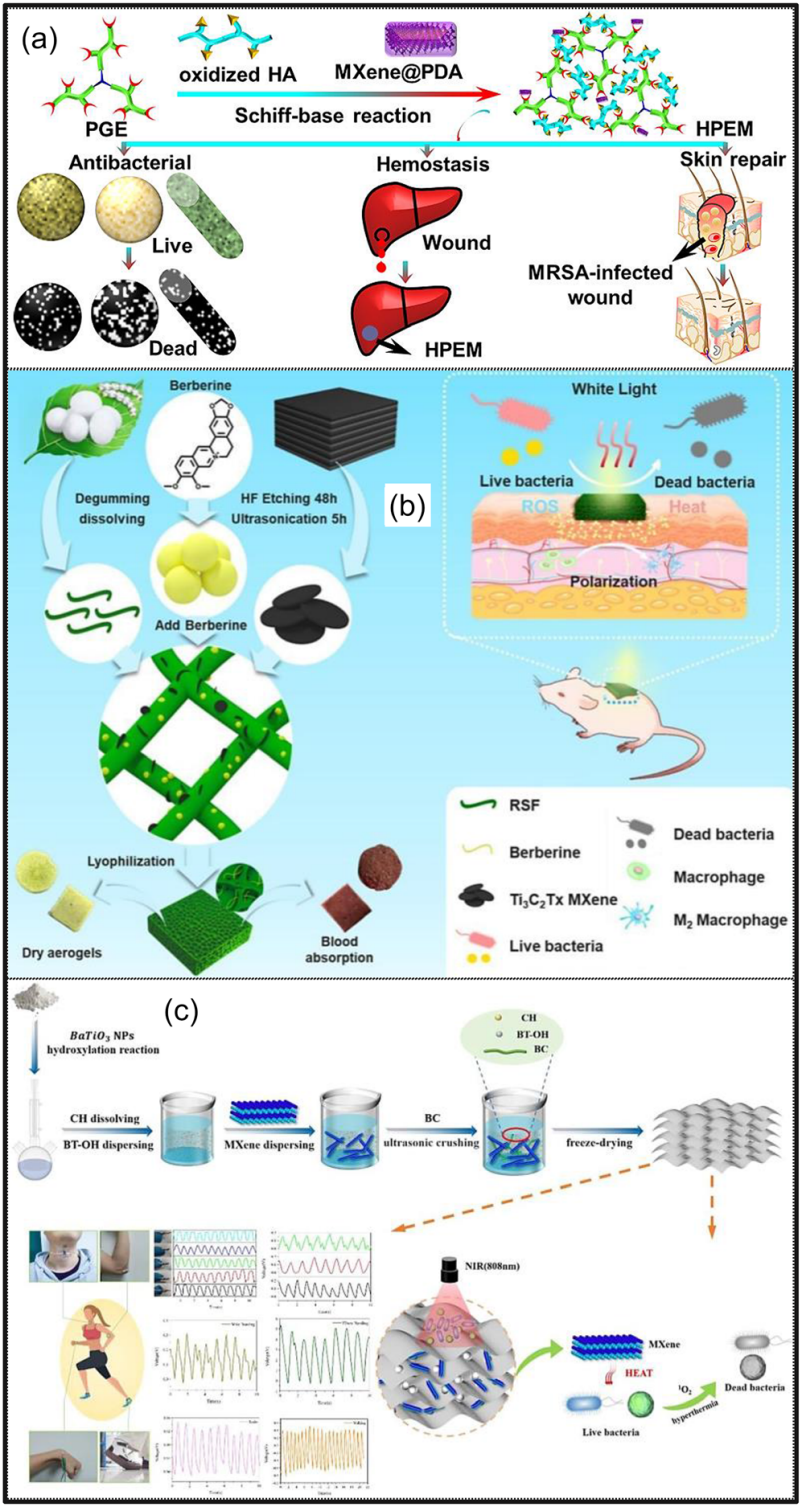
(a) MXene NSs based conductive frameworks designed for antibacterial, hemostatic, and infected‐wound healing applications. Adapted from [238]. Copyright 2021, American chemical society. (b) AIE‐enabled silk fibroin/MXene aerogels containing berberine for fast blood coagulation and effective wound healing. Adapted from [239]. Copyright 2024, Elsevier B.V. (c) MXene‐BaTiO_3_ NPs reinforced biomass aerogels exhibiting photothermal antibacterial activity and ultrasensitive self‐powered sensing capability. Adapted from [240]. Copyright 2023, Elsevier B.V.

Multifunctional frameworks constructed from poly(glycerol‐ethylenimine), MXene@PDA NSs, and oxidized hyaluronic acid exhibit self‐healing capability, strong adhesion to biological tissues, and effective antibacterial activity against multidrug‐resistant strains such as MRSA in wound‐healing applications (Figure 25a). These properties contribute to accelerated wound closure, enhanced angiogenesis, and increased collagen formation, underscoring their promise for treating infected wounds [238]. In a related advance, a DNA‐inspired hydrogel formed by crosslinking MXene with short‐stranded DNA (smDNA) was reported to enable the codelivery of Adriamycin and the tumor‐suppressive microRNA (miR‐375). The system demonstrates pH‐responsive release behavior, selectively activating in the acidic tumor microenvironment, thereby achieving localized tumor inhibition with minimal systemic toxicity and offering potential for post‐surgical tissue regeneration [241].

Likewise, a silk fibroin aerogel incorporating aggregation‐induced emission (AIE)‐modified berberine and MXene has been reported to enable rapid blood coagulation and strong antibacterial performance through synergistic photothermal and photodynamic mechanisms (Figure 25b). The material exhibits excellent hemocompatibility and significantly accelerates healing in infected wounds, highlighting its potential for emergency clinical applications [239]. In a related study, a biomass‐derived chitosan/BC aerogel integrated with hydroxylated BaTiO_3_ NPs and MXene demonstrated high piezoelectric responsiveness for motion detection, alongside robust photothermal antibacterial activity. These multifunctional features make the aerogel particularly attractive for wearable biosensors that combine real‐time sensing with infection control (Figure 25c) [240].

Beyond wound‐healing applications, MXene‐based waterborne PU aerogels have been developed as phase‐change composite materials with light‐responsive shape‐memory and self‐repairing functions, enabling multifunctional performance in solar‐thermal energy harvesting and biomedical thermal regulation [242]. In addition, chitin/MXene/poly(L‐arginine) hybrid aerogel microspheres have been explored as hemoperfusion adsorbents, demonstrating high bilirubin uptake, strong selectivity, excellent blood compatibility, and robust structural integrity, indicating their potential for clinical management of hyperbilirubinemia and other toxin‐associated disorders [243]. Wu and colleagues [244] developed a chitosan/PVA aerogel (CPMAE) for berry preservation that enables the sustained release of MXene‐supported Ag NPs and trans‐2‐hexenal. The porous aerogel structure effectively dissipates mechanical stress, while only limited amounts of Ag (∼6%) and E_2_H (∼67%) are gradually released over 1 week, which is sufficient to achieve strong antimicrobial performance (∼99%) along with notable antioxidant activity. Owing to its ultralight nature, CPMAE padding preserved strawberry firmness and suppressed mold growth, with fruit remaining fresh after 12 days of storage at 4°C, demonstrating its dual functionality as both an impact‐buffering material and an active preservative.

Xing's team [245] developed a breathable smart textile by coating cellulose‐based nonwoven fabrics with Ti_3_C_2_T_*x*_ layers. The MXene coating undergoes reversible expansion and contraction in response to ambient humidity, enabling the conversion of respiratory moisture signals into distinct resistance variations for accurate breathing monitoring. Owing to its metallic conductivity, the fabric rapidly generates heat under low applied voltage, allowing it to function as a safe thermotherapy platform equipped with a humidity‐assisted temperature alarm. In addition, the Joule‐heating effect provides effective antibacterial activity, enabling wound sanitization. This lightweight and flexible textile integrates respiration sensing, adaptive heating, and antimicrobial functionality, highlighting its promise for next‐generation wearable healthcare systems. Chao and coworkers [246] fabricated a fully breathable and biodegradable pressure sensor by depositing Ti_3_C_2_T_*x*_ onto silk fibroin NFs membranes and subsequently ink‐printing interdigitated MXene electrodes. The resulting MXene/SF sensing network operates across a broad pressure range (0–39 kPa) and delivers high piezoresistive sensitivity, reaching 298 kPa^−1^ at pressures below ∼ 16 kPa and 172 kPa^−1^ at higher loads. The device exhibits a rapid response time of 7 ms and maintains stable performance over 10 000 loading‐unloading cycles without hindering skin breathability. When mounted on the human body, it enables real‐time monitoring of physiological signals, including pulse detection, limb motion, spatial pressure distribution, and wireless data transmission, offering an environmentally benign alternative to conventional plastic‐based electronic skins.

**FIGURE 26:**
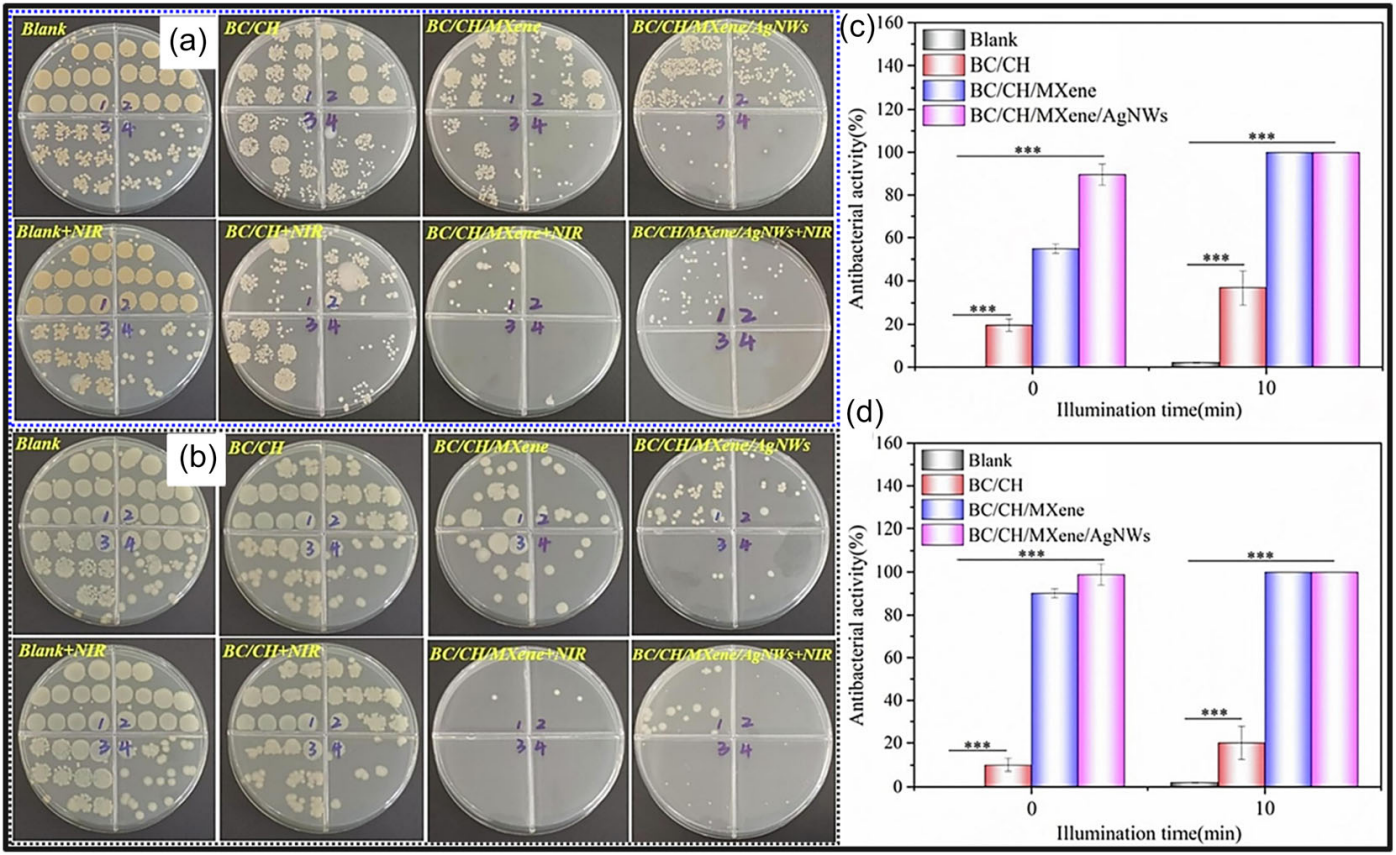
Agar plate images of bacterial colonies after 24 hr incubation: (a) *S. aureus* and (b) *E. coli*. The four plate quadrants correspond to serial dilution factors of 10^1^, 10^3^, 10^4^, and 10^6^. Antibacterial performance of various composite aerogels under dark and NIR irradiation against (c) *S. aureus* and (d) *E. coli*. Adapted from [247]. Copyright 2022, Elsevier B.V.

Yijun's group [247] examined the antibacterial performance of BC/chitosan (BC/CH) hybrid aerogels incorporated with MXene and Ag NWs, as depicted in Figure 26. Agar plate assays revealed pronounced differences in *S. aureus* viability following treatment with various aerogels under both dark conditions and NIR irradiation (Figure 26a). The untreated control groups (quadrants 1–4) showed dense bacterial colonies and biofilm‐like coverage, whereas the BC/CH aerogel markedly suppressed bacterial growth, mostly after 10 min of NIR exposure, demonstrating a photothermal antibacterial effect. Incorporation of MXene further enhanced bacterial inhibition (Figure 26b), reflecting the combined intrinsic antibacterial action and improved photothermal conversion. The BC/CH/MXene/AgNW aerogel achieved strongest antibacterial response due to the synergistic effects of MXene and Ag NWs. Quantitative analysis (Figure 26c) confirmed the progressive enhancement in antibacterial efficiency with MXene and AgNW loading and NIR irradiation. Comparable antibacterial trends were observed against *E. coli* (Figure 26b,d), with the hybrid aerogel again exhibiting superior performance.

In summary, recent advances highlight the remarkable promise of MXene‐integrated hydrogels and aerogels for next‐generation biomedical technologies. These compounds exhibit a broad spectrum of functions, including localized cancer therapy, infection control, rapid blood coagulation, enhanced wound repair, toxin removal, and intelligent sensing, while maintaining excellent biocompatibility and mechanical adaptability. By combining controlled drug delivery, antimicrobial performance, and photothermal treatment within a single construct, MXene‐based aerogel composites enable highly integrated and customizable therapeutic platforms. Nonetheless, widespread clinical implementation will depend on progress in scalable manufacturing, rigorous long‐term biosafety evaluation, and successful clinical translation.

### 7.6 MW Absorption

MXene‐based aerogels exhibit outstanding MW absorption performance owing to their ultralight, hierarchically porous 3D frameworks, which combine strong dielectric attenuation with favorable impedance matching. The interconnected but highly tortuous conductive networks generate significant conduction losses and Joule heating, while numerous defects, surface terminations, and heterogeneous interfaces (e.g., MXene‐air, MXene‐polymer, and MXene‐inorganic phases) promote dipolar and interfacial polarization, thereby dissipating incident EM waves as thermal energy rather than reflecting them [248, 249].

**FIGURE 27:**
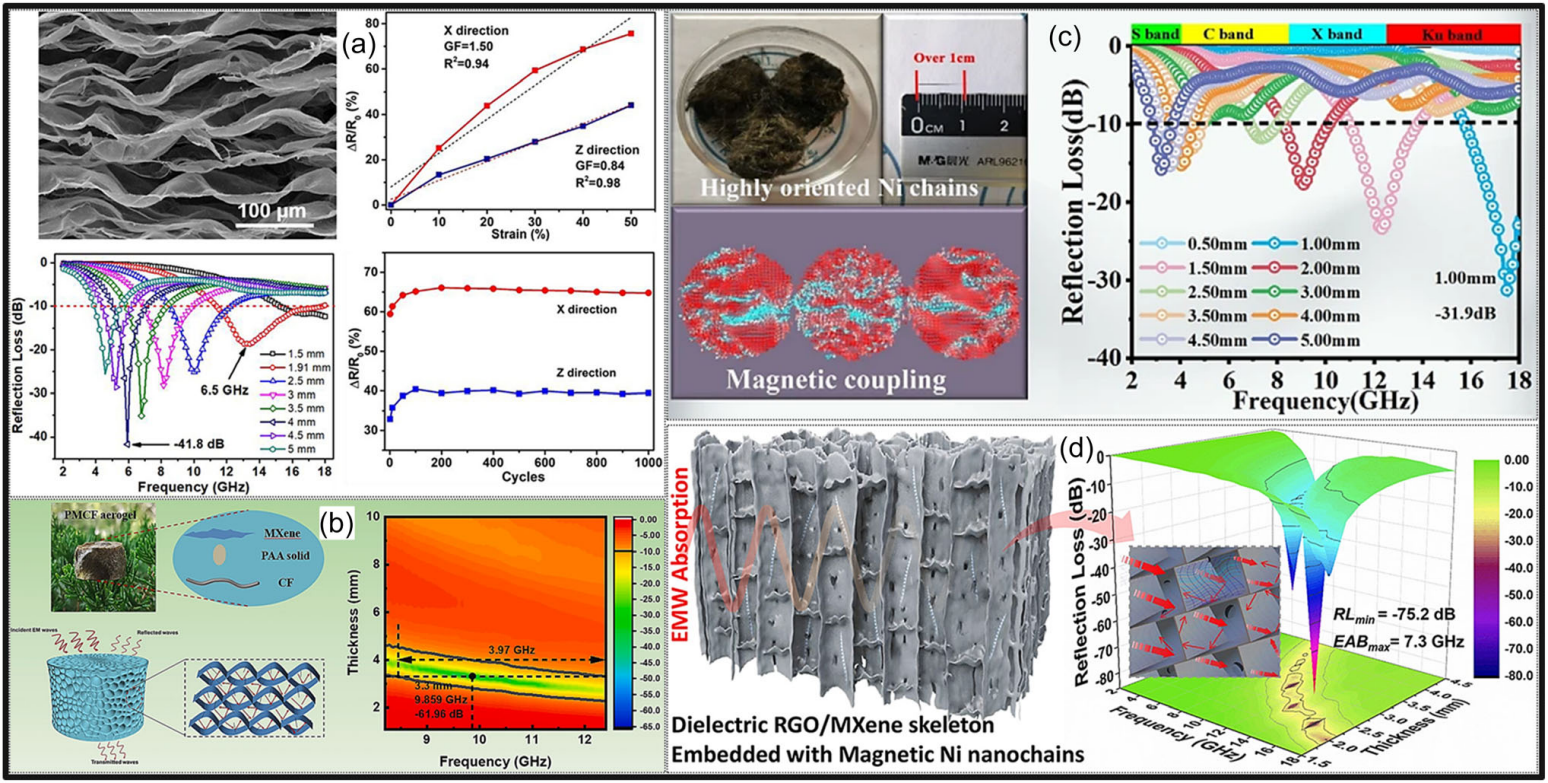
(a) Anisotropic MXene aerogels exhibiting high sensitivity, mechanical robustness, and broadband MW attenuation. Adapted from [254]. Copyright 2020, Elsevier B.V. (b) Multifunctional hybrid aerogel composed of MXene, CNFs, and PI for efficient MW absorption. Adapted from [253]. Copyright 2024, Elsevier B.V. (c) MXene/Ni‐chain aerogel absorber achieving ultrathin matching thickness through macroscopic EM synergy. Adapted from [255]. Copyright 2022, Springer Nature. (d) Magnetic Ti_3_C_2_T_*x*_/graphene aerogel demonstrating exceptional EM wave absorption performance. Adapted from [256]. Copyright 2021, American chemical society.

The porous framework functions as a graded refractive layer that mitigates interfacial reflection, induces repeated internal scattering, and extends the EM propagation distance, thereby strengthening overall attenuation. By adjusting the aerogel thickness and pore dimensions, the structure can be engineered to satisfy the quarter‐wavelength matching condition within targeted frequency ranges, leading to enhanced destructive interference and reduced reflection loss [250]. Incorporation of magnetic NPs or ferrite phases into MXene aerogels introduces coupled dielectric‐magnetic loss mechanisms, improving impedance matching and contributing additional magnetic dissipation (e.g., natural and resonance losses), which effectively widens the absorption bandwidth. In parallel, the intrinsic compressibility and ultralow mass density allow the aerogels to form lightweight, conformal coatings on nonplanar substrates, while polymeric or hybrid encapsulation strategies protect MXenes from oxidative degradation without compromising EM behavior [248, 251]. Typically fabricated via advanced routes like bidirectional freezing, lyophilization, or hydrothermal assembly, these aerogels achieve extremely low densities in the range of 5.2–18.58 mg cm^−3^, making them well suited for mass‐sensitive applications including wearable electronics, stealth systems, and communication technologies [252, 253]. Yang and coworkers [254] developed lightweight, compressible anisotropic MXene/PI aerogels for multifunctional applications in MW attenuation and pressure sensing (Figure 27a). The bidirectional freezing strategy produced a wave‐like lamellar architecture that enhanced mechanical robustness and electrical transport along the aligned layers while maintaining high elasticity in the transverse direction. Owing to this anisotropic framework, the aerogels exhibited stable, direction‐dependent piezoresistive responses over 1000 compression cycles at strains up to 50%. Structural optimization enabled precise tuning of MW absorption behavior, achieving an exceptionally broad effective absorption bandwidth (EAB) of 6.5 GHz with a minimal thickness of 1.91 mm, among the highest performances reported for MXene‐based absorbers. These features highlight the promise of such aerogels as wearable pressure sensors and high‐efficiency EMI shielding materials, covering the entire X‐band at a thickness of 2.57 mm.

Yan et al. [253]. reported the fabrication of hydrophobic Ti_3_C_2_T_*x*_/CNFs/PI aerogels that combine ultralight weight with outstanding mechanical robustness and fire resistance (Figure 27b). These aerogels exhibited a high compressive strength of 683.33 kPa and maintained structural integrity at temperatures approaching 500°C. Their porous framework enabled an extremely low density of 18.58 mg cm^−3^ while delivering outstanding MW attenuation, achieving a lowest reflection loss of −61.96 dB and an EAB of 3.97 GHz. Synergistic integration of MXene, CNFs, and PI enhanced dielectric loss mechanisms, resulting in efficient EM wave dissipation. Owing to this combination of mechanical durability, thermal stability, and EM performance, these aerogels show strong promise for stealth and electronic systems operating under harsh conditions. Lu and colleagues [255] developed a magnetic aerogel inspired by the structure of *Ficus microcarpa*, featuring centimeter‐scale, macroscopically aligned Ni chains formed through magnetic field‐assisted assembly (Figure 27c). The ordered Ni framework establishes strong magnetic coupling and continuous EM conduction pathways. As a result, the aerogel exhibits enhanced EM parameters at high frequencies, effectively overcoming the thickness limitations commonly associated with traditional dielectric‐magnetic absorbers. Owing to the optimized cooperative EM network, efficient MW absorption is achieved at an ultrathin thickness of only 1 mm. This study introduces a viable strategy for realizing ultrathin EM absorbers by leveraging macroscopic structural engineering and dielectric‐magnetic synergy. Xie's team [256] reported an ultralight Ni/MXene/rGO aerogel with a density of only 6.45 mg cm^−3^ and a directionally organized cellular architecture (Figure 27d). This material demonstrates unprecedented EM wave absorption among MXene‐based absorbers, with a minimum reflection loss of −75.2 dB, corresponding to nearly complete attenuation of incident waves, and an ultra‐broad EAB of 7.3 GHz. These superior properties arise from well‐balanced impedance matching and strong coupling between dielectric and magnetic loss mechanisms. The 3D framework integrates the dielectric contribution of MXene with the magnetic behavior of Ni nanochains, generating multiple polarization pathways that enhance attenuation. Moreover, the aerogel combines high mechanical robustness, hydrophobicity, and thermal insulation, enabling stable operation under deformation and in demanding environmental conditions.

**FIGURE 28:**
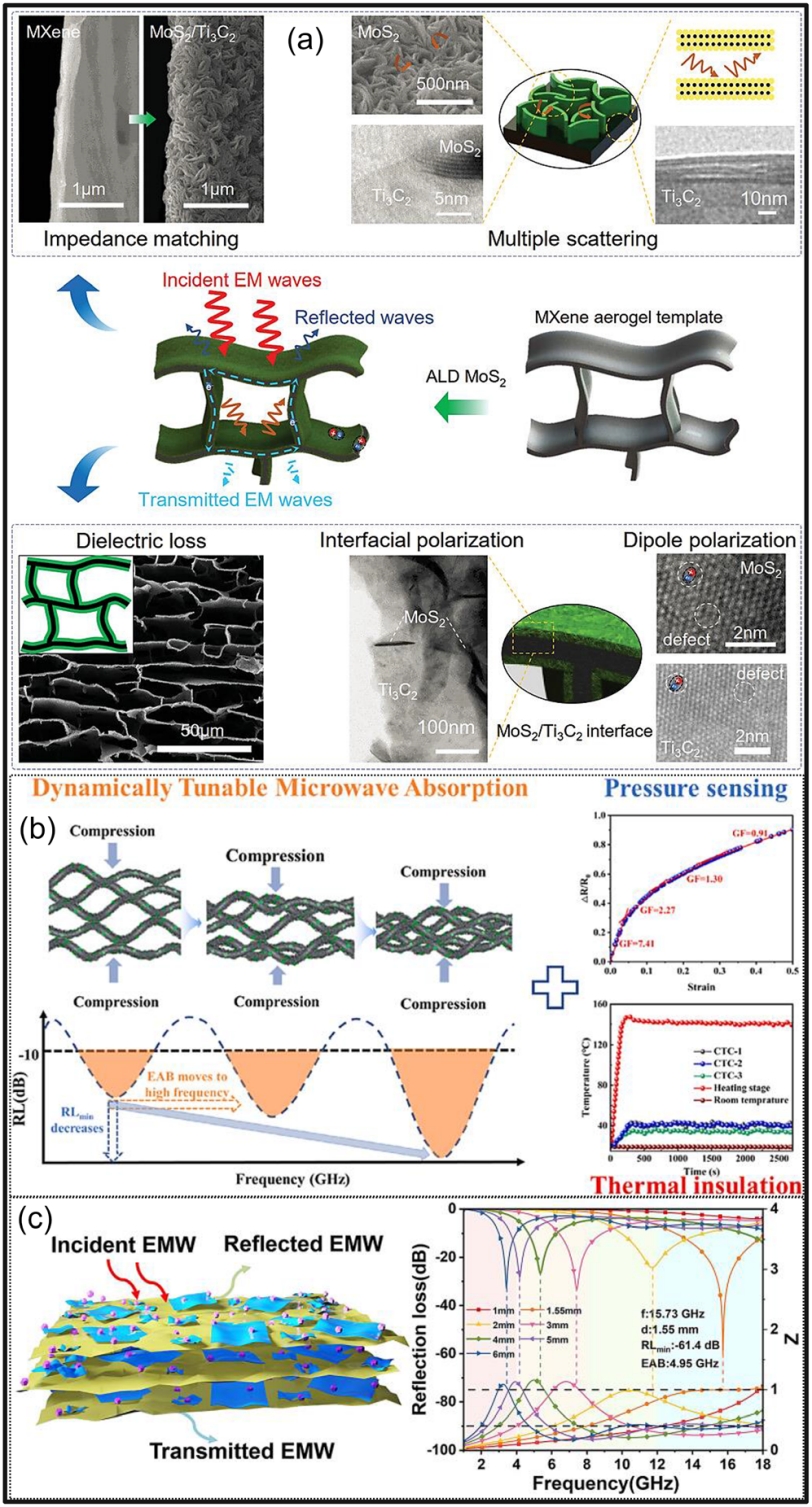
(a) MoS_2_/MXene aerogel featuring conformal heterogeneous interfaces engineered via ALD for adjustable MW absorption behavior. Adapted from [257]. Copyright 2022, Wiley‐VCH. (b) Pressure‐responsive multifunctional CNFs@TiO_2_/C aerogels derived from MXene for tunable MW absorption. Adapted from [258]. Copyright 2024, Elsevier B.V. (c) MOF‐assisted rGO/MXene/FeCoC aerogels demonstrating high‐efficiency MW absorption. Adapted from [259]. Copyright 2024, American chemical society.

Lei and coworkers [257] reported the preparation of a 3D porous MoS_2_/MXene composite aerogel via atomic layer deposition (ALD), in which interfacial design was used to enhance MW absorption performance (Figure 28a). The preserved MXene porous framework promotes dielectric loss by increasing EM wave scattering, while the conformal MoS_2_ layer shifts the EM response from reflection‐dominated shielding to absorption by improving impedance matching. By adjusting the number of ALD cycles, an optimal balance between impedance matching and attenuation was achieved, with the 300‐cycle sample exhibiting the strongest absorption (minimum reflection loss of −61.65 dB at 4.53 mm thickness). In addition, the composite aerogel maintains favorable mechanical strength, hydrophobicity, and low density, supporting its applicability in demanding EM environments.

In a related work, Meng et al. [260]. developed an ultralight MXene/cellulose aerogel via directional freeze‐drying that integrates both MW and acoustic attenuation capabilities. The resulting biomimetic honeycomb architecture delivered strong MW absorption in the X‐band, achieving a minimum reflection loss of −44.9 dB, alongside an excellent sound absorption coefficient of 0.82 across the 1000–6300 Hz frequency range. Precise regulation of MXene loading and aerogel density enabled simultaneous optimization of EM and acoustic energy dissipation within a single sustainable platform. This study highlights the effectiveness of bioinspired porous architectures for multifunctional pollution control and provides a design strategy for next‐generation materials capable of addressing EMI and noise pollution concurrently. Lu's team [261] reported a bioinspired hollow MXene aerogel fiber that demonstrates outstanding MW attenuation, achieving a lowest reflection loss of −52.39 dB. The hierarchical hollow‐porous architecture promotes repeated internal scattering and strong dielectric dissipation, leading to highly efficient EM energy attenuation. This fiber‐based structural concept opens new pathways for developing high‐performance MW absorbers while preserving mechanical integrity.

Sun's group [258] reported the fabrication of pressure‐sensitive CF@TiO_2_/C aerogels exhibiting mechanically adjustable MW absorption behavior, achieving an ultrabroad effective bandwidth of 7.84 GHz and a minimum reflection loss of −74.38 dB under applied compression (Figure 28b). The MXene‐derived 2D TiO_2_/C composite architecture enables real‐time modulation of absorption characteristics across the C‐, X‐, and Ku‐frequency bands through mechanical deformation. In addition to strong EM attenuation, these aerogels possess favorable mechanical robustness and thermal insulation performance. This work demonstrates a promising strategy for the development of intelligent, multifunctional MW absorbers aimed at adaptive EMI shielding in military and advanced electronic applications. Zhou et al. [259]. fabricated a 3D rGO/MXene/FeCoC hybrid aerogel that exhibits strong EM wave attenuation, achieving a minimum reflection loss of −61.4 dB and an EAB of 4.95 GHz at a thickness of only 1.55 mm (Figure 28c). The interconnected porous framework promotes multiple internal reflections of incident waves, while the synergistic combination of graphene, MXene, and magnetic FeCoC NPs provides balanced dielectric and magnetic loss mechanisms. Compared with single‐component absorbers, this multicomponent architecture offers improved impedance matching and enhanced energy dissipation. The study demonstrates an effective strategy for designing high‐performance MW absorbers by integrating multidimensional structures with multifunctional material components.

### 7.7 EMI Shielding

MXene‐based aerogels exhibit outstanding EMI shielding performance owing to their 3D frameworks that establish continuous electrically conductive networks, leading to significant ohmic dissipation. The presence of abundant surface functional groups and heterogeneous interfaces induces strong interfacial (Maxwell‐Wagner) polarization, which effectively converts incident EM energy into thermal energy. Hierarchically organized meso‐ and macro‐pores enhance impedance matching with free space, facilitating wave penetration into the aerogel rather than surface reflection [262, 263]. Once transmitted into the interior, the highly tortuous porous channels induce multiple scattering events and internal reflections, increasing the effective propagation path and strengthening attenuation. Consequently, EMI shielding in MXene aerogels is largely dominated by absorption mechanisms, thereby reducing secondary EM radiation compared with conventional metallic foils or dense films. Moreover, SE can be readily tuned over a wide frequency range by controlling parameters such as aerogel thickness, pore architecture, NSs orientation, and bulk density.

Incorporation of dielectric C materials such as CNTs or graphene, as well as magnetic components including Fe_3_O_4_ or Ni, introduces additional dipolar and magnetic loss mechanisms that broaden the effective shielding bandwidth and enhance SE. The resulting composites exhibit high specific shielding performance while maintaining mechanical flexibility, thermal robustness, and resistance to corrosion, making them well suited for wearable devices, aerospace structures, and compact electronic housings. These attributes position MXene‐based hybrid aerogels as promising materials for advanced applications in aerospace, defense, and portable electronics. The following section reviews recent progress in MXene hybrid aerogels, highlighting innovative structural designs, EMI shielding performance, and multifunctional properties, with particular emphasis on lightweight, mechanically resilient, and environmentally sustainable architectures [264, 265].

**FIGURE 29:**
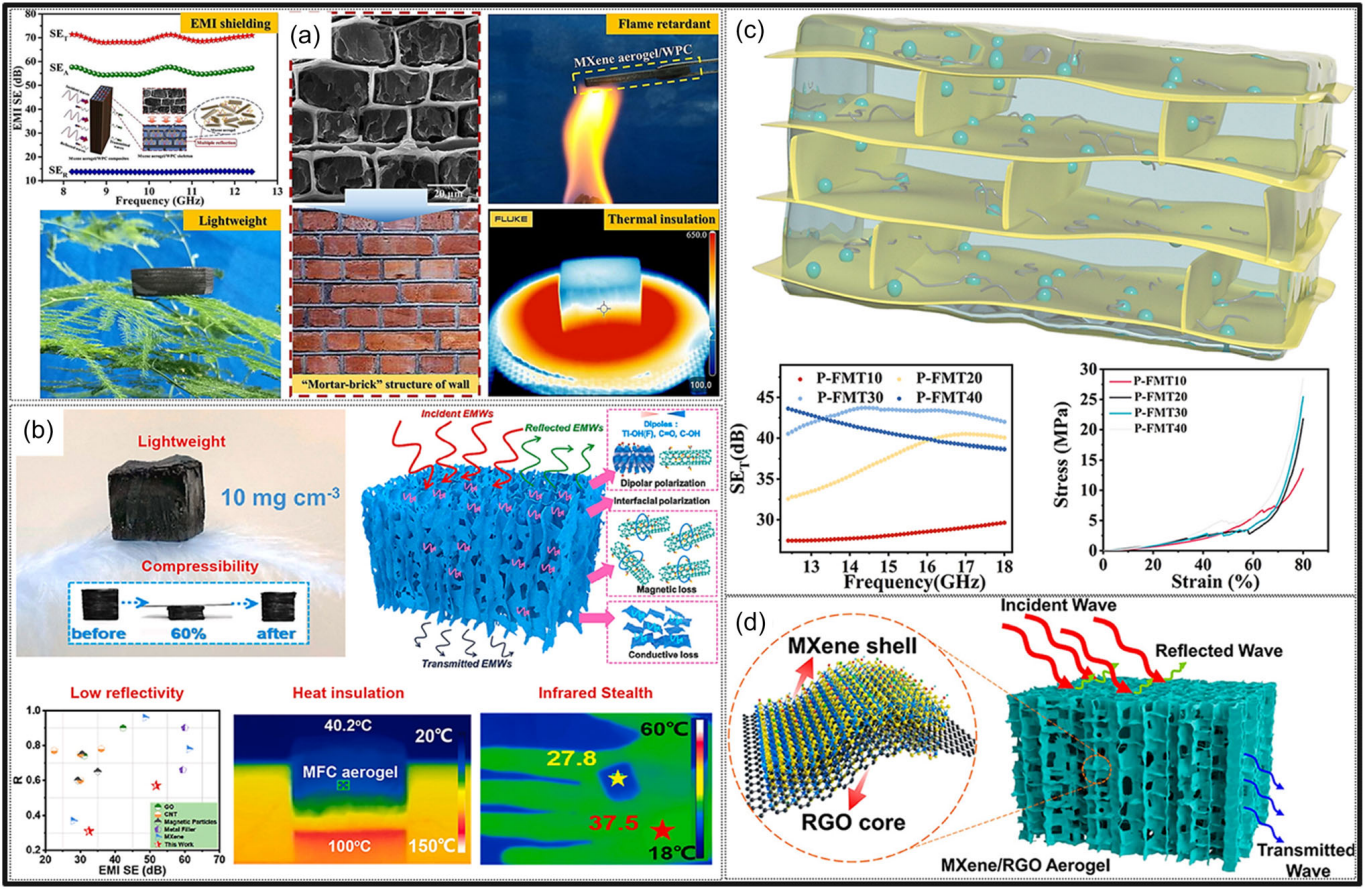
(a) MXene aerogel combined with WPC to form ultralight, wall‐like brick‐mortar structures for enhanced EMI protection. Adapted from [266]. Copyright 2020, Elsevier B.V. (b) Magnetic, multifunctional MXene hybrid aerogels designed to suppress EMI while limiting EM reflection. Adapted from [267]. Copyright 2023, Elsevier B.V. (c) High‐strength MXene hybrid aerogel suitable for diverse applications, including EMI shielding. Adapted from [268]. Copyright 2024, Elsevier B.V. (d) Highly efficient EMI‐shielding behavior achieved in 3D MXene/rGO composite aerogel architectures. Adapted from [269]. Copyright 2018, American chemical society.

Wood‐derived porous C (WPC) frameworks have been employed as scaffolds for constructing ultralight MXene aerogel/WPC hybrids by leveraging the intrinsic hierarchical architecture of wood (Figure 29a). When MXene aerogels are integrated into the honeycomb‐like microchannels of WPC, a brick‐and‐mortar configuration is formed, in which MXene acts as the “brick” and WPC as the “mortar.” This architecture is highly effective for EMI shielding, as it enhances structural integrity, prolongs EM wave propagation paths, and promotes conversion of incident radiation into thermal and electrical energy. As a result, the composite achieves a SE of 71.3 dB at an ultralow density of 0.197 g cm^−3^. Increasing the carbonization temperature further improves graphitization and electrical conductivity. In addition, the composite exhibits anisotropic compressive strength, low thermal conductivity, and flame resistance, making it suitable for demanding service environments [266].

Magnetic MXene/Fe_3_O_4_@acidified‐MWCNT hybrid aerogels were prepared via directional freezing followed by freeze‐drying, producing ultralight structures with tunable EMI SE ranging from 32.5 to 51.6 dB in the X‐band at an extremely low density of 10 mg cm^−3^ (Figure 29b). The preloading of Fe_3_O_4_ NPs onto acid‐treated MWCNTs strengthens interfacial polarization and magnetic loss, leading to a reduced power reflection coefficient of 0.31. Beyond effective EMI shielding, these aerogels also display reversible compressibility, excellent fatigue durability, thermal insulation, and infrared camouflage properties, highlighting their potential for sustainable EMI shielding and infrared stealth applications [267]. To overcome limitations associated with high EM reflection and limited mechanical strength in Ti_3_C_2_T_*x*_ aerogels, PDMS‐encapsulated Fe_3_O_4_/MWCNT/Ti_3_C_2_T_*x*_ aerogels were fabricated using a combination of bidirectional freeze‐drying and vacuum impregnation (Figure 29c). The resulting lamellar porous structure, together with the synergistic effects of conductive, dielectric, and magnetic components, achieved an EMI SE of 43.6 dB across the X‐ and Ku‐bands, while restricting reflection loss to 7 dB (16.7% of total shielding). PDMS incorporation significantly improved compressive strength (28.62 MPa), tensile strength (1.67 MPa), and thermal insulation performance, thereby expanding the material's practical applicability [268].

A GO‐assisted hydrothermal process combined with directional freezing and freeze‐drying has enabled the fabrication of lightweight yet electrically conductive 3D MXene scaffolds (Figure 29d). The resulting MXene/graphene composite aerogels exhibit highly ordered cellular structures, in which graphene forms the internal supporting framework while MXene constitutes the cell walls. Owing to this architecture, the aerogels achieve a high conductivity of 1085S m^−1^ and an EMI SE exceeding 50 dB in the X‐band with a low MXene loading of only 0.74 vol %. This approach expands the practical applicability of MXene‐based composites in EMI shielding, energy storage, and sensing technologies [269]. In a separate study, Abdul and coworkers [270] reported a ultralight, nonmagnetic MXene/PI (MXPI) aerogel fabricated via a straightforward freeze‐drying approach, in which PI served as a structural binder to suppress MXene restacking and enhance intersheet interactions. The MXPI‐25% sample demonstrated excellent EM wave absorption, achieving a minimum reflection loss of −64.70 dB at 13.92 GHz with an EAB of 1.04 GHz at a thickness of 5.24 mm. The outstanding absorption performance was attributed to the combined effects of conductive loss and multiple polarization relaxation mechanisms. Furthermore, the porous aerogel framework and uniformly distributed MXene NSs improved impedance matching and EM attenuation, highlighting the material's potential for EMI shielding and stealth‐related applications.

**FIGURE 30:**
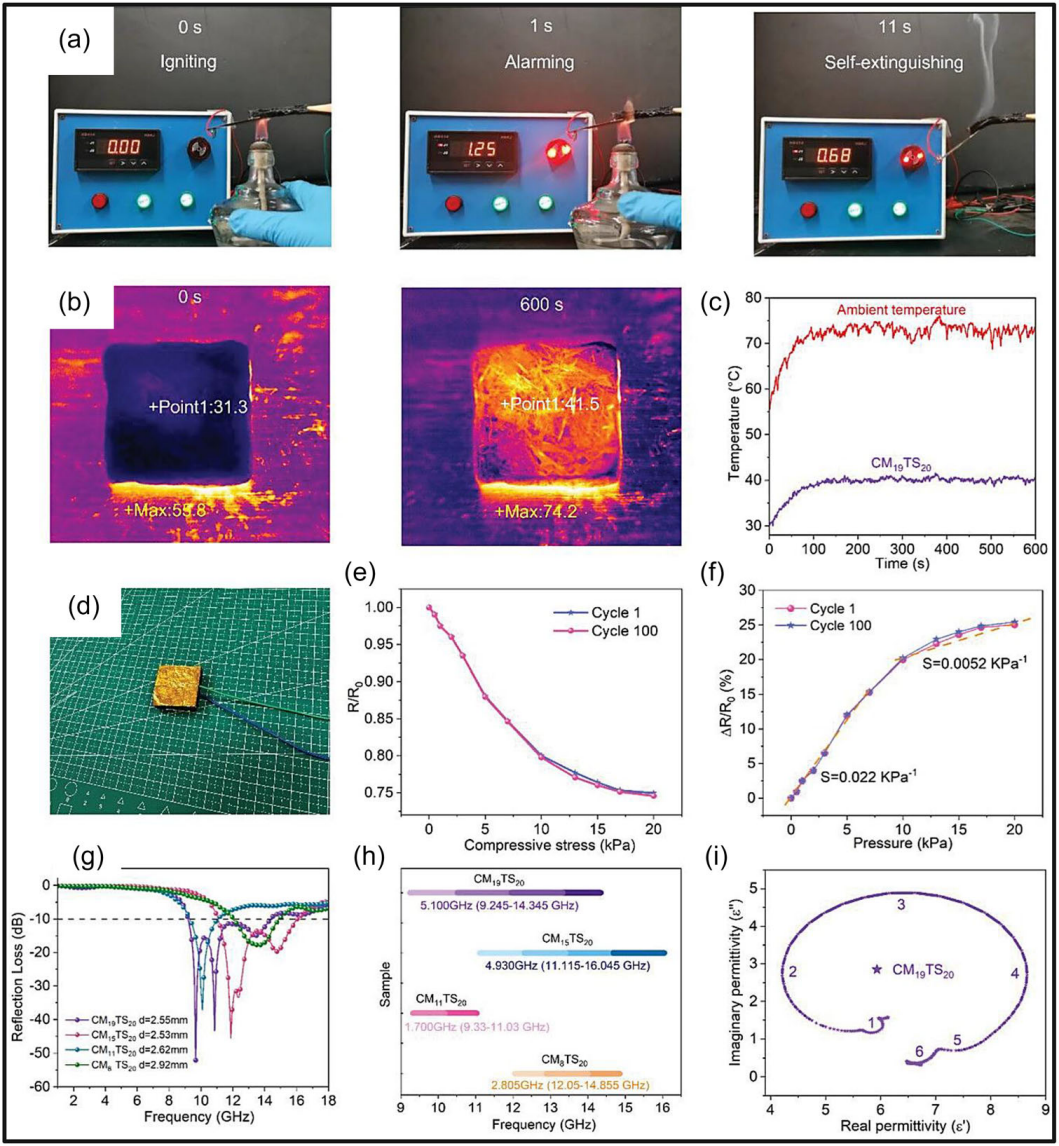
Demonstration of multifunctional properties of MXene‐based hybrid aerogels. (a) Time‐resolved snapshots from fire‐warning experiments highlighting rapid alarm activation and flame‐retardant behavior. (b) Thermal infrared snapshots of CM_19_TS_20_ obtained at 0 and 600 s under a heating temperature of 80°C, revealing excellent insulation performance. (c) Comparison of temperature variations on the aerogel surface and the heating stage. (d) Pictorial demonstration of pressure sensor fabrication process. (e) Cyclic resistance response (R/R_0_) at 20% compressive strain for 100 cycles. (f) Pressure‐dependent electrical response of CM_19_TS_20_. (g) Reflection loss curves of CM_*x*_TS_20_ composites. (h) EAB of CM_*x*_TS_20_ in X‐ and Ku‐bands. (i) Cole–Cole semicircle plots characterizing the dielectric behavior of CM_19_TS_20_. Adapted from [114]. Copyright 2023, Wiley‐VCH.

Owing to its tailored aerogel architecture, the CM_19_TS_20_ hybrid exhibits a broad range of multifunctional characteristics that enable diverse applications. As shown in Figure 30a, the CM_19_TS_20_ aerogel triggers a fire alarm within 1 second after ignition and self‐extinguishes in less than ten seconds, demonstrating outstanding flame‐retardant behavior and rapid fire‐warning capability. The material exhibits an exceptionally low thermal conductivity of 0.047 W m^−1^ K^−1^, which underpins its excellent thermal insulation capability (Figure 30b,c). Furthermore, CM_19_TS_20_ demonstrates reliable pressure‐sensing behavior, maintaining stable resistance responses over repeated compression‐release cycles (Figure 30d‐f), indicating its suitability for wearable pressure sensor applications. In addition to these functionalities, the aerogel shows strong EM wave attenuation, achieving a maximum absorption of 52.06 dB at 9.67 GHz (Figure 30g, h). As depicted in Figure 30i, its EMI shielding performance is attributed to the combined effects of dielectric loss, dipolar polarization, and interfacial polarization. Collectively, these results offer important insights for the development of advanced multifunctional EMI shielding materials and devices [114]. In summary, MXene aerogels have emerged as highly promising candidates for EMI shielding applications. Their distinctive 2D layered architectures combined with high electrical conductivity enable strong EMI SE, typically exceeding 50 dB, while maintaining ultralow density. The incorporation of porous and composite architectures further enhances EM wave attenuation by promoting absorption and suppressing reflection, which is particularly advantageous for aerospace and defense applications. Moreover, the integration of additional functionalities, like thermal insulation, flame retardancy, and mechanical strength, positions MXene aerogels as next‐generation solutions for advanced EMI shielding materials.

### 7.8 Catalyst Applications

MXene‐based aerogels have recently emerged as versatile catalytic materials that combine highly active surfaces with macroscopic, reactor‐compatible scaffolds. Their 3D, porous, and electrically interconnected frameworks provide a high density of accessible catalytic sites while suppressing mass‐ and charge‐transport limitations, a synergy that supports high catalytic turnover in energy‐ and environment‐related processes. These materials have demonstrated outstanding performance in HER and competitive activity toward ORR in metal‐air batteries (MABs) and fuel cells. In addition, MXene aerogels facilitate pollutant degradation through advanced oxidation processes, enable fast nitrophenol degradation, and promote CO_2_ reduction toward value‐added chemicals [271, 272]. Moreover, defect‐ and surface‐termination engineered Ti_3_C_2_T_*x*_ aerogels, often incorporating single atoms or nanoclusters, display emerging potential for N_2_ reduction to NH_3_ under ambient conditions.

**FIGURE 31:**
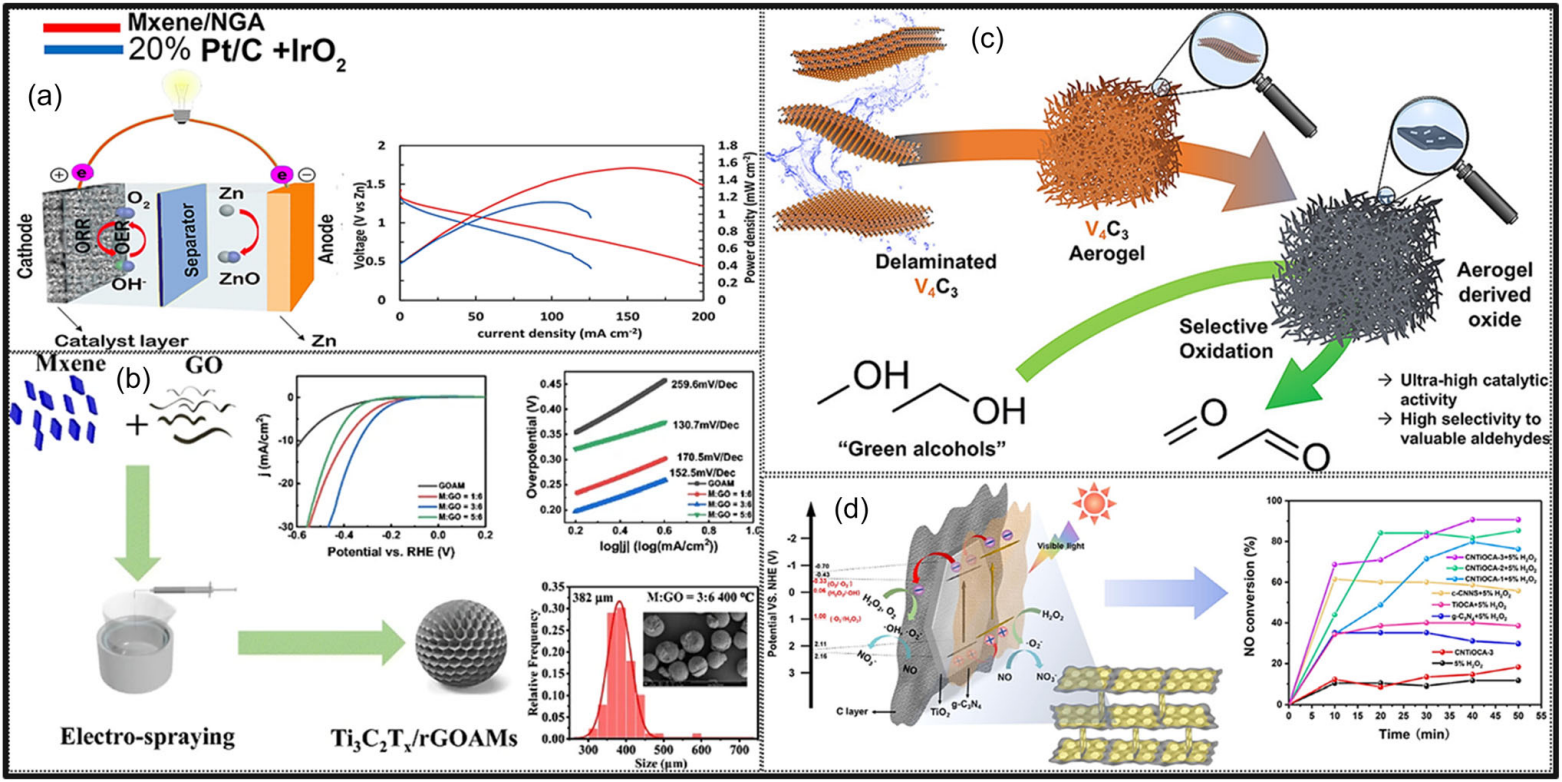
Catalytic functionalities enabled by MXene aerogels. (a) Schematic illustration of preparation of N‐doped graphene/Ti_3_C_2_T_*x*_ aerogel exhibiting high ORR activity in fuel cells and MABs. Adapted from [273]. Copyright 2021, Elsevier B.V. (b) MXene/rGO aerogel microspheres employed as efficient HER electrocatalysts. Adapted from [274]. Copyright 2021, Springer Nature. (c) Highly active vanadia catalyst derived from MXene aerogels for selective alcohol upgrading. Adapted from [275]. Copyright 2023, American chemical society. (d) 3D hierarchical porous MXene‐derived TiO_2_@C modified with g‐C_3_N_4_ for combined photocatalytic and H_2_O_2_‐assisted NO removal. Adapted from [276]. Copyright 2022, Elsevier B.V.

From a mechanistic perspective, adjustable surface terminations, interlayer spacing, and engineered heterointerfaces govern adsorption energetics and reaction routes, while meso‐ and macro‐porous architectures reduce diffusion distances and inhibit NSs restacking. The resulting synergy among structure, surface chemistry, and transport properties establishes MXene aerogels as highly adaptable catalytic platforms. Critical design parameters include termination regulation, incorporation of heteroatoms or single‐atom species, and rational pore‐structure engineering, with improvements in durability and selectivity representing key challenges for large‐scale implementation [271]. MXene aerogels have been widely explored as electrocatalysts for ORR, HER, N_2_ reduction (NFR), and CO_2_RR. For instance, N‐doped graphene integrated with Ti_3_C_2_T_*x*_ exhibited ORR behavior comparable to commercial Pt/C, achieving an onset potential of 1.003 V versus RHE and a current density of 5.65 mA cm^−2^, along with superior durability in fuel cells and ZABs (Figure 31a) [273]. In addition, vertically aligned MXene/CNT aerogels decorated with Pt clusters delivered HER activity at industrially relevant current densities, requiring overpotentials of 208 and 249 mV at 0.5 and 1 A cm^−2^, respectively [277]. Similarly, MXene‐graphdiyne NTs‐MoS_2_ hybrid aerogels showed outstanding HER performance (η_10_ = 109 mV), attributed to synergistic coupling between the conductive scaffold and edge‐rich MoS_2_ domains [278].

MXene/rGO aerogels further enhanced HER kinetics by facilitating improved mass transport, while NiSe_2_‐decorated MXene/rGO aerogels exhibited bifunctional electrocatalytic behavior, delivering low overpotentials of 97 mV for HER and 262 mV for OER (Figure 31b) [274]. For NFR, S‐doped MXene aerogels incorporating metal sulfides achieved a NH_3_ production rate of 12.4 μg h^−1^ mg^−1^ cat with a Faradaic efficiency of 27.05% at −0.15 V [279]. In CO_2_RR, MXene aerogels templating bimetallic Cu‐Pd catalysts reached over 90% selectivity toward formate with an energy efficiency of 47%, representing the highest value reported so far for membrane‐electrode assembly (MEA) configurations [280]. Collectively, these studies highlight how compositional tuning and 3D architectural design can substantially boost the catalytic performance of MXene aerogels in electrochemical applications.

**FIGURE 32:**
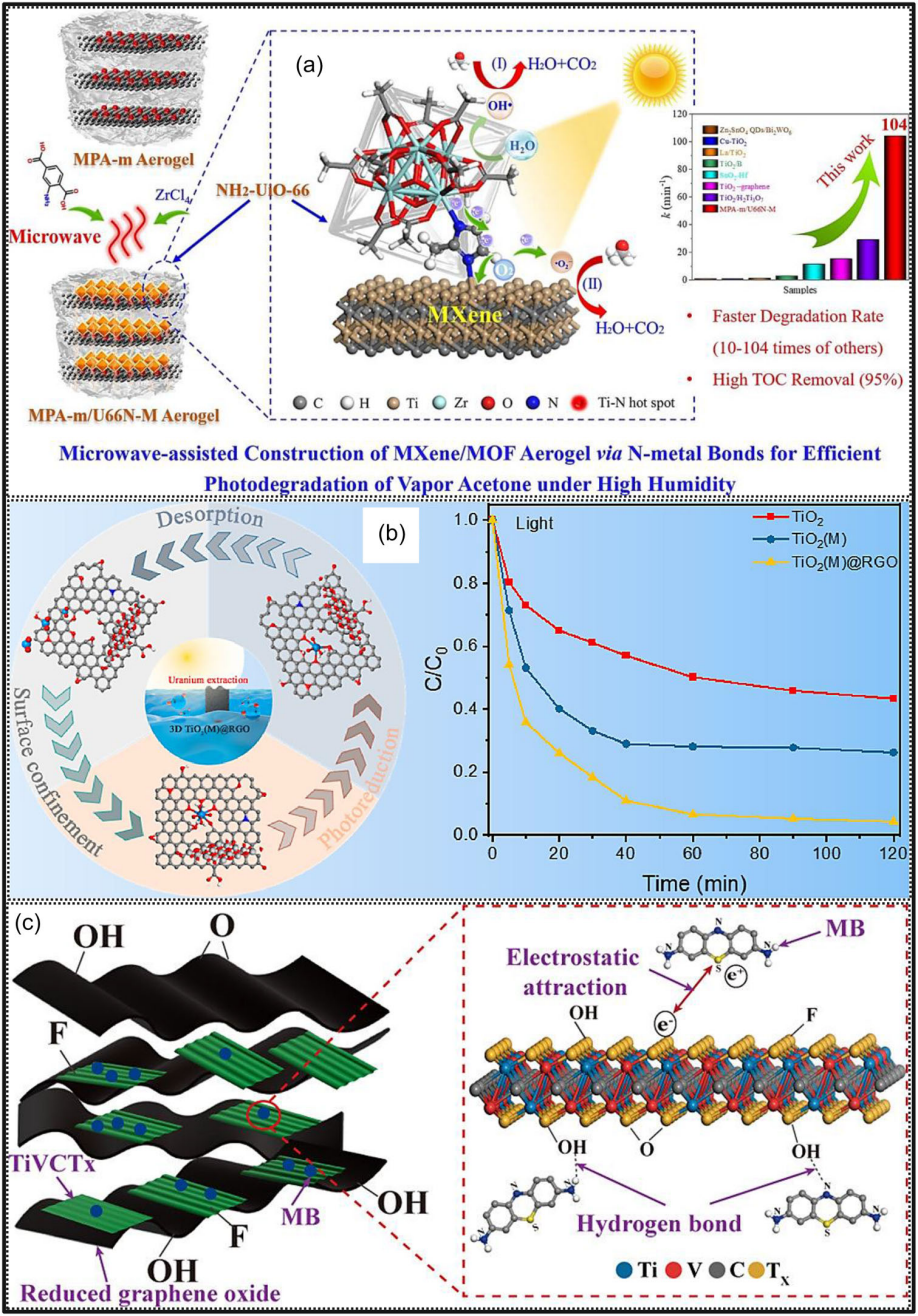
(a) MW‐assisted MXene/MOF aerogel featuring N‐metal bonding for high‐efficiency acetone vapor photodecomposition under humid conditions. Adapted from [281]. Copyright 2023, Elsevier B.V. (b) MXene‐derived, defect‐rich TiO_2_@rGO aerogel exhibiting ultrafast carrier transport for light‐driven U recovery, validated through experimental and theoretical investigations. Adapted from [282]. Copyright 2022, American chemical society. (c) TiVCT_*x*_/graphene aerogels showing strong performance in organic pollutants removal from wastewater. Adapted from [171]. Copyright 2024, American chemical society.

Beyond their electrocatalytic roles, MXene‐based aerogels have gained increasing attention as versatile platforms for photocatalytic and light‐driven reactions. For example, vanadium oxide aerogels derived from V_4_C_3_T_11_ MXenes exhibit alcohol oxidation activities during methanol and ethanol conversion that are nearly an order of magnitude greater than those of conventional catalysts (Figure 31c) [275]. In a related study, g‐C_3_N_4_/TiO_2_@C aerogels achieved a NO removal efficiency of 90.7% through the synergistic effects of photocatalysis and H_2_O_2_‐assisted oxidation (Figure 31d) [276]. Under optimized humidity conditions, MXene/MOF aerogels enabled up to 97.3% photodegradation of acetone, which was attributed to improved light absorption and efficient charge separation facilitated by N‐metal interactions (Figure 32a) [281]. Additionally, TiO_2_/Ti_3_C_2_ aerogels demonstrated 98.3% degradation efficiency toward palm oil mill effluent under simulated conditions without external aeration [283]. Another high‐efficiency photocatalytic system, ZIF‐67@MXene‐TA‐CNF aerogels, demonstrated near‐complete removal of tetrabromobisphenol A (TBBPA), achieving 99.9% removal with 81.8% total organic C (TOC) mineralization within 40 min. This performance is attributed to the generation of reactive radical species through PMS activation under visible‐light irradiation [284]. In parallel, solar‐driven PPy/BiVO_4_‐PI/MXene aerogels integrated photothermal evaporation with photocatalytic functionality, producing an evaporation rate of 1.64 kg m^−2^ h^−1^ and dye removal efficiencies above 70%, while simultaneously enabling effective salt recovery [285]. Additionally, MXene‐derived TiO_2_/rGO aerogels demonstrated effective photo‐assisted uranium (U) extraction, reaching 95.7% removal efficiency owing to enhanced electron transport via Schottky junction formation (Figure 32b) [282]. TiVCT_*x*_/graphene aerogels further exhibited excellent adsorption performance toward dyes and pharmaceutical contaminants, achieving MB uptake as high as 319.67 mg g^−1^ and broad‐spectrum pollutant removal (Figure 32c) [171].

In conclusion, MXene‐based aerogels represent a transformative materials platform for energy conversion, storage, and environmental remediation. Their hierarchical porosity and chemically adjustable surfaces enable the integration of diverse functions within a single architecture, allowing simultaneous high performance in ORR, HER, OER, NFR, CO_2_RR, photocatalysis, pollutant removal, and adsorption processes. Future efforts should prioritize scalable manufacturing strategies, long‐term structural robustness under demanding operational environments, and deeper mechanistic insights to facilitate rapid translation into industrial and environmental technologies. With continued advances, MXene aerogels are poised to become cornerstone materials in sustainable energy and environmental systems.

### 7.9 Thermal Regulation

MXene‐based aerogels present significant opportunities in thermal management owing to the combination of their intrinsically great thermal conductivity and ultralight, hierarchically porous architectures. Continuous Ti_3_C_2_T_*x*_ networks enable rapid lateral heat transport, while strong photothermal activity supports efficient conversion of light into heat for applications such as de‐icing, solar‐assisted heating, and Joule heating. Their mechanical strength and elastic recovery preserve thermal transport pathways during compression and repeated thermal cycling, enabling reliable operation in demanding environments [286, 287]. These aerogels can function either as independent heat‐dissipation components or be integrated with insulating and PCMs to provide simultaneous thermal transport and energy storage, while their conductive frameworks also deliver EMI shielding. Collectively, these properties make MXene aerogels attractive for thermal control in electronics and batteries, wearable heating systems, and solar energy technologies [30, 288].

Directional freeze‐casting was employed to fabricate an ultralight nano BC (BNC)/MXene aerogel with a density of 0.011 g cm^−3^. The BNC framework establishes a highly porous architecture (99.33%) and yields an exceptionally low thermal conductivity of 0.038 W m^−1^K^−1^. The resulting aerogel exhibits excellent photothermal performance, achieving 98% solar absorption across 0.25–2.5 μm, a rapid temperature rise to 88°C within 10 s, and sustained thermal stability at 89°C over 120 min. These characteristics highlight the potential of BNC/MXene aerogels for efficient thermal‐management solutions relevant to climate mitigation strategies [289]. Inspired by antifreeze beetles, a surface‐engineered MXene/BC aerogel was further infiltrated with an organic phase‐change material to form a composite film, significantly enhancing phase‐change enthalpy, mechanical robustness, and active material loading. A sub‐millimeter‐thick aerogel layer maintained a thermally comfortable state for over 800 s, demonstrating the effectiveness of bioinspired integration of photothermal conversion and thermal energy storage [290].

**FIGURE 33:**
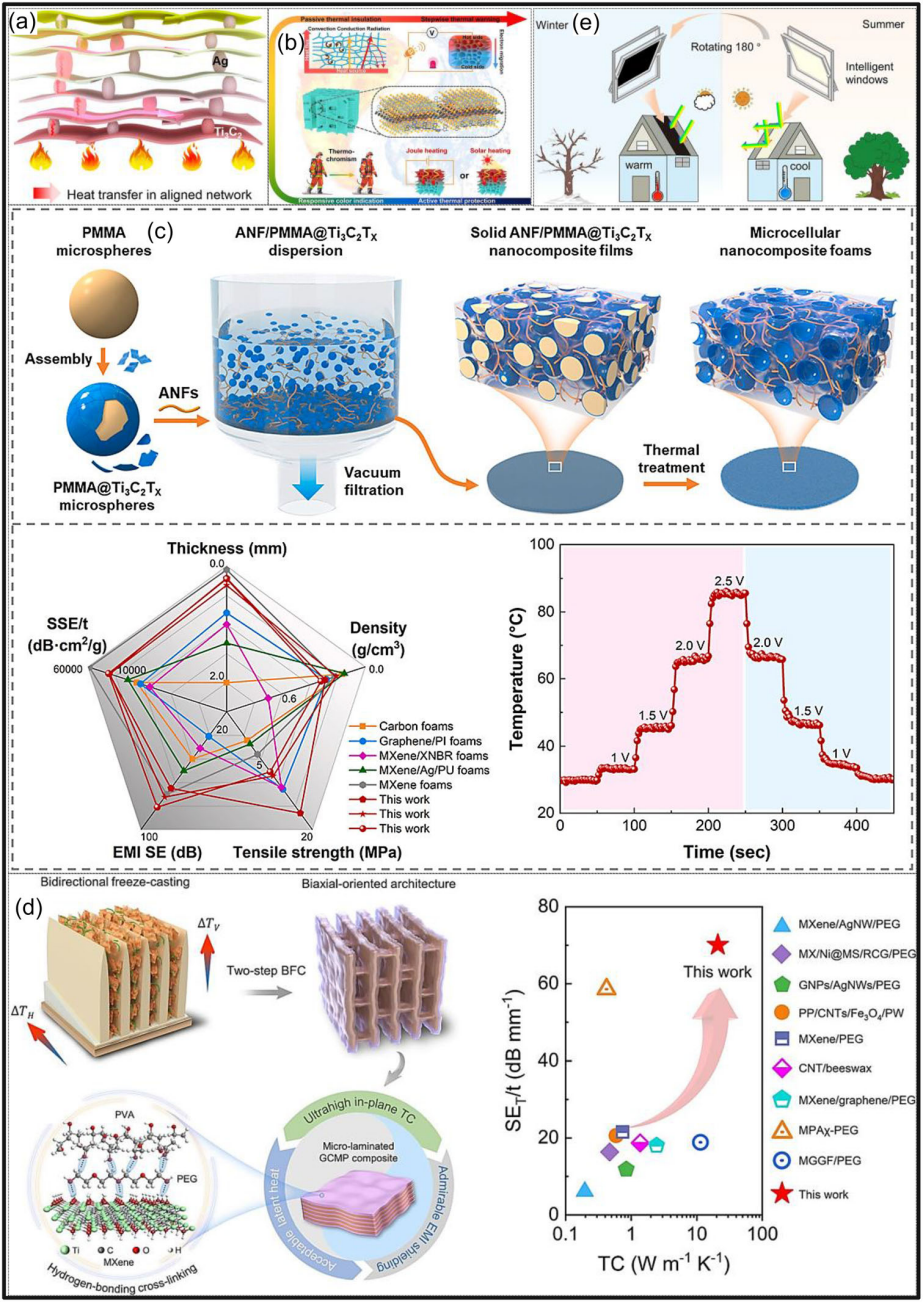
(a) Ice‐directed MXene/Ag epoxy hybrids demonstrating superior thermal transport performance. Adapted from [291]. Copyright 2020, American chemical society. (b) Hybrid anisotropic MXene/rGO/PEDOT:PSS aerogel for dual‐mode thermal protection and stepwise temperature alerting. Adapted from [292]. Copyright 2024, Elsevier B.V. (c) Low‐weight MXene‐based microcellular foams combining high strength with EMI shielding and thermal control in a single material system. Adapted from [293]. Copyright 2025, Elsevier B.V. (d) Multilayer‐oriented, millefeuille‐like phase‐change hybrid enabling efficient thermal regulation and EMI shielding. Adapted from [294]. Copyright 2024, Elsevier B.V. (e) MXene‐NH_2_/ANF aerogel providing flame resistance and adaptive thermal insulation for enhanced safety and energy savings across seasons. Adapted from [295]. Copyright 2024, American chemical society.

The 3D MXene/Ag aerogels with through‐thickness alignment were employed as thermally conductive frameworks within epoxy composites to mitigate interfacial heat‐transfer limitations in electronic devices. Decoration of MXene NSs with Ag NPs effectively reduced interfacial contact resistance, achieving an exceptionally low thermal boundary resistance of 4.5 × 10^−7^ m^2^ W K^−1^. At a filler content of 15.1 vol %, the composites exhibited a through‐plane thermal conductivity of 2.65 W m^−1^K^−1^, representing an enhancement of more than three orders of magnitude compared to pristine epoxy. As a result, these materials demonstrated excellent heat‐dissipation capability in practical electronic systems, including consumer laptops and smartphones (Figure 33a) [291]. In a related approach, an anisotropic MXene/rGO/PEDOT:PSS aerogel was engineered for thermal regulation across a wide temperature window (0–200°C), combining intrinsic microporous insulation with tunable photothermal and electrothermal heating for adaptive thermal control. The aerogel integrates thermochromic inks and thermoelectric outputs to enable real‐time temperature monitoring and automatic warning responses when critical thresholds are exceeded. Combined with its intrinsic flame‐retardant behavior, this multifunctional system offers enhanced operational safety and reliability in demanding environments (Figure 33b) [292]. In a related development, lightweight ANF/Ti_3_C_2_T_*x*_ hybrid foams (density ≈ 0.29 g cm^−3^) were engineered to deliver both EMI shielding and effective thermal regulation. These foams exhibit excellent SE (64.9 dB), high mechanical robustness (tensile strength of 16.5 MPa), and superior flame resistance. In addition, their tunable Joule‐heating capability enables stable and controllable thermal management, making them attractive for aerospace, electronic, and telecommunication applications (Figure 33c) [293].

An asymmetric ANF/MXene‐NH_2_ aerogel (ASAMA) was engineered to deliver energy‐efficient thermal regulation across both cold and hot environments. The asymmetric architecture integrates layers with contrasting thermal conductivities, leveraging the strong photothermal response of MXene‐NH_2_ alongside the high solar reflectance and mid‐infrared emissivity of ANF. As a result, ASAMA maintains an internal temperature of approximately 20°C at ‐5°C ambient conditions and reduces external temperatures of 40°C to about 28°C. The aerogel also exhibits high mechanical robustness (tensile strength of 2.98 MPa), effective thermal insulation over a wide temperature window (−10 to 280°C), and sustained flame resistance for up to 6 min, enabling low‐energy thermal control (Figure 33d) [294]. In parallel, MXene‐based aerogels with large SSA and high electrical conductivity were developed as flexible pressure sensors for health monitoring and artificial intelligence (AI) applications under extreme climatic conditions. Covalent cross‐linking between MXene NSs, silane monomers, and PDA‐modified SiO_2_ NFs significantly enhances mechanical integrity and elasticity. These sensors demonstrate high sensitivity (0.83 kPa^−1^), rapid response (48 milli seconds), and long‐term durability over 10 000 loading cycles (Figure 33e), enabling applications in motion tracking, biomedical diagnostics, and personal thermal regulation [295].

A leaf‐inspired biomimetic MXene@nanocellulose aerogel‐cotton composite was engineered for infrared stealth and personal thermal regulation. With a thickness of 2.6 mm, the fabric exhibits a high breaking strength of 158 N and low infrared emissivities of 0.35 in the 3–5 μm band and 0.22 in the 8–14 μm band. The material enables infrared concealment for up to 600 s and improves thermal performance, increasing radiative heating by 5°C and solar‐induced heating by 17°C compared with pristine cotton. These features highlight its promise for both defense‐related applications and wearable thermal regulation systems [296]. Flexible phase‐change hybrid films were produced via physical blending of paraffin, PVA, and MXene to simultaneously enhance mechanical robustness, photothermal efficiency, and thermal transport. Higher paraffin content increased latent heat storage (up to 117.02 J g^−1^) at the expense of tensile strength (1.01 MPa), whereas MXene incorporation significantly reinforced the films (2.51 MPa) while retaining high latent heat values (>92.72 J g^−1^). The resulting composites show stable and repeatable photothermal conversion, near‐complete shape recovery, and good thermal durability, demonstrating effective temperature‐regulation capability for thermal management applications [297].

**FIGURE 34:**
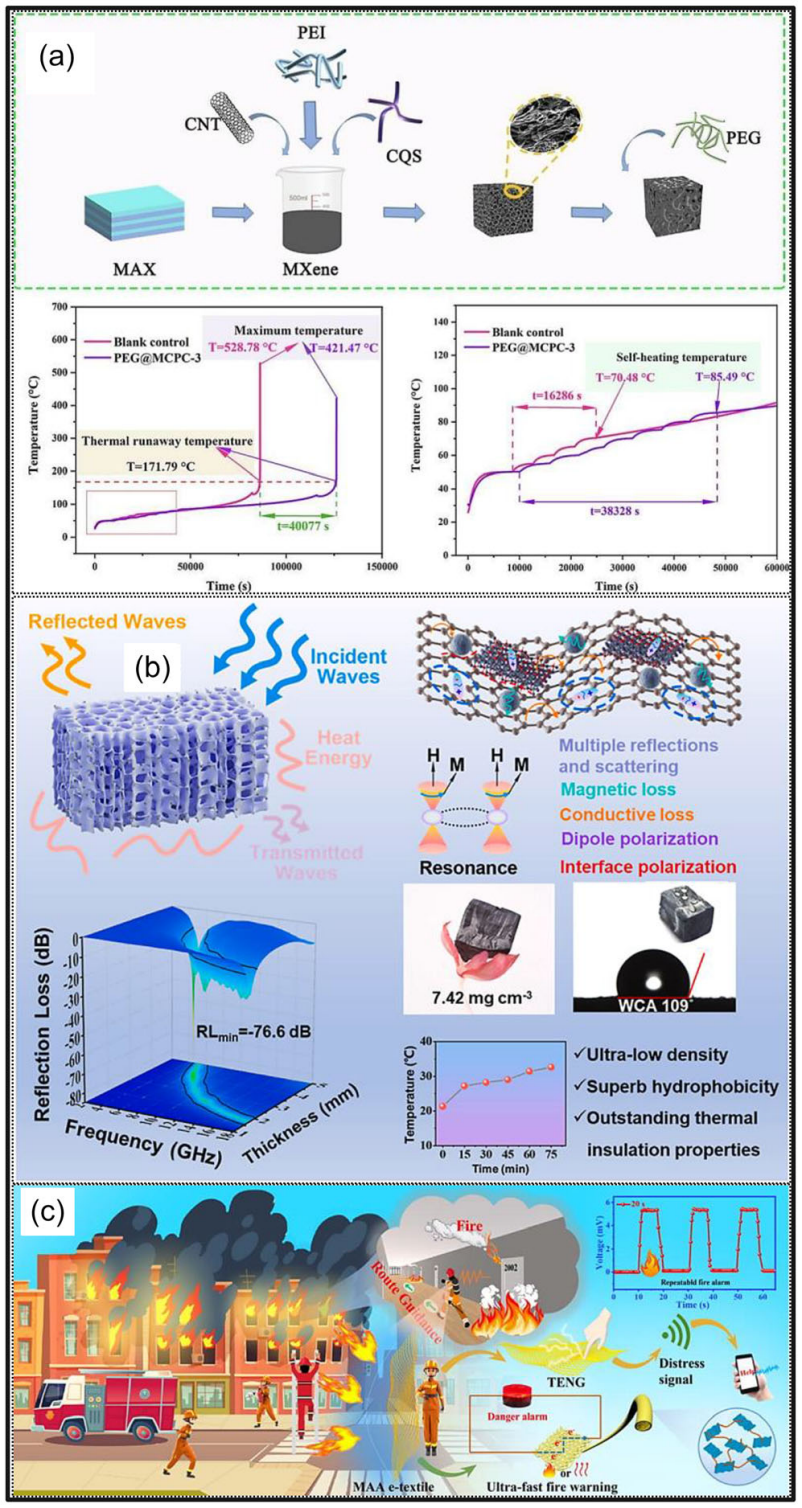
(a) Schematic illustration of MXene‐based aerogel derived PCMs designed for mitigating thermal runaway in battery systems. Adapted from [299]. Copyright 2025, Elsevier B.V. (b) Ultralight Fe_3_O_4_@SiO_2_/Ti_3_C_2_T_*x*_/rGO magnetic aerogels exhibiting combined EM wave absorption and thermal regulation functionality. Adapted from [300]. Copyright 2024, Elsevier B.V. (c) Self‐powered fire‐alert e‐textile fabricated from MXene, Ag NWs, and ANF aerogel fibers for integration into firefighting garments. Adapted from [301]. Copyright 2023, Elsevier B.V.

A Janus‐type MXene NFs aerogel with ultrahigh porosity (98.2%) and notable mechanical robustness (tensile strength of 2 MPa) has been developed to enable switchable radiative heating and cooling. When applied to a model house featuring asymmetric functional layers, the aerogel maintains indoor temperatures above 25°C in winter and below 30°C in summer. This architecture enables energy‐efficient thermal regulation across diverse climatic conditions, while its macroscopic formability enhances practicality for real‐world deployment [298]. In a related study, a multifunctional MCPC aerogel framework composed of quaternized chitosan, PEI, CNTs, and MXene was engineered as a host matrix for PCMs. The resulting composite exhibits high enthalpy efficiency, enhanced thermal conductivity, and effective EMI shielding, thereby delaying battery thermal runaway. Moreover, the aerogel supports photothermal, electrothermal, and magnetothermal energy conversion, extending the applicability of PCMs in advanced thermal management systems (Figure 34a) [299]. A microlaminated graphene‐C/MXene‐coated PCMs (GCMP) with a millefeuille‐like architecture has been fabricated via bidirectional freeze‐casting, delivering high in‐plane thermal conductivity of 20.96 W m^−1^K^−1^ along with an EMI SE of 70.5 dB. The composite also exhibits a high latent heat density of 153.8 J cm^−3^, outstanding leakage resistance, and strong thermal cycling stability. These combined properties make GCMP well suited for high‐efficiency thermal management in microelectronic devices and solar‐thermoelectric energy conversion systems, with promising prospects in electronics, energy storage, and aerospace applications [302].

Wang and coworkers [300] reported the fabrication of porous Fe_3_O_4_/SiO_2_/Ti_3_C_2_T_*x*_/rGO aerogels, which, at a filler loading of 5 wt %, exhibited an outstanding minimal reflection loss of −76.6 dB and an EAB of 5.36 GHz. The lowweight structure, with a density of only 7.42 mg cm^−3^, was achieved through directional freeze‐drying followed by thermal reduction. These aerogels display strong hydrophobicity, effective thermal insulation, and robust stability under elevated temperature and humid conditions, making them suitable for EM wave absorption and thermal regulation applications (Figure 34b). In a separate study, MXene/K^+^/paraffin wax composites were produced using metal‐ion‐assisted self‐assembly and vacuum impregnation, achieving a solar‐thermal conversion efficiency of 98.4%. The MK3@PW system enables conversion of solar, electrical, and magnetic energy into heat, generating an output voltage of 206 mV via solar‐thermal‐electric coupling. It also demonstrates high Joule heating capability (105°C at 2.5 V) and strong magnetothermal behavior, while providing EMI SE of 57.7 dB, highlighting its multifunctional PCM characteristics [303].

Yu's group [301] reported a wearable, self‐powered fire early‐warning electronic textile (MAA e‐textile) fabricated by wet spinning Ti_3_C_2_T_*x*_, Ag NWs, and ANF aerogel fibers (Figure 34c). Integrated into firefighter protective clothing, the textile enables broad‐range temperature monitoring from 100 to 400°C by exploiting the thermoelectric response of MXene, triggering fire alerts within 1.6 s to warn personnel prior to garment failure. The cooperative interaction between MXene and ANFs significantly enhances flame resistance, reducing the peak heat release rate by approximately 80%. Moreover, the MAA e‐textile can power a TENG‐based localization system, supporting rescue operations for trapped firefighters. This strategy demonstrates a new paradigm for self‐powered fire safety monitoring in protective textiles. Overall, MXene‐based composite aerogels show strong potential as versatile platforms for thermal management across diverse applications, including electronics, aerospace, and personal thermal regulation. Their combination of high thermal conductivity, efficient photothermal conversion, mechanical robustness, and EMI shielding highlights the promise of organic–inorganic hybrid nanostructures in addressing extreme climate adaptation and energy‐efficient structural design.

## 8 Future Perspectives

MXene‐based aerogels are advancing rapidly due to the convergence of data‐driven materials design, multifunctional composite structures, and environmentally responsible fabrication strategies. Automated experimentation combined with ML now enables efficient exploration of the vast compositional space of MXene hybrids, facilitating accurate property prediction and accelerating materials discovery [304, 305]. At the same time, MXenes are increasingly combined with other 2D materials, biopolymers, and MOFs to form aerogel systems that inherently exhibit mechanical robustness, high electrical conductivity, and versatility for energy storage, sensing, and emerging applications. Scalable fabrication routes for lightweight, monolithic aerogels, such as ambient‐pressure drying, additive manufacturing, and chemical foaming, are being developed without excessive energy input, while greener synthesis methods, including HF‐free electrochemical etching, reduce environmental impact and promote industrial translation [306]. As a result, MXene aerogels are expanding beyond traditional uses in EMI shielding and electrochemical storage toward soft robotics, wearable electronics, solar‐driven desalination, and neuromorphic systems [307]. Supported by advanced design methodologies and a strong focus on sustainability, MXene aerogels are poised to evolve into adaptable and programable materials platforms bridging fundamental research and practical deployment.

1. Future research may increasingly employ advanced AI and ML frameworks to rationally design MXene aerogel compositions. These tools enable rapid and cost‐effective prediction, tuning, and optimization of aerogel characteristics, significantly shortening development cycles. However, their practical implementation will require the development of high‐quality, standardized experimental datasets and closer integration with scalable manufacturing processes to ensure real‐world applicability.
2. The development of composite MXene aerogels incorporating complementary functional materials, such as MOFs, biopolymers, and other 2D systems, represents a promising research direction. These hybrid architectures can synergistically integrate MXenes’ high electrical conductivity with the mechanical robustness, hierarchical porosity, or catalytic functionality of secondary components. Future studies may further explore interfacial engineering and hierarchical design to simultaneously optimize performance, stability, and multifunctionality.
3. From an industrial perspective, advancing scalable fabrication routes, such as ambient‐pressure drying and additive manufacturing, is critical for the large‐scale production of MXene aerogels. Future efforts should prioritize refining these methods to enable precise control over complex architectures while maintaining high functional performance. In addition, replacing high‐cost processes such as supercritical drying with energy‐efficient alternatives, along with improving batch‐to‐batch reproducibility, will be essential for reliable and economically viable mass production.
4. Future research should emphasize the development of environmentally benign synthesis pathways for MXene aerogels to improve their sustainability profile. In particular, replacing hazardous etchants such as HF acid with safer alternatives, such as electrochemical etching, offers a promising route. Continued refinement of these green approaches for both MXene production and aerogel assembly could substantially reduce environmental impact and facilitate broader technological adoption.
5. Owing to their intrinsic elasticity and high electrical conductivity, MXene aerogels are promising candidates for future integration into soft robotic systems, wearable electronics, and bioelectronic devices. However, practical deployment will require addressing key engineering challenges, including long‐term mechanical reliability under repeated deformation, stable electrical interfacing with device components, and durability under real operating conditions. These materials can function as deformable electrodes, pressure sensors, and actuators, enabling improved comfort, responsiveness, and signal fidelity in wearable platforms.
6. MXene aerogels are expected to play an increasingly pivotal role in sustainable water treatment technologies. Future investigations may focus on enhancing their photothermal conversion efficiency to support solar‐driven desalination under demanding environmental conditions. At the same time, improving long‐term environmental stability, particularly resistance to oxidation and performance degradation in aqueous and oxygen‐rich environments, will be critical for their practical implementation.
7. Looking ahead, MXene aerogels are likely to find applications in neuromorphic computing and flexible memory technologies. Their combination of high electrical conductivity, mechanical compliance, and tunable surface chemistry makes them suitable for memristive devices and other components used in brain‐inspired computing architectures. Addressing issues related to device integration, stability, and reproducibility will be key to translating these concepts into functional systems.
8. Establishing standardized performance metrics will be critical for accelerating the transition of MXene aerogels from laboratory research to practical implementation. Future studies should focus on developing unified testing protocols for key parameters, such as EMI SE and sensor sensitivity, to enable meaningful cross‐comparisons among materials. In parallel, any biomedical application will require thorough evaluation of long‐term biocompatibility, in vivo degradation behavior, and immune responses to ensure clinical safety and efficacy.
9. Continued efforts are expected to further optimize MXene aerogels for established application areas, including energy storage, EMI shielding, and catalysis. Future research may focus on improving energy density, operational stability, and cycling durability through compositional tuning and architectural refinement. In addition, enhancing long‐term environmental durability and resistance to oxidation will be essential to ensure sustained performance under realistic working conditions.

## 9 Conclusion

In this review, we have systematically summarized the recent advances in MXene‐based aerogels, highlighting the intricate relationships between material composition, structural design, and multifunctional performance. By integrating insights from synthesis strategies, pore architecture engineering, surface chemistry, and hybridization approaches, it becomes evident that MXene aerogels represent a uniquely versatile class of materials capable of addressing diverse challenges across energy storage, environmental remediation, and EMI shielding.

Beyond their individual performance metrics, a central theme emerging from this review is the importance of structure‐property integration across multiple length scales, where nanoscale surface chemistry, mesoscale pore architecture, and macroscale mechanical integrity collectively determine functionality. While significant progress has been achieved in tailoring these features, the field is now approaching a critical stage where further advances will depend less on incremental material optimization and more on overcoming fundamental translational barriers.

In this context, several key “tipping points” can be identified for the future development of MXene aerogels. First, the realization of scalable and cost‐effective manufacturing routes remains essential, particularly through the replacement of energy‐intensive processes such as supercritical drying with more practical alternatives. Second, ensuring long‐term environmental stability, especially resistance to oxidation and performance degradation under realistic operating conditions, is crucial for maintaining functional reliability. Third, successful integration into real‐world devices will require addressing engineering challenges such as mechanical durability, interfacial compatibility, and reproducible large‐scale fabrication.

Furthermore, the continued evolution of MXene aerogels will benefit from the convergence of rational structural design, hybrid material integration, and data‐driven optimization approaches, enabling the development of materials with programable properties tailored to specific applications. Importantly, future research must increasingly bridge the gap between laboratory‐scale demonstrations and industrial requirements by incorporating standardized performance evaluation, lifecycle considerations, and application‐oriented design principles.

Looking forward, MXene aerogels are poised to transition from promising laboratory materials to functional components in next‐generation technologies. Achieving this transition will depend on coordinated advances in materials chemistry, process engineering, and device integration. By addressing these critical challenges, MXene aerogels have the potential to evolve into a foundational platform for lightweight, multifunctional materials in a wide range of technological applications.

## Funding

This work was supported by National Research Foundation of Korea (NRF) (Grants RS‐2024‐00449682, RS‐2024‐00433118 and RS‐2023‐00236572).

## Conflicts of Interest

The authors declare no conflicts of interest.

## Data Availability

Data sharing not applicable to this article as no datasets were generated or analyzed during the current study.
